# Synergistic degradation of fucoidans in the ocean

**DOI:** 10.1038/s41586-026-10980-z

**Published:** 2026-08-26

**Authors:** Andreas Sichert, Shaul Pollak, Taylor Priest, Akshit Goyal, Samuel Miravet-Verde, Shinichi Sunagawa, Otto X. Cordero, Uwe Sauer

**Affiliations:** 1 Institute of Molecular Systems Biology, ETH Zürich, Zürich, Switzerland; 2 Department of Civil and Environmental Engineering, Massachusetts Institute of Technology, Cambridge, MA USA; 3 Centre for Microbiology and Environmental Systems Science, Department of Microbiology and Ecosystem Science, Division of Microbial Ecology, University of Vienna, Vienna, Austria; 4 Institute of Microbiology, Department of Biology, ETH Zürich, Zürich, Switzerland; 5 International Centre for Theoretical Sciences, Tata Institute of Fundamental Research, Bengaluru, India

**Keywords:** Microbial ecology, Glycobiology

## Abstract

Fucoidans, a class of complex polysaccharides produced by brown algae and diatoms, contribute to long-term carbon sequestration owing to their resistance to microbial degradation^1,2^. Although individual microorganisms can break down portions of these polysaccharides^3–5^, it remains unclear whether complete breakdown is possible in nature and, if so, by what mechanisms. Here we show that fucoidans are degraded through synergistic interactions between specialized bacteria with complementary metabolic functions. Using metabolomic analysis of a reconstructed marine consortium, we uncovered metabolic guilds of bacteria that preferentially degrade either the sulfated fucose backbone or the side branches of rare monomers. This functional division of labour leads to an unexpectedly high number of synergistic interactions between different degraders that enhanced degradation efficiency up to 97.1%. Despite varying fucoidan structures across different types of algae^6^, the metabolic functions of degraders remained conserved, enabling quantitative prediction of degradation outcomes based on community and substrate composition. The frequent co-occurrence of functionally complementary fucoidan degraders in ocean metagenomes suggests that synergistic degradation is a globally relevant strategy. Our findings suggest that the environmental turnover of complex biopolymers depends not only on individual metabolic capabilities of degraders but also on ecological interactions shaped by substrate architecture. This work provides a mechanistic framework for understanding carbon cycling in the ocean and for engineering synthetic microbial consortia to degrade recalcitrant polysaccharides.

## Abstract (web summary)

Efficient degradation of fucoidans depends on complementary bacterial guilds that cooperatively break backbone and side-chain sugars, revealing a conserved, globally relevant mechanism that shapes marine carbon cycling.

## Main

Plant and algal polysaccharides are among the most abundant and diverse biopolymers on Earth^7,8^. Their degradation by microbial communities drives carbon cycling^9,10^, promotes gut health^11^ and enables sustainable biotechnologies^12,13^. As the main component of protective extracellular matrices in plants and algae, the chemical diversity of polysaccharides has escalated a co-evolutionary arms race, driving the diversification of carbohydrate-active enzymes and their reshuffling among microbial degraders via horizontal gene transfer^14–17^. Although these distributed metabolic capabilities are evident in many microbial ecosystems, it remains unclear how multiple degraders coexist on a single complex polysaccharide resource and engage in synergistic interactions rather than competition^18–23^. Consequently, we lack a quantitative, mechanistic framework linking the metabolism and interactions of individual degraders to degradation on a community level. This gap hinders our understanding of microbial contributions in carbon cycling and our ability to design microbial consortia for efficient degradation of diverse substrates.

In marine ecosystems, brown algae and diatoms produce the recalcitrant polysaccharide fucoidan, giving them a key role in carbon sequestration. They account for one-fifth of marine primary production and, through sinking biomass and particles, export 5 GtC yr^−1^ to the ocean depths, where the carbon can be stored for millennia^24–26^. These natural processes are increasingly harnessed in brown algal aquaculture, which is projected to contribute at least 0.5% of the 1 GtCO_2_ yr^−1^ sequestration target set for nature-based climate solutions by 2050^27^. Fucoidan is a major agent of algal carbon export, as it constitutes 25–50% of the cell wall and mucilage^6,28^, promotes particle formation, and resists microbial degradation for up to several months^1,29^. This stability is probably attributable to its complex structure, which comprises a sulfated fucose backbone that varies across algal species in linkage patterns and sulfation, as well as in the heterogeneous composition and linkage architecture of non-fucose side chains^6^. Because of this complexity, only a few bacterial species are known to degrade fucoidans, and those that do typically only achieve incomplete degradation despite encoding dozens of fucoidan-active enzymes^3–5^. Metagenomic studies reveal co-occurring degraders with complementary enzyme repertoires that potentially target different regions of the polysaccharide^30–32^, suggesting that complete degradation may depend on positive interactions among specialized microorganisms—an ecological hurdle that could stabilize fucoidan in a dilute environment such as the ocean and help explain why many algae rely on this polysaccharide as a protective layer.

## Enzymatic specialization among degraders

**Fig. 1:**
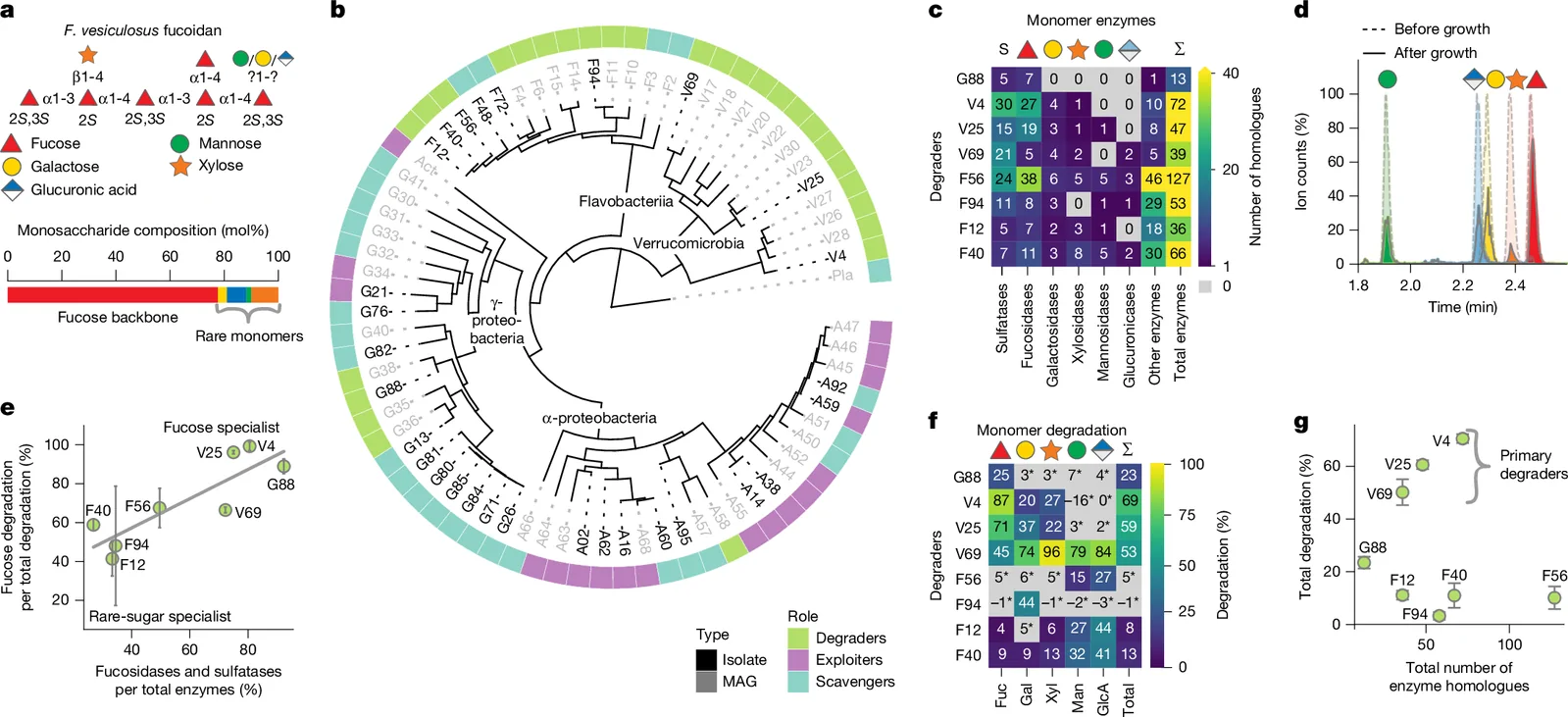
Fucoidan degraders in communities specialize through complementary monomer degradation. **a**, Schematic structure of the main repeating unit of fucoidan from *F. vesiculosus* and corresponding monosaccharide composition shown as bar graph. Monosaccharides are depicted following the Symbol Nomenclature for Glycans; glycosidic linkages are indicated as text and the question mark denotes unresolved linkages of mannose, galactose and glucuronic acid. **b**, Phylogenetic tree of marine bacterial strains enriched on fucoidan as sole carbon source. The outer ring indicates inferred metabolic roles; names are coloured to distinguish isolates from metagenome-assembled genomes. **c**, Number of fucoidan-associated enzymes across isolated degraders. Enzymes were grouped by inferred activities targeting similar fucoidan linkages: sulfatases (S1_15, S1_16, S1_17, S1_22 and S1_25), fucosidases (GH29, GH95, GH107, GH141 and GH168), galactosidases (GH36, GH97 and GH2), xylosidases (GH39, GH120, GH3, GH30 and GH31), mannosidases (GH92) and glucuronidases (GH115). ‘Other enzymes’ denotes fucoidan-associated CAZymes with unresolved activity. Σ indicates total fucoidan-associated enzymes per genome. Values indicate homologue counts, grey cells indicate zero counts and colour bar is capped at 40. **d**, Representative targeted LC–MS chromatograms of acid hydrolysed culture supernatants of V69 before (dotted) and after (solid) growth on fucoidan used to infer monomer degradation. Peaks are coloured by monomers identified by multiple reaction monitoring and retention time. For visualization, ion counts of monomers are normalized to before-growth samples. **e**, Correlation between proportion of enzymes targeting sulfate and fucose per total enzymes and the proportion of fucose degraded per total degradation. Dots and error bars show mean ± s.d. of three biological replicates. **f**, Heat map showing the mean monomer degradation obtained from three biological replicates; grey indicates no significant change from control. **g**, Number of fucoidan-degrading enzymes versus total fucoidan degradation across degraders. Dots and error bars show mean ± s.d. of three biological replicates. Source data

**Extended Data Fig. 1:**
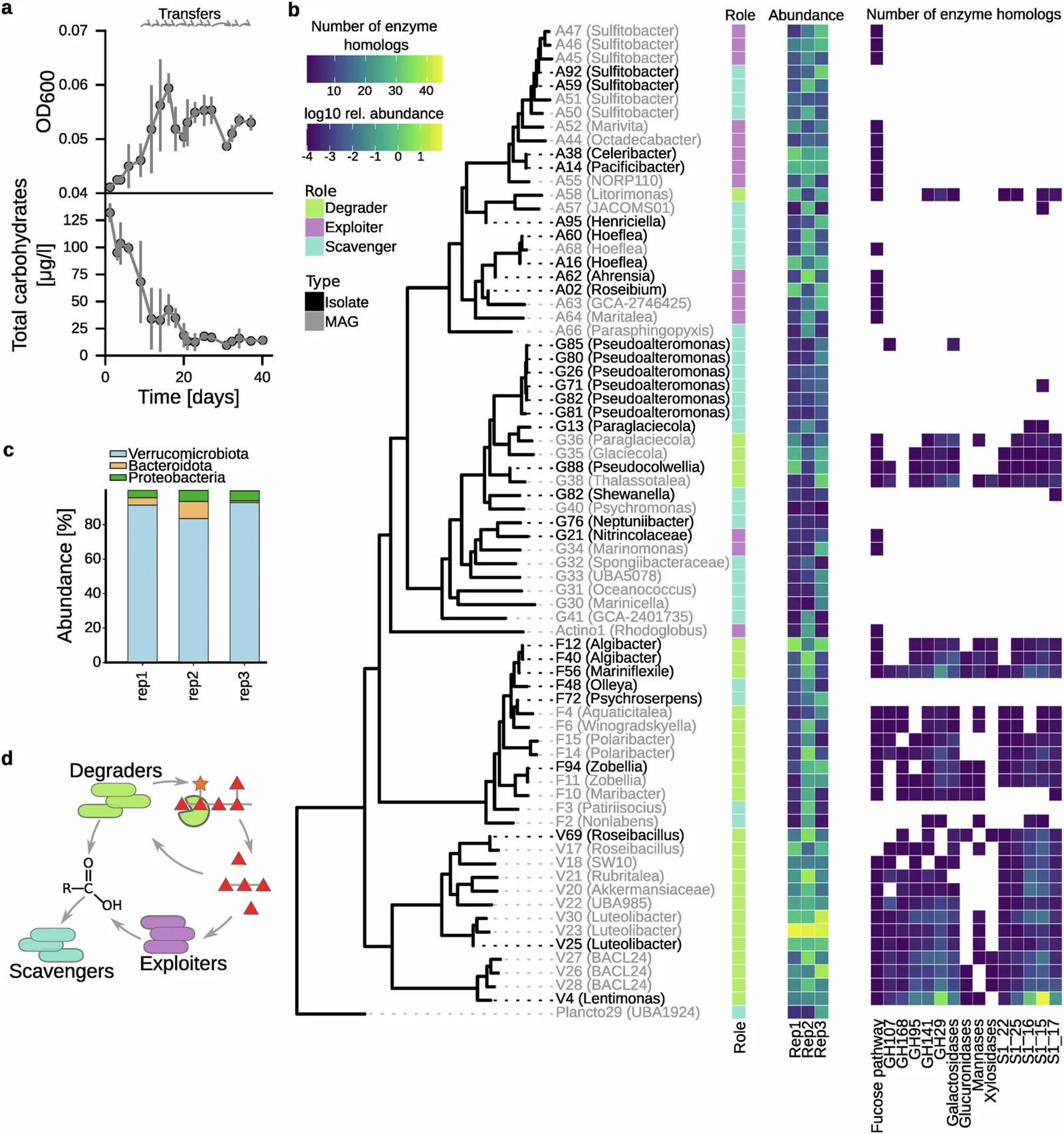
Genome centric analysis of fucoidan enrichments. **a**, Growth and total carbohydrate concentrations in three replicates of fucoidan enrichment cultures inoculated with the same seawater sample. Data points and error bars represent mean ± s.d. (n = 3), grey arrows indicate 1:50 transfers into fresh medium. **b**, Maximum-likelihood phylogeny of MAGs (grey) and isolates (black) reconstructed from the enrichments, based on a concatenated alignment of 120 single-copy marker genes. Accompanying heatmaps show (from left to right): (i) inferred metabolic roles; (ii) relative abundance at the final time point in all three replicates; (iii) presence of a complete fucose degradation pathway and counts of homologs in selected sulfatase and CAZyme families. Only medium and high-quality genome assemblies are included. **c**, Phylum-level composition at the final time point. **d**, Schematic of metabolic interactions in fucoidan-degrading communities. Degraders initiate fucoidan degradation via extracellular enzymes. Breakdown products are utilized by both degraders and exploiters, while scavengers rely on metabolic waste products released by degraders or exploiters.

To understand the role of microbial interactions in fucoidan degradation, we enriched a bacterial community from coastal seawater using fucoidan from the common brown alga *Fucus vesiculosus* as the sole carbon source (Supplementary Table 1). The previously characterized structure of this fucoidan (Fig. 1a) can be conceptualized as two compositionally and structurally distinct resource pools: a sulfated fucose backbone (about 80% of monomers) comprising an α-(1→3) or α-(1→4)-linked main chain with short α-(1→4)-linked fucose branches, and side chains composed of the ‘rare-sugar monomers’ xylose, galactose, mannose and glucuronic acid^6,33–35^ (Supplementary Note 1). After 12 sequential growth–dilution cycles, enrichments achieved 90% substrate degradation (Extended Data Fig. 1a–c). Metagenomic analysis recovered 73 metagenome-assembled genomes (MAGs), with Verrucomicrobiota—a phylum known for degraders of complex polysaccharides—dominating the community at over 80% relative abundance (Supplementary Table 2). Notably, the final points of all enrichments were dominated by *Luteolibacter*, a close relative of known fucoidan degraders that inhabit algal surfaces^32,36^. Based on gene content, MAGs were classified into three ecological guilds^37,38^: (1) ‘degraders’, which harbour fucosidases and sulfatases in their genomes and initiate polysaccharide breakdown; (2) ‘exploiters’, which lack these enzymes but possess genes for fucose catabolism, enabling them to compete with degraders for released fucose; and (3) ‘scavengers’, which do not contain either class of enzymes and instead rely on metabolic byproducts for growth (Fig. 1b and Extended Data Fig. 1d).

From these enrichments, we established a strain collection that reflects the taxonomic and functional diversity of the community (Fig. 1b). We isolated 5 exploiters, 16 scavengers and 7 degraders, namely V25 (*Luteolibacter*), V69 (*Roseibacillus*), G88 (*Pseudocolwellia*) and four members of the Flavobacteriia (F12, F40, F56 and F94). We additionally included the previously characterized degrader *Lentimonas* sp. CC4 as strain V4^3^. Isolate classification was experimentally validated by confirming that degraders grow on fucoidan, exploiters grow on fucose (but not fucoidan), and scavengers are unable to utilize either substrate (Supplementary Fig. 1). Although several degraders were not previously described, we found that in particular V69 and V4 co-occur across many macroalgae-associated coastal habitats (Supplementary Fig. 2 and Supplementary Note 2), highlighting the ecological relevance of this strain collection. Collectively, these 29 strains provide a foundation for dissecting the mechanisms of fucoidan degradation in microbial communities.

**Extended Data Fig. 2:**
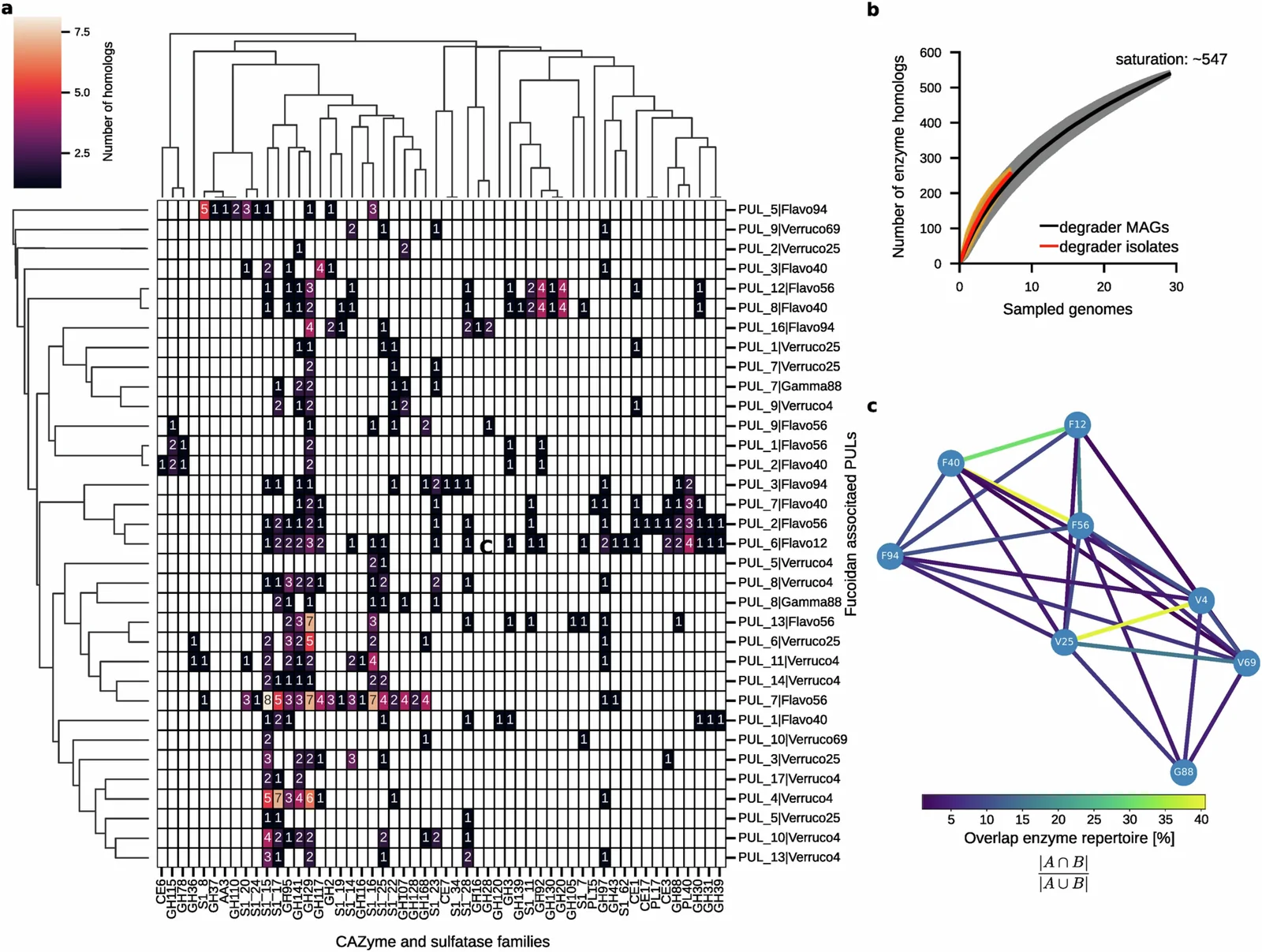
Comparison of enzyme repertoires in genomes of fucoidan degraders. **a**, Heatmap of homolog counts per enzyme family across polysaccharide utilization loci (PULs) associated with fucoidan degradation in isolated degraders. Each column corresponds to a PUL, each row to an enzyme family. Numbers denote the count of homologs per family per locus. Dendrograms reflect hierarchical clustering based on pairwise correlation. **b**, Rarefaction analysis of enzyme homologs identified in isolated degraders (orange) and in both isolates and MAGs (black). Solid lines indicate the average number of unique homologs per genome; shaded areas show s.d. from 10,000 random samplings. Saturation was estimated using the Chao1 estimator. **c**, The nodes of the network represent isolated degraders and the edges are coloured by the normalized similarity of their enzyme repertoires calculated from the Jaccard similarity.

**Extended Data Fig. 3:**
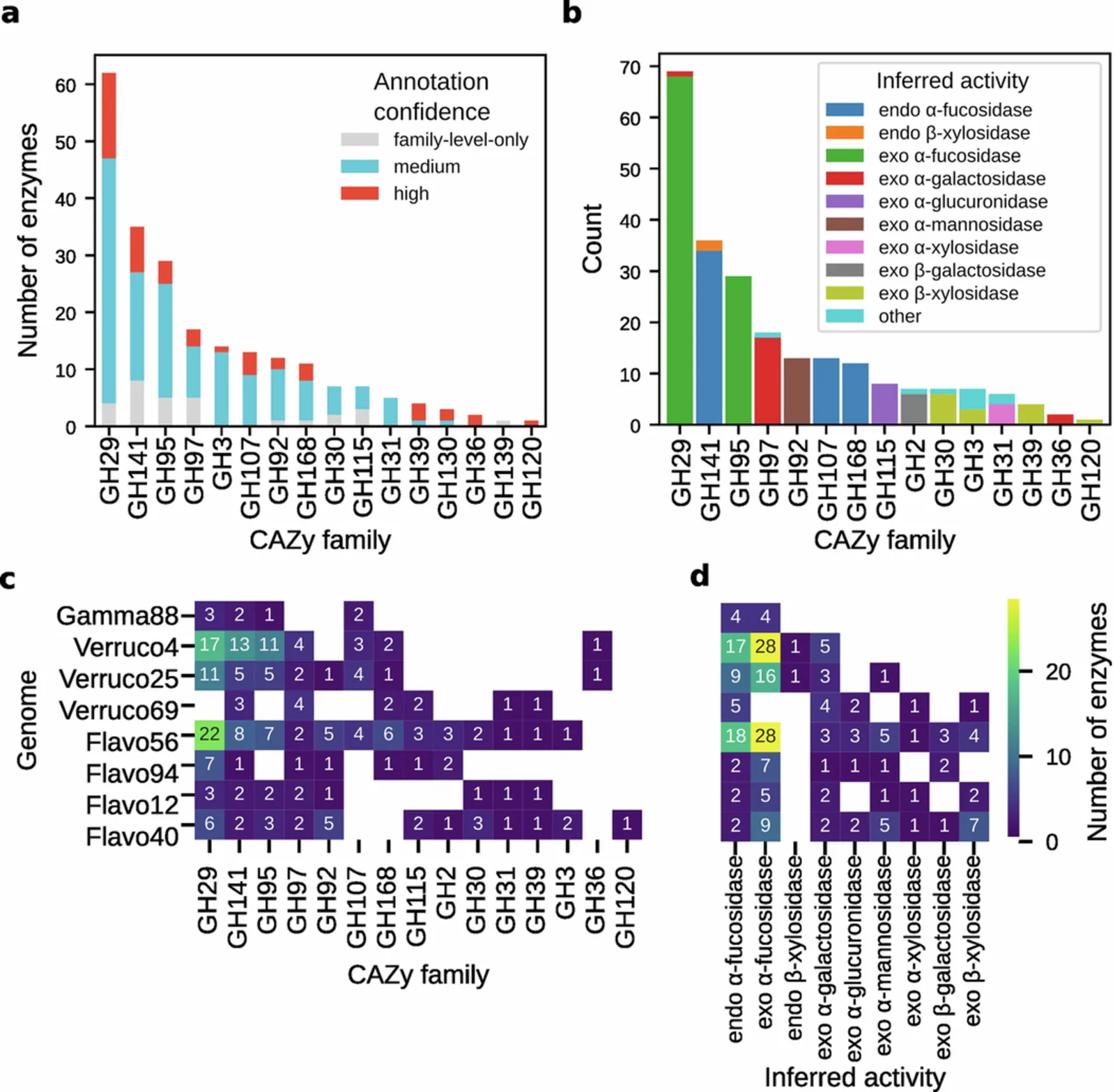
Annotation confidence and functional classification of fucoidan-associated enzymes. **a**, Annotation confidence of major CAZyme families implicated in fucoidan degradation and identified through co-localization in PULs across genomes of all eight isolated degraders. Annotations were classified based on sequence identity and alignment coverage into high-, medium- and family-level-only assignments. **b**, Inferred enzymatic activities per CAZy family. Bars show the number of enzymes assigned to each activity across all genomes. Inferred EC numbers are reported in Supplementary Table 3. Activities were assigned based on EC number, CAZyme family, closest characterized homolog and compatibility with known fucoidan linkage chemistry. **c**, Heatmap showing the number of enzymes per CAZy family and genome. **d**, Heatmap showing the number of enzymes per coarse-grained inferred activity class and genome.

Genome analysis of the isolated degraders revealed an unexpectedly large and diverse repertoire of fucoidan-degrading enzymes. We identified 34 genomic polysaccharide utilization loci (PULs) associated with fucoidan degradation, each encoding a different combination of enzymes (Extended Data Fig. 2 and Supplementary Table 3). Characterized enzyme families that target sulfate and fucose linkages^3,4,39–45^ co-localized with 44 additional enzyme families, including 10 families predicted to act on rare-sugar monomers (Extended Data Fig. 3 and Supplementary Note 3). The characteristic polysaccharide-binding and transport pair SusC/D was only found in Flavobacteriia (Supplementary Fig. 3). Individual degraders encoded 13–127 distinct enzymes, defined at a 60% amino acid identity threshold. Repertoires overlapped by only 9% on average between strains, yielding 547 unique enzymes across all enrichment-culture genomes (Extended Data Fig. 2b,c). Notably, all enzymes of strain G88 were encoded on a 112 kb plasmid, whereas strain F56 harboured a 623 kb genomic island containing five PULs (Supplementary Fig. 4). These observations are consistent with the lateral acquisition of large carbohydrate-active enzyme (CAZyme)-rich gene clusters^14,17^. Together, these findings indicate that enzyme repertoires are shaped by gene mobility and partial sampling from a vast environmental pool, culminating in a highly diverse enzymatic landscape for fucoidan degradation.

**Extended Data Fig. 4:**
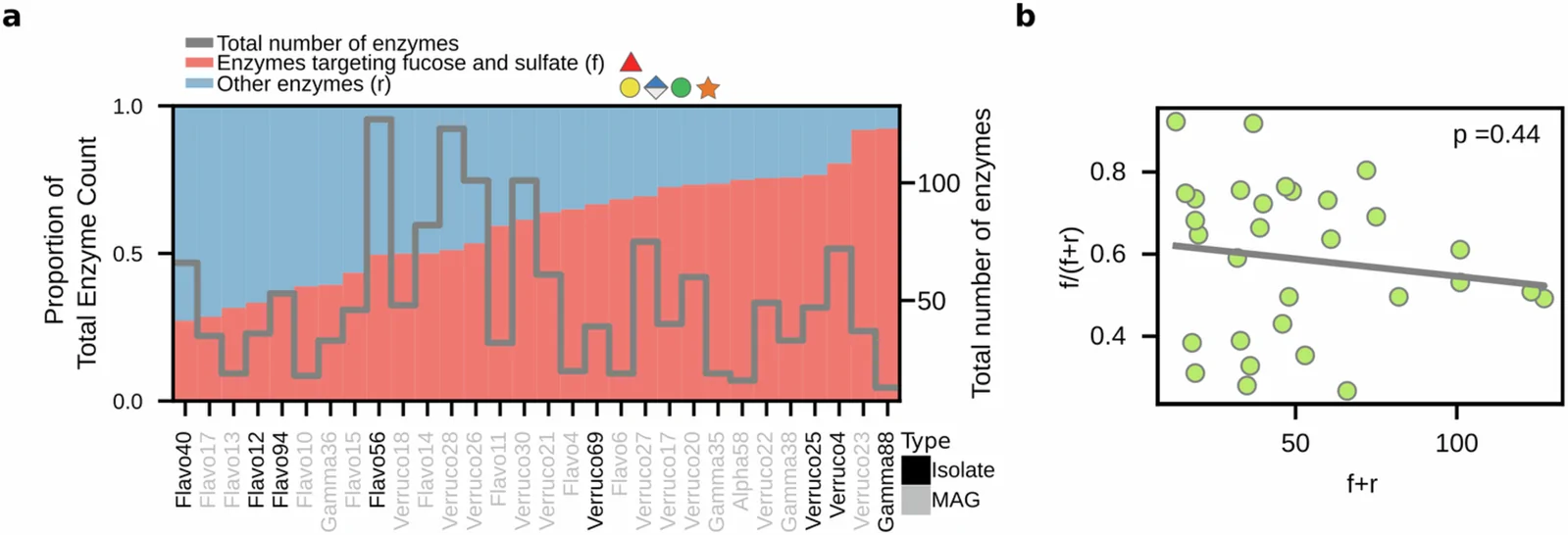
Enzymatic specialization of fucoidan degraders. **a**, Total number of fucoidan-associated enzymes and the proportion of enzymes assigned to sulfate- and fucose-targeting families, comprising sulfatases (S1_15, S1_16, S1_17, S1_22 and S1_25), endo-fucoidanases (GH107, GH141 and GH168) and exo-α-L-fucosidases (GH29 and GH95), or to other fucoidan-associated families, including enzymes predicted to act on rare-sugar monomers: galactosidases (GH36, GH97 and GH2), xylosidases (GH39, GH120, GH3, GH30 and GH31), mannosidases (GH92) and glucuronidases (GH115). **b**, Relationship between total number of fucoidan-targeting enzymes and the proportion of sulfated fucose targeting enzymes across 28 degrader genomes. Each point represents one genome; the grey line shows a linear regression (ordinary least squares) with corresponding P value.

Despite the unique enzyme repertoires found across genomes, a distinct functional differentiation emerged between fucose and rare-sugar degraders. Independent of the total enzyme count, the proportion of enzymes targeting sulfate and fucose linkages within the total fucoidan enzyme repertoires varied considerably—ranging from under 30% in F40 to over 90% in G88 (Extended Data Fig. 4)—indicating different levels of genomic specialization for the cleavage of fucose or other monomer linkages. Furthermore, the fucose specialists V25, V4 and G88 lacked glucuronidases and encoded few enzymes required for cleaving side chains of rare-sugar monomers (Fig. 1c). Conversely, the rare-sugar specialists F12, F40 and F94 contained fewer fucosidases and sulfatases. Strains F56 and V69 appeared to be generalists, possessing enzymes from both functional classes, although V69 lacked exo-acting fucosidases from families GH29 and GH95 (Extended Data Fig. 3c). These patterns suggest distinct preferences for fucoidan monomers across degraders.

## Complementary monomer degradation

**Extended Data Fig. 5:**
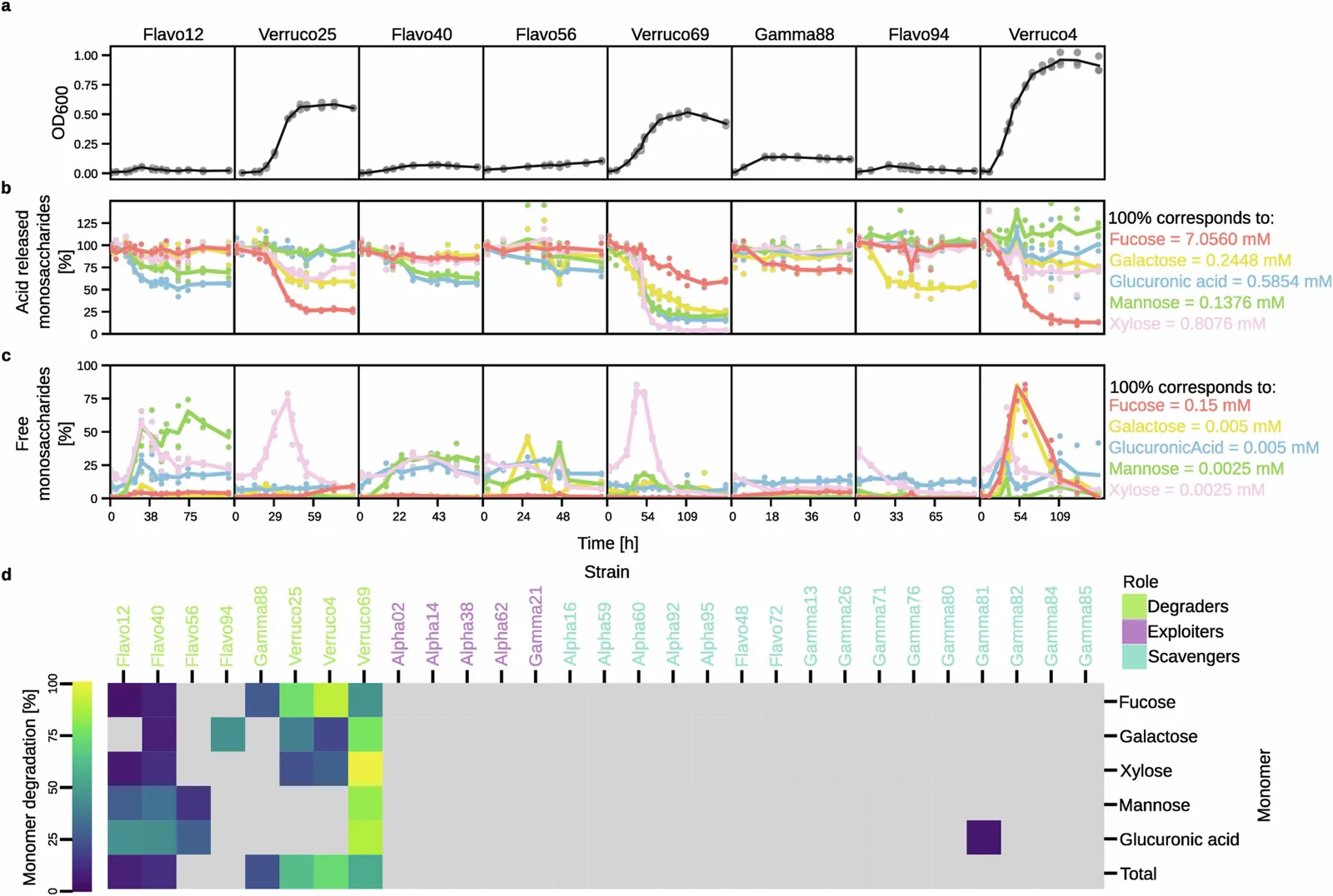
Characterization of fucoidan degradation at the monomer level. **a**, Growth of eight degraders on fucoidan from *Fucus vesiculosus* as the sole carbon source. Grey points represent individual measurements and black lines indicate the mean of three biological replicates (n = 3). **b**, Time-resolved changes in fucoidan-derived monosaccharides during growth. Following acid hydrolysis of culture supernatants, absolute concentrations of fucose, galactose, glucuronic acid, mannose and xylose were quantified by targeted LC–MS. Points represent individual measurements and lines indicate means of three biological replicates (n = 3). For visualization, concentrations are normalized to initial levels; absolute concentrations each monosaccharide are indicated on the right. **c**, Concentrations of free monosaccharides in the extracellular medium. Monosaccharides released during growth were directly quantified by targeted LC–MS. Points represent individual measurements and lines indicate means of three biological replicates (n = 3). Concentrations are normalized to initial levels for visualization; absolute concentrations of each monosaccharide are indicated on the right. **d**, End-point measurements of fucoidan degradation across all 29 strains. The heatmap shows the average decrease in fucoidan-bound monomers from three independent growth experiments (n = 3) after acid hydrolysis relative to initial concentrations. Grey cells indicate changes that are not significantly different from the no-cell control.

We validated our genomic predictions of enzymatic specialization using a targeted liquid chromatography mass spectrometry (LC–MS) assay to analyse fucoidan degradation at the monomer level. Monomers bound in fucoidan were released through acid hydrolysis of culture supernatants and quantified using established derivatization protocols and an optimized 3.5 min LC–MS method^46,47^. Time-resolved measurements across all eight degraders showed that monomer depletion closely aligns with growth, saturating once cultures entered stationary phase (Extended Data Fig. 5a–c and Supplementary Table 4). During exponential growth, we also observed a transient accumulation of free monosaccharides, most prominently fucose, which reached up to 150 µM in V4, as reported previously^3^. This suggests that extracellular fucoidan hydrolysis releases monosaccharides at rates that temporarily exceed their uptake rate. Subsequently, we quantified fucoidan degradation as the change in fucoidan-bound monomers before and after growth (Fig. 1d and equation (1)). This approach effectively captures the combined effect of enzymatic cleavage of the polysaccharide and microbial uptake of the released monomers—a process that we refer to as monomer degradation. The results confirmed that only the degraders contribute substantially to fucoidan turnover (Extended Data Fig. 5d). To assess metabolic specialization, we determined the monomer preference for each degrader as the fraction of consumed carbon derived from fucose. This metric strongly correlated with the genomic proportion of enzymes targeting sulfate and fucose linkages relative to the total fucoidan repertoire (*r* = 0.75, *P* < 0.001; Fig. 1e). This confirms that V69 and F56 function as generalists, whereas V4, V25 and G88 are fucose-specialists and F12, F40 and F94 are rare-sugar specialists.

None of the degraders were able to completely break down any specific monomer type. The fucose specialists V4 and V25 achieved only 87% and 71% degradation of fucose, respectively (Fig. 1f), but exhibited limited activity on galactose and xylose, and did not degrade mannose or glucuronic acid. By contrast, mannose or glucuronic acid were effectively consumed by F12, F40, F56 and V69, but exhibited low levels of fucose degradation. This metabolic specialization resulted in limited total degradation, even among the strongest degraders V4, V25 and V69 that achieved 69%, 59% and 53%, respectively—substantially lower than the ~90% degradation observed in enrichment cultures. Notably, a larger enzymatic toolkit does not necessarily mitigate this bottleneck (Fig. 1g). For example, F56, despite encoding >100 enzymes, achieves negligible total degradation, whereas G88 reaches 23% with only 13 enzymes. This decoupling indicates that access to complementary substrate fractions—rather than enzyme count—limits fucoidan degradation, such that complete breakdown emerges from interactions among metabolically complementary degraders.

## Synergism in pairwise co-cultures

**Fig. 2:**
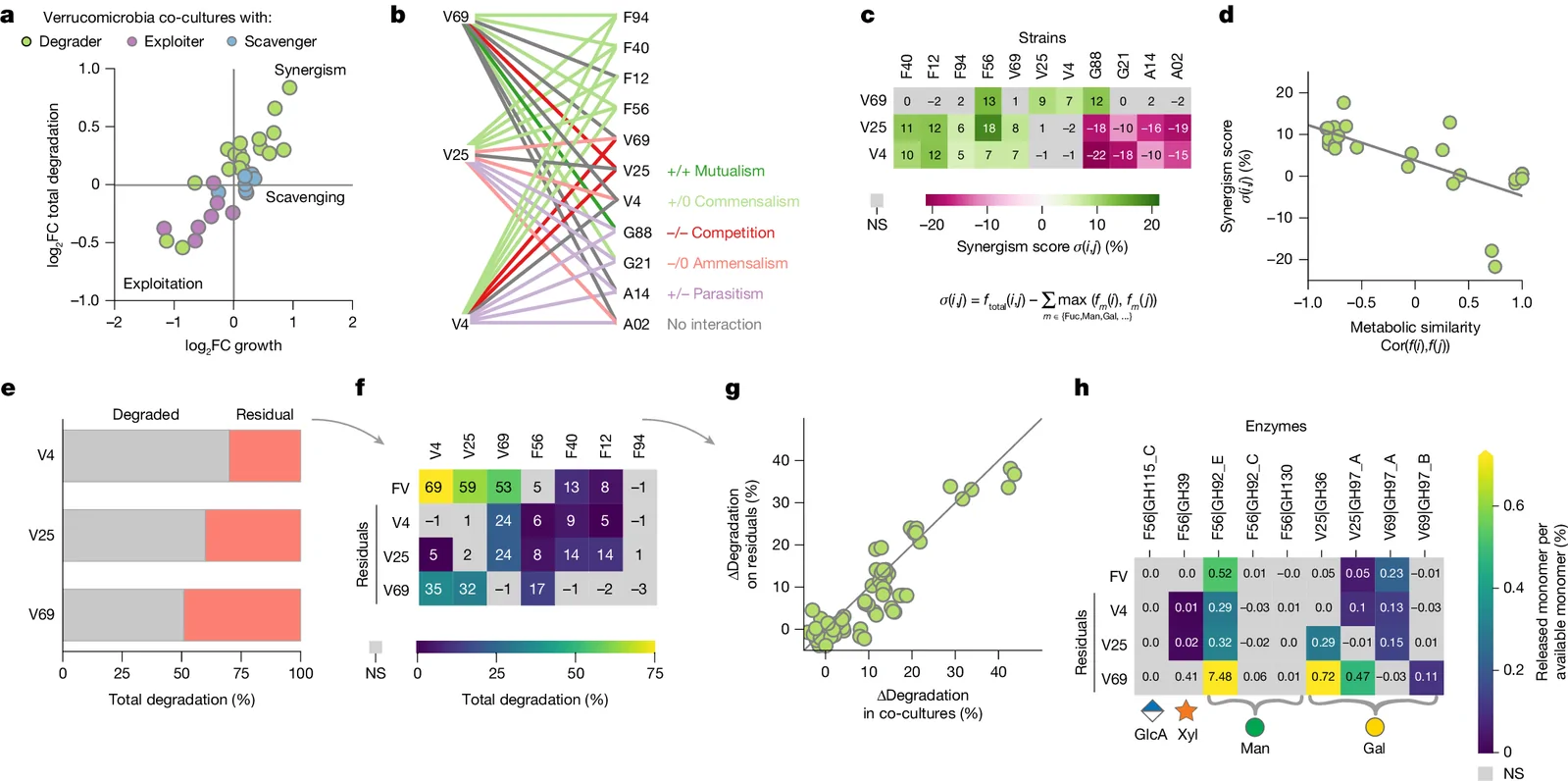
Synergistic interactions between complementary degraders in pairwise co-cultures. **a**, log_2_-transformed fold changes (FC) in growth yield and total fucoidan degradation in 87 pairwise co-cultures of Verrucomicrobiota strains with other community members, relative to the corresponding Verrucomicrobiota monoculture. Only significant changes are shown; dots are colour-coded according to the metabolic role of the partner strain. **b**, Network of pairwise interactions inferred from changes in strain abundance in co-culture relative to monoculture, measured by strain-specific qPCR. Nodes denote strains and edges interaction types. **c**, Synergism score (*σ*(*i*,*j*)) for Verrucomicrobiota strains paired with other community members. Scores quantify deviations from a null model of independent substrate utilization. Grey indicates no significant (NS) change from null expectation. **d**, Relationship between synergism score and metabolic similarity of strain pairs, calculated as the pairwise correlation between *z*-score normalized monomer degradation profiles in monoculture. **e**, Fucoidan degradation by three primary degraders and the corresponding residual substrate used in cross-feeding assays. **f**, Additional degradation of primary residual fucoidans by secondary degraders, scaled to the initial fucoidan pool (residual fraction × fraction degraded by secondary degrader). Grey indicates no significant (NS) change compared to controls. Data in **a**–**f** represent means of 3 biological replicates (*n* = 3); replicate-level data and exact *P* values are provided in the Source Data file. **g**, Relationship between additional degradation in co-cultures and degradation of purified residual fucoidans. Co-culture values were calculated as total degradation in the co-culture minus degradation by the primary degrader alone. Residual values indicate degradation achieved by the secondary degrader on purified residual fucoidan. Dots show individual biological replicates. **h**, Release of monosaccharides from fucoidan and fucoidan-derived residual substrates by purified enzymes, normalized to the total amount of the respective monosaccharide present in the substrate. Monosaccharide symbols and text indicate the type of released monosaccharide for each enzyme. Values represent the mean of three biological replicates (*n* = 3) and grey indicates no significant (NS) release. Source data

**Extended Data Fig. 6:**
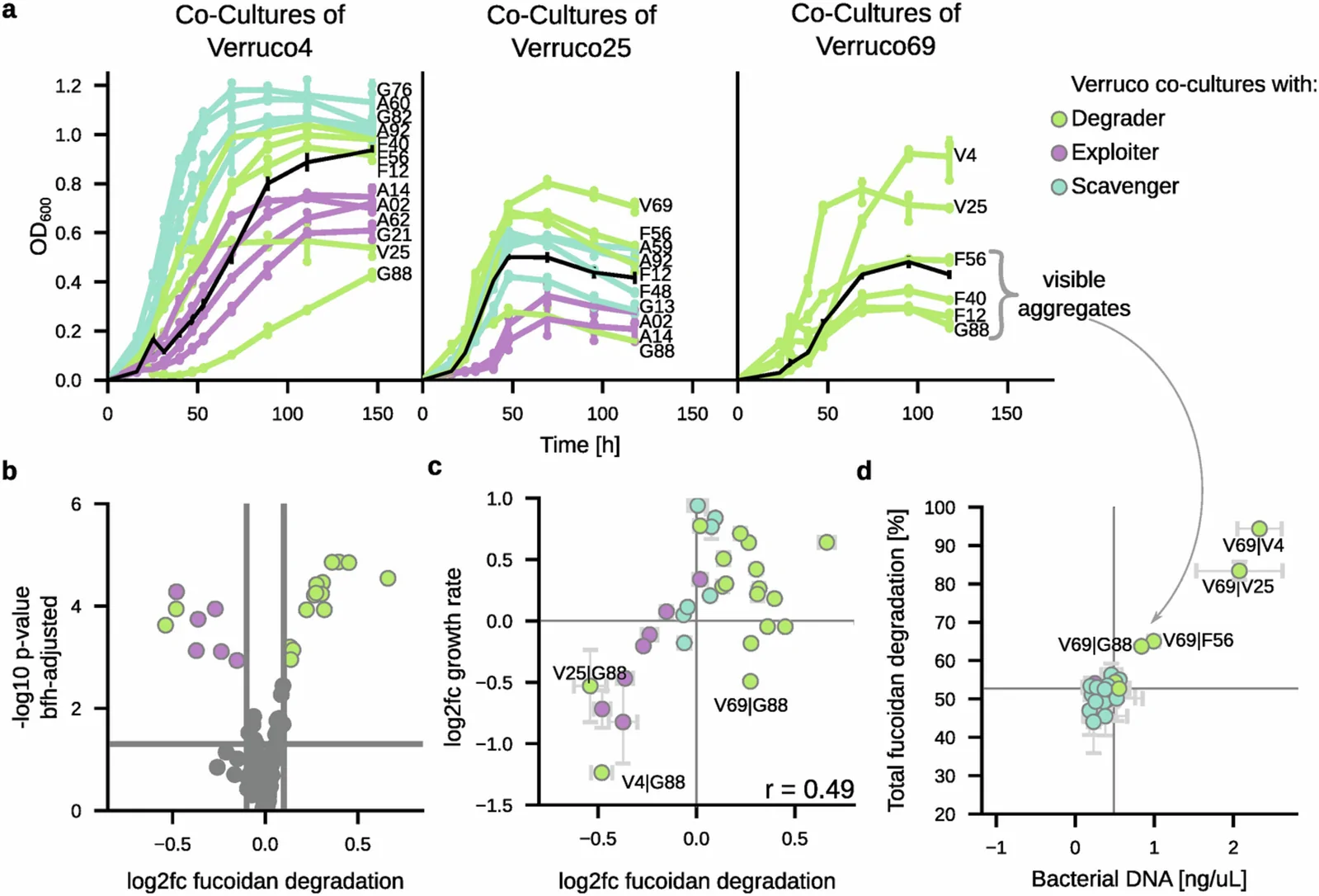
Analysis of growth and fucoidan degradation in Verrucomicrobia-centric co-cultures. **a**, Growth curves of Verrucomicrobia primary degraders (black lines) in pairwise co-culture with partners of different functional roles. Only co-cultures showing significant changes in maximal OD_600_ are displayed; interaction partners are labelled and colour-coded by functional role. Solid lines and errorbars represent mean ± s.d. of three independent growth experiments (n = 3). **b**, Volcano plot showing *P*-values of log_2_-fold changes in fucoidan degradation in co-cultures compared to corresponding monocultures calculated based on independent growth experiments (n = 3). Statistical significance was assessed using two-sided unpaired t-tests, followed by Benjamini–Hochberg correction for multiple comparisons. Coloured points indicate significant changes, grouped by the role of the interaction partner. **c**, Relationship between changes in fucoidan degradation and growth rate across all co-cultures. Only significant differences are shown. **d**, Comparison of total bacterial biomass (measured by qPCR targeting bacterial DNA) and fucoidan degradation across all co-cultures of Verruco69. The grey arrow highlights four co-cultures (with G88, F56, F12 and F40) that formed visible precipitates, which interfered with optical density (OD_600_) measurements shown in panel **a**. For these four co-cultures, OD-based estimates of changes in biomass were substituted by DNA-based estimates, including those shown in Fig. 2a. All data points and error bars represent mean ± s.d. of three biological replicates (n = 3).

To assess the impact of microbial interactions on fucoidan degradation, we measured the fold changes in growth yields and total degradation for the primary degraders V4, V25 and V69 when co-cultured individually with each of the 29 strains, comparing the results to monoculture controls (Fig. 2a, Extended Data Fig. 6 and Supplementary Table 5). Of the 87 co-culture pairs tested, 33 exhibited significant changes that broadly aligned with predictions based on genome-based guild classifications: Eight pairs with scavengers increased biomass without influencing degradation, supporting their role in recycling byproducts that are not directly linked to the degradation pathway. Conversely, seven pairs with predicted exploiters reduced both growth and fucoidan degradation. Surprisingly, 16 degrader–degrader co-cultures showed positive interactions, characterized by increases in both biomass and degradation, as well as higher growth rates (Extended Data Fig. 6c). The only exception was G88, which increased degradation when paired with V69, but acted as an exploiter when paired with V4 and V25. On the subset of co-cultures with altered degradation outcomes, we classified interaction types using absolute species abundances determined by strain-specific quantitative PCR (qPCR)^48^ (Fig. 2b and Supplementary Table 6). Exploiters increased in biomass at the expense of primary degraders, consistent with opportunistic consumption of released fucose. Degrader–degrader interactions with increased total degradation and biomass were predominantly commensal, but also included cases of amensalism and competition; only the V69–G88 pair was mutualistic. These results distinguish ecological interaction types at the species level from positive interactions at the functional level, showing that reciprocal benefits to both strains are not necessarily required for enhanced community function.

The observed positive interactions among degraders could not be explained solely by resource partitioning, indicating the emergence of a synergism on a functional level. To quantify this, we defined a synergism score, denoted as *σ*(*i*,*j*), by calculating the difference between observed total degradation and a null hypothesis that assumed no interaction among strains beyond mere resource partitioning (Fig. 2c and equation (2)). The strongest synergism of 18% occurred between V25 and F56, which together achieved 79% total degradation. The highest degradation levels overall were found in the pairs V25/V69 (82%) and V4/V69 (93%), each exhibiting synergistic contributions of about 8%. Overall, a strong negative correlation was discovered between the calculated synergism *σ*(*i*,*j*) and the dissimilarity of monomer degradation profiles between paired strains (*r* = – 0.79, *P* < 0.001; Fig. 2d), indicating that complementary metabolic capabilities amplified degradation in a non-additive manner. Additionally, the relationship between synergism and complementarity was reflected in the genomes of degraders, as we identified a positive association between *σ*(*i*,*j*) and the difference in the genomic proportion of sulfate- and fucose-targeting enzymes relative to total enzymes (*r* = 0.53, *P* = 0.02; Supplementary Fig. 5). Collectively, these results indicate that synergism emerges through the combination of fucose and rare-sugar specialists when enzymatic and metabolic capabilities are maximized.

## Mechanism of synergism

To determine whether interactions among degraders were purely resource-mediated or involved additional metabolic exchanges, we measured degradation using cell-free, purified residual fucoidans (larger than 1 kDa) obtained from three stationary-phase Verrucomicrobiota cultures (Fig. 2e). These residual fucoidans contain carbon that is enzymatically inaccessible to the producing strain, even when supplied as a fresh carbon source (Fig. 2f). When offered to other degraders as the sole carbon source, LC–MS measurements showed that all degraders except F94 utilized at least one of the three residual substrates, and F56 exhibited a threefold increase in degradation of residuals compared to untreated fucoidan. This additional degradation quantitatively matched the enhanced performance observed in the corresponding co-cultures (*R*^2^ = 0.90, *P* < 0.001; Fig. 2g and equation (3)), indicating that synergism among degraders does not require direct cell–cell contact or cross-feeding of small molecules, but instead arises from complementary enzymatic activities that unlock otherwise inaccessible carbon.

**Extended Data Fig. 7:**
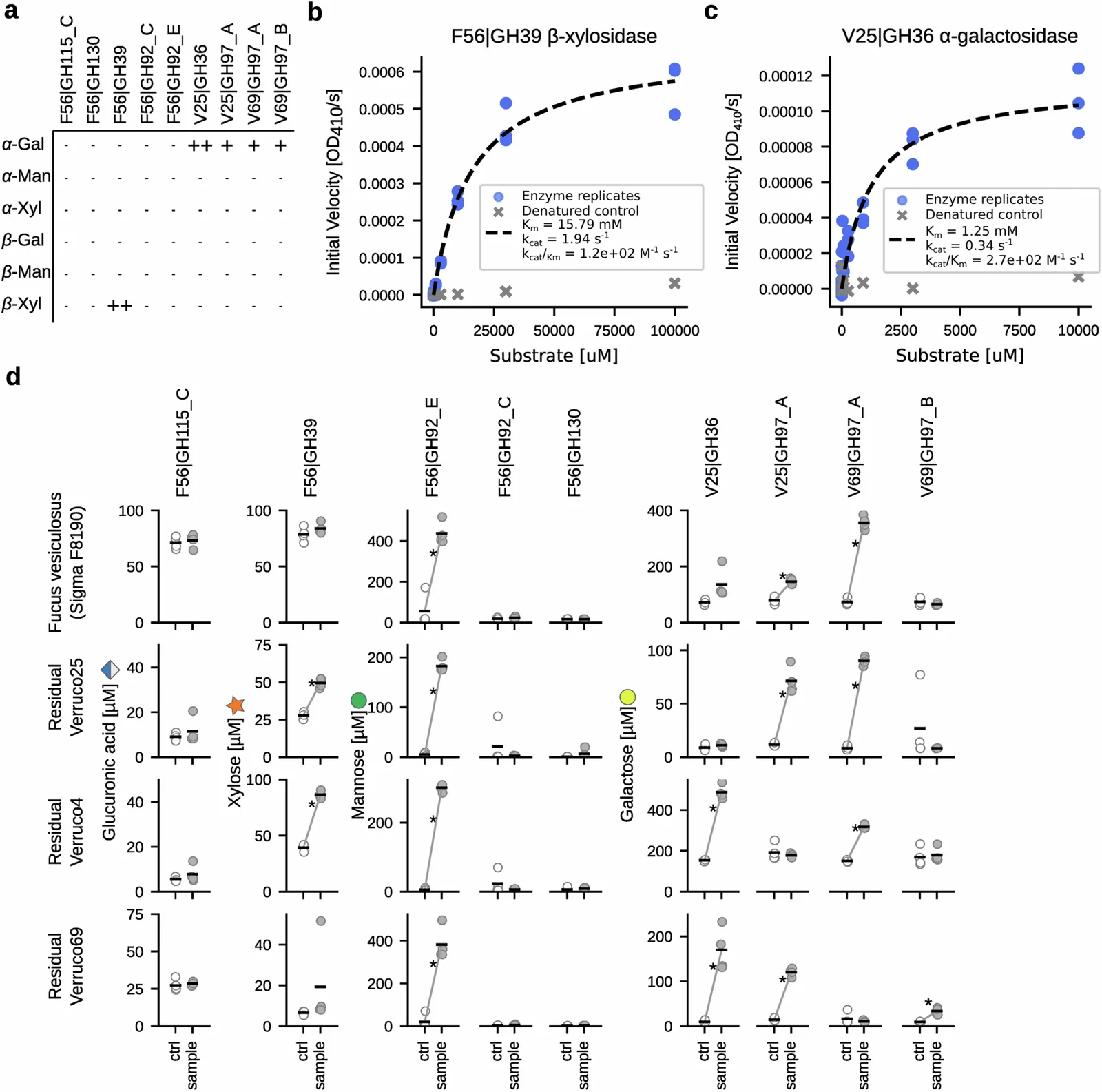
Functional characterization of glycoside hydrolases acting on rare-sugar monomers. **a**, Activity of heterologously expressed enzymes on pNP-labelled substrate analogues. Substrates are abbreviated by sugar identity and linkage, for example pNP-α-D-galactopyranoside is denoted α-Gal-pNP; all other substrates are abbreviated analogously (*e.g*. α-Man-pNP, β-Xyl-pNP). Activity measured in three independent enzyme assays (n = 3) was classified as follows: −, no activity detected after 24 h; +, increase in OD_410_ of 0.05–0.1 after 24 h; ++, increase in OD_410_ > 0.1 within 10 min. **b,c**, Enzyme kinetics of representative enzymes F56|GH39 (b, β-xylosidase activity on pNP-β*-*D-xylopyranoside) and V25|GH36 (c, α-galactosidase activity on pNP-α-D-galactopyranoside). Reaction velocities were measured across a range of substrate concentrations and fitted using Michaelis–Menten kinetics by nonlinear least-squares regression. Data points (blue) represent three individual measurements (n = 3); dashed lines indicate model fits. Denatured enzyme controls (grey) show no detectable activity. **d**, Release of monosaccharides from native fucoidan and fucoidan-derived residual fractions generated by primary degraders. Data points represent four independent enzyme assays (n = 4). Grey lines and asterisks indicate statistically significant release evaluated using one-sided paired Student’s t-tests with Benjamini–Hochberg correction for multiple comparisons with exact *P*-values reported in the Source Data file for Fig. 2h.

To test whether synergism arises from enzymatic complementarity, we purified and characterized nine predicted exo-acting enzymes targeting rare-sugar monomers. These enzymes, assigned to CAZyme families GH39, GH36, GH130, GH115, GH92 and GH97, were selected because of their uncharacterized roles in fucoidan degradation and their unique distribution among degraders (Extended Data Fig. 3, Supplementary Table 7 and Supplementary Figs. 6 and 7). Assays with pNP-labelled substrate analogues, native *F. vesiculosus* fucoidan and residual fucoidans derived from three Verrucomicrobiota degraders confirmed the expected substrate specificities for six enzymes, including α-galactosidases (V25|GH36, V25|GH97_A, V69|GH97_A, V69|GH97_B), an α-mannosidase (F56|GH92_E) and a β-xylosidase (F56|GH39), whereas three showed no detectable activity (Extended Data Fig. 7). Kinetic analyses yielded Michaelis constant (*K*_m_) values of 15.79 mM for F56|GH39 and 1.25 mM for V25|GH36, consistent with reported values^49,50^. Notably, several active enzymes are encoded in distinct fucoidan PULs, providing functional support for these loci (Supplementary Fig. 8). Monomer release from fucoidan substrates was low (0.01–7.48% of the initial pool), indicating that only a limited fraction of linkages is accessible to individual enzymes (Fig. 2h). By contrast, most enzymes exhibited substantially higher activity on residual fucoidan, indicating that prior enzymatic processing exposes otherwise inaccessible linkages, with up to tenfold increases for F56|GH92_E and V25|GH97_A. Additionally, the distinct activity profiles among GH97 homologues further indicate substrate partitioning across structurally heterogeneous side chains. Together, these results show that complementary hydrolase repertoires expand substrate accessibility and drive synergistic degradation in co-culture.

## Quantitative prediction of synergism

**Fig. 3:**
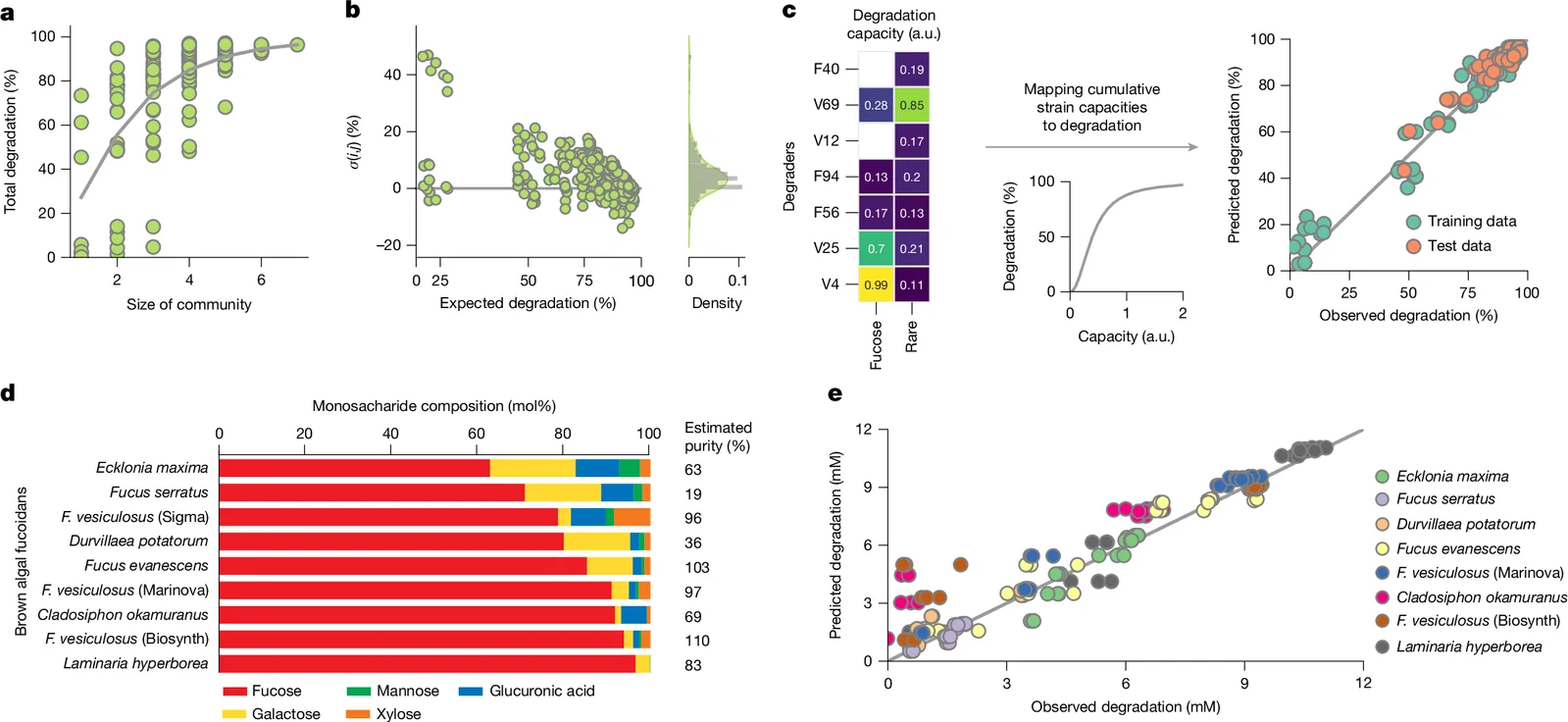
Functional complementarity enables predictable degradation synergies across different fucoidan structures. **a**, Total fucoidan degradation in all 127 possible combinations of up to 7 degraders. Data points show the mean of three biological replicates; the grey line denotes the mean degradation per community size. **b**, Synergism score in 127 communities compared to expected degradation under a null model assuming no interactions. Left, scatter plot of expected degradation versus synergism score. Right: density plot of synergism score distribution. All individual data points from three biological replicates are shown. **c**, Predictive model of community degradation based on strain-specific substrate preferences. Left, heat map of inferred degradation capacities for fucose and rare-sugar monomers across seven strains. Middle, nonlinear Hill function (*n* = 2.1, *K*_m_ = 0.4) mapping cumulative strain capacity to predicted degradation. Right, predicted versus observed degradation across 127 communities; dots are coloured by training or test set and show the mean of three biological replicates. a.u., arbitrary units. **d**, Monosaccharide composition and estimated fucoidan purity of nine brown algal fucoidans. Bars show the relative abundance of fucoidan-derived monosaccharides. The estimated fucoidan content provides an approximate measure of the fraction of fucoidan-derived material within the total hydrolysable carbohydrate pool. **e**, Observed versus predicted degradation of nine fucoidan substrates across seven selected communities. Dots are coloured by substrate identity and all data points from three biological replicates are shown. Source data

**Extended Data Fig. 8:**
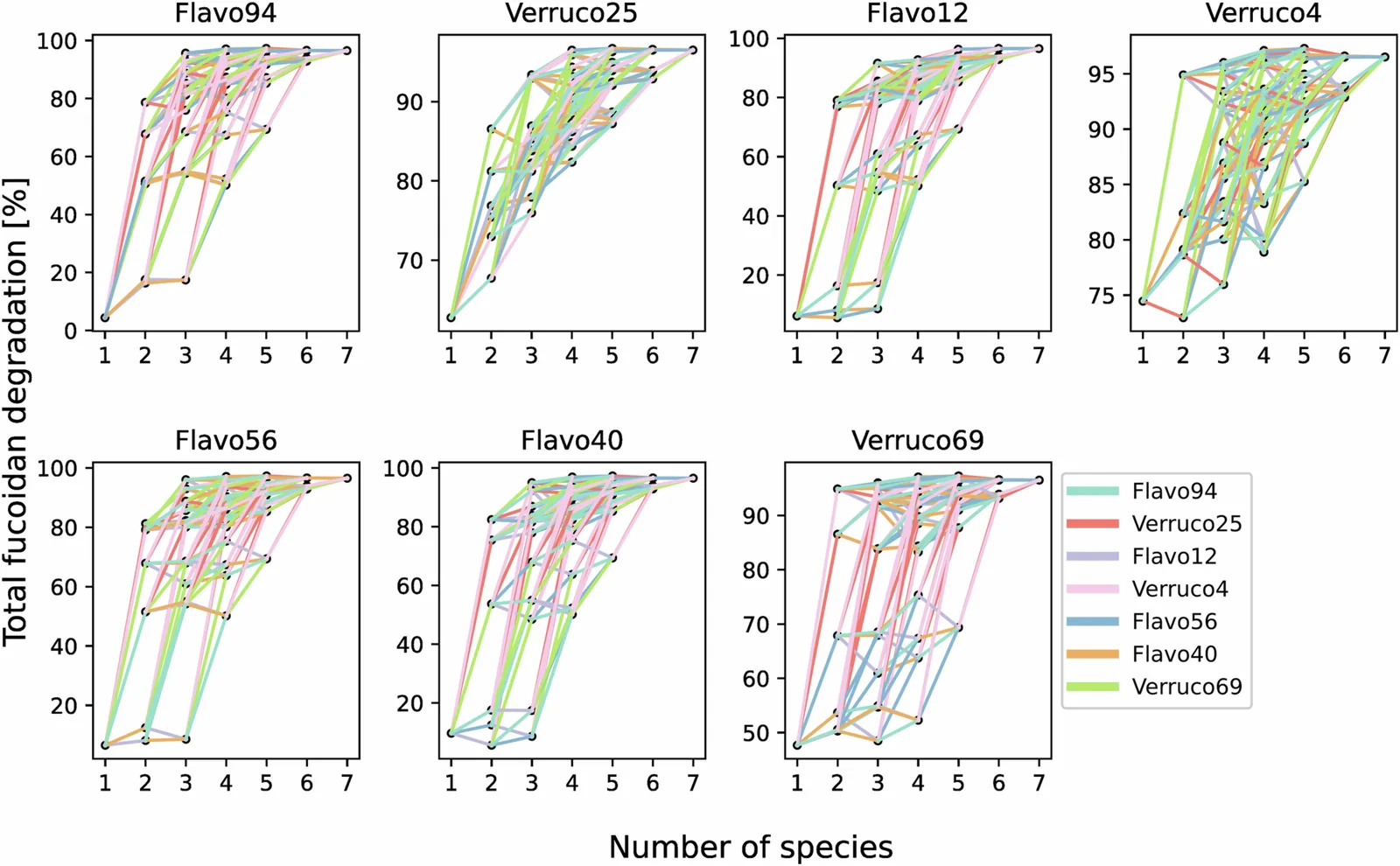
Fucoidan degradation in seven member communities. Composition–degradation landscape of the seven-member bacterial community, reconstructed from total fucoidan degradation measured across all possible combinations of one to seven degraders. Each node represents community-level fucoidan degradation (vertical axis) as a function of community size (horizontal axis). Edges connect communities that differ by the addition of a single degrader, with edge colour indicating the identity of the added strain; edge slopes therefore represent the change in degradation caused by adding a species in a given community context. Each subplot depicts the functional landscape originating from a different focal strain (monoculture at x = 1). Nodes represent mean total fucoidan degradation across three biological replicates (n = 3).

To explore whether synergistic effects extend beyond pairs, we measured degradation across all 127 combinations of the seven consistent degraders, excluding G88. Total degradation increased with community richness, ranging from 4% to a maximum of 97.1% in the 5-member community F40, F56, V69, F94 and V4 (Fig. 3a). A notable example of emergent synergism was observed between F56 and F94, which showed minimal degradation in monoculture but reached 50% degradation when combined (Extended Data Fig. 8).

Our results revealed a high degree of functional redundancy, with near-complete degradation achieved across multiple community configurations. Some configurations depended on a few high-performing strains (for example, V69 paired with V25), whereas others relied on synergistic combinations of individually weaker degraders (Supplementary Fig. 9). For example, four Flavobacteriia strains, although ineffective alone, collectively approximated the degradation performance of V69. This functional redundancy implies that diverse communities can serve as a buffer against the stochastic variation in species composition typical of particle-associated marine microbiomes^51,52^. Remarkably, even when approaching near-complete degradation, 80% of interactions were still synergistic beyond what would be expected from simple resource partitioning, while negative interactions were infrequent and weak (equation (2) and Fig. 3b). These findings indicate a smooth structure–function landscape with minimal higher-order effects and a high degree of predictability in community function^53^.

**Extended Data Fig. 9:**
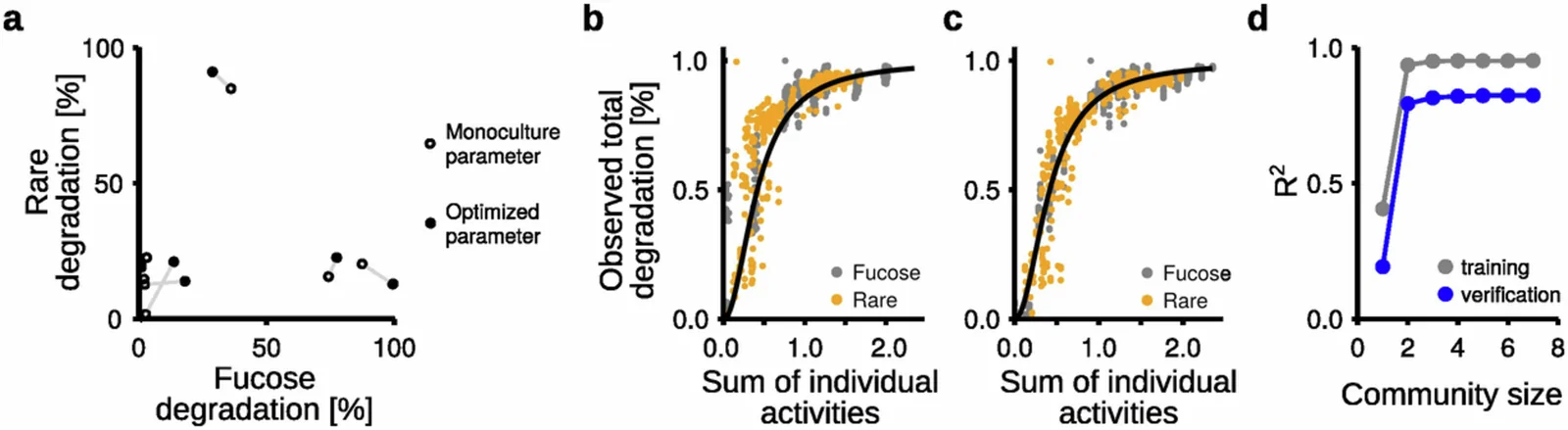
Trait-based modelling of community degradation. **a**, Effect of parameter optimization on the monomer degradation of seven degraders. Circles represent strain-specific degradation capacities before (hollow) and after (filled) optimization. Observed values were averaged from three biological replicates per strain. **b**, Model prediction using original monoculture-derived activities for each monomer pool. The black line shows a fitted Hill function. Grey and orange data points represent fucose and rare-sugar monomer degradation, respectively, measured across 127 communities in biological triplicates. **c**, Same as (**b**), but using optimized strain activity parameters. **d**, Model performance (R²) as a function of maximal community size included in training.

To further assess the predictability of degradation in complex communities, we developed a mechanistic model that links community composition to degradation outcome (Fig. 3c). This model was inspired by the observed resource partitioning between fucose and rare-sugar specialists and represents fucoidan as two monomer pools (fucose and rare) in which strains are characterized by their ability to degrade each type (equation (4)). Given that degradation tended to saturate in richer communities, we hypothesized that community-level degradation could be predicted by a nonlinear combination of the capabilities of individual strains, captured through a Hill function (equations (5) and (6)). Model parameters—strain-specific degradation potentials and Hill coefficients—were inferred from data on one-, two- and three-member communities (Extended Data Fig. 9). This straightforward framework proved to be highly effective, accurately predicting degradation across all 127 community combinations (*R*^2^ = 0.96, *P* < 0.001; Fig. 3c and Supplementary Table 8), including the emergent interaction between F56 and other degraders. These results highlight that, despite the structural complexity of fucoidan and the diversity of enzymatic pathways involved, community-level degradation can be reliably predicted from the synergistic contributions of individual strains to fucose and rare-sugar monomer utilization.

**Extended Data Fig. 10:**
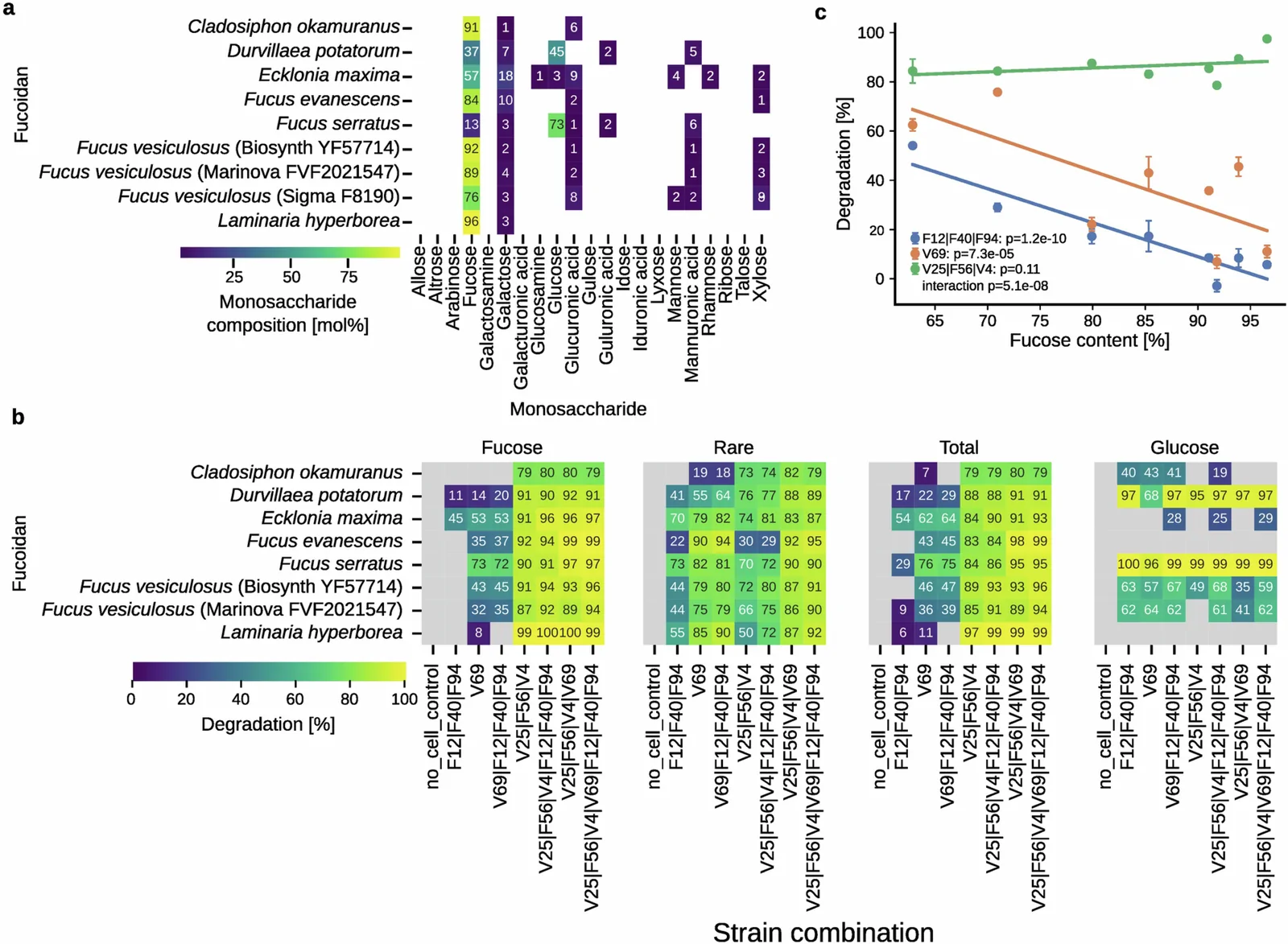
Degradation of compositionally diverse fucoidans. **a**, Monosaccharide composition of the fucoidans used in this study. Only monosaccharides with >1% abundance are shown and values represent means of three independent measurements (n = 3). **b**, Heatmaps showing degradation profiles for fucose, rare fucoidan monomers (galactose, xylose, glucuronic acid, mannose), total fucoidan monomers (fucose and rare monomer) and glucose across seven defined communities and eight fucoidan substrates. Values represent the mean of three biological replicates (n = 3). **c**, Community degradation of three selected communities as a function of fucoidan fucose content of eight tested fucoidans. Points show mean ± s.d. from three biologically independent cultures for each fucoidan–community combination (n = 3); lines show ordinary least-squares regressions fitted to the replicate-level data. The *P*-values of individual slopes tested against zero using two-sided t-tests on the regression coefficients are displayed as text labels Differences among slopes were assessed using a type II ANOVA F-test for the community × fucose interaction (F_2,66_ = 21.90, P = 5.1 × 10^−8^).

Given that the monomer composition, structure and degradation pathways of fucoidan vary substantially between brown algal species^54^, we validated the generality of our results across eight different types of fucoidan (Supplementary Note 4). We quantified degradation using full monosaccharide analysis to resolve co-extracted polysaccharides and degradation across seven communities. Most substrates were highly pure, with more than 90% of measured monosaccharides attributable to fucoidan, and fucose comprising 63–96% of these (Fig. 3d, Extended Data Fig. 10a and Supplementary Table 9). Two preparations (*D. potatorum* and *F. serratus*), however, contained elevated glucose levels (45% and 73%, respectively), potentially from co-extracted laminarin, which was almost completely depleted across communities, showing rapid consumption of labile glucans (Extended Data Fig. 10b). By contrast, fucoidan degradation depended strongly on community composition: simpler communities showed limited degradation, consistent with constraints imposed by incomplete enzymatic repertoires. Also consistent with our previous observations, communities that only contain rare-sugar specialists F12, F40 and F94 achieved higher degradation on substrates enriched in rare-sugar monomers (Extended Data Fig. 10c).

Remarkably, without any refitting, our predictive model generalized well to these chemically distinct fucoidans (*R*^2^ = 0.90, *P* < 0.001; Fig. 3e). Using the parameters trained on fucoidan from *F. vesiculosus*, the model accurately predicted degradation across all substrates by accounting solely for the stoichiometry of fucose and rare-sugar monomers. These out-of-sample predictions demonstrate an extraordinary degree of accuracy, considering the complexity of the degradation pathways involved. This suggests that the metabolic capabilities of the degraders and their synergistic interactions remain conserved across different substrates, despite variation in overall monomer composition and polysaccharide structure.

## Global metagenomic evidence for synergism

**Fig. 4:**
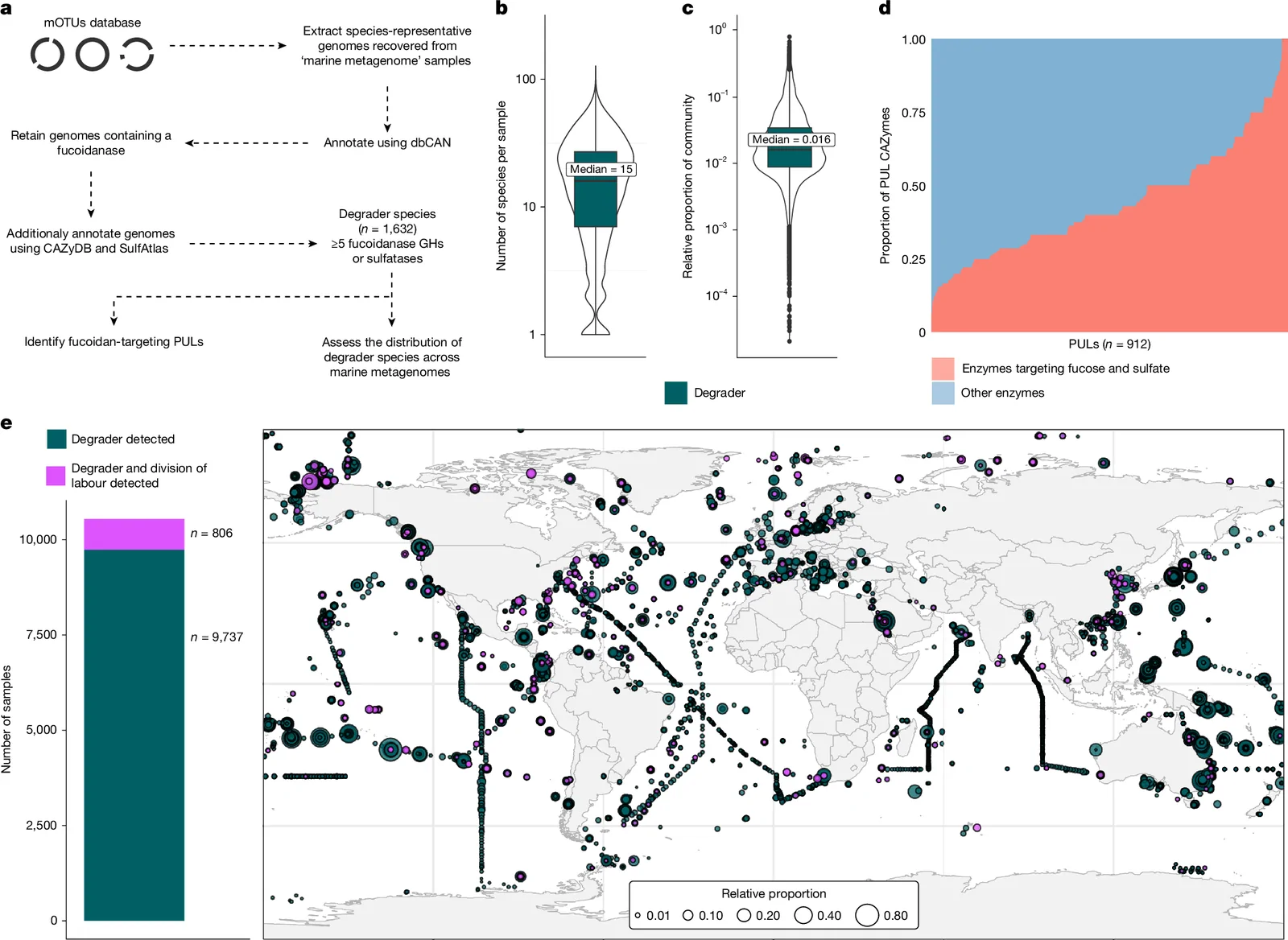
Potential for synergistic fucoidan degradation in marine microbial communities. **a**, Overview of the analysis workflow used to identify fucoidan-degrading species and their functional repertoires across marine metagenomes. GH, glycoside hydrolase. **b**,**c**, Distribution of the number of degrader species (**b**) and their relative proportion of the community per sample (**c**) (from 10,543 metagenome samples), showing that multiple degraders commonly co-occur within individual communities and represent a consistent but typically low-abundance fraction. Box plots illustrate the 25th, median and 75th percentile of the distribution, with whiskers representing minimum and maximum values. Relative proportion is defined as the proportion of genomes within each community that is attributed to degraders, determined through single-copy marker gene coverage. **d**, Enzymatic composition of fucoidan-targeting PULs, illustrating a continuum of sulfated fucose and rare-sugar monomer targeting PULs. **e**, Right, global distribution of metagenome samples (*n* = 10,543) containing degrader species (teal) and samples with co-occurring degraders exhibiting complementary functional profiles (purple). Left, bar plot summarizing the number of samples in which degraders are detected and the subset in which potential for synergistic degradation is observed.

So far, we have seen that fucoidan degradation is controlled by synergistic interactions among degraders, rooted in the complementarity of their enzyme repertoires. To assess whether these interactions constrain fucoidan cycling at a global scale, we leveraged metagenomic data to quantify the abundance and co-occurrence patterns of organisms classified as degraders (Fig. 4a–c). In 12,347 ocean metagenomes from the mOTUs database^55^, we identified 1,632 putative degrader species encoding between 5 and 175 fucoidanase genes. Degrader abundance and prevalence exhibited highly right-skewed distributions (median relative abundance: 1.6%, maximum: 33%), consistent with previous reports of *Lentimonas* spp.^3,56^. Critically, multispecies assemblages were near-universal: 94% of samples harboured multiple degrader species (median: 15 species per sample). This ubiquity of co-occurring degraders across diverse ocean habitats suggests that the synergistic interactions identified in culture are likely to constrain fucoidan flux at global scales.

To quantify the potential for synergism among co-occurring degraders, we focused on the 528 species for which we identified fucoidan-targeting PULs. Variations in the enzyme composition of these PULs recapitulated the functional specialization observed in our isolates, encompassing repertoires enriched in enzymes targeting sulfate and fucose linkages or other enzymes that potentially act on rare-sugar monomers (Fig. 4d). Leveraging these variations, we conservatively estimated signals of synergism across ocean samples based on the co-occurrence of degraders with high-capacity (upper quartile) and low-capacity (lower quartile) of fucose-targeting PULs. This analysis identified 806 samples containing both functional extremes and thus a potential synergism (Fig. 4e). Together, these findings suggest that functionally specialized and complementary fucoidan degraders frequently co-occur in the ocean, supporting synergistic interactions as an ecologically relevant strategy for the degradation of complex marine fucoidans.

## Discussion

Our results demonstrate that positive interactions among complementary degraders are both frequent and essential for the complete breakdown of fucoidan by marine microbial communities. This emphasizes that its degradation is fundamentally a collective effort. Despite being a particularly complex and variable class of polysaccharides—with dozens of glycosidic linkages and an expansive enzymatic space—the degradation of fucoidans at the community level proves remarkably predictable. This paradox is resolved by synergism among degraders, in which individual strains consistently specialize in either the fucose-rich backbone or the side chains of rare sugars, exhibiting conserved metabolic roles across diverse fucoidan structures. This allows for a quantitative mapping of community composition to degradation outcomes and underscores that the co-evolutionary arms race between the protective extracellular matrix of brown algae and microbial degraders unfolds at the monomer level.

Degradation synergism does not necessarily emerge from mutualistic interactions, but rather depends on complementarity between enzyme repertoires. The single exception was the mutualistic V69–G88 pair, indicating that genuine cooperative division of labour, while rare, can occur in these systems. Evolutionary and ecological pressures appear to have driven the partitioning of complementary enzymes across organisms. Several non-mutually exclusive hypotheses are plausible. One possibility involves a metabolic trade-off between fucose and other hexoses: whereas side-chain sugars typically feed into upper glycolysis, fucose enters metabolism at the level of lower glycolysis, requiring gluconeogenic flux to generate upstream intermediates. This mismatch may favour the evolution of specialists that target either the fucose backbone or the side chains, avoiding potentially wasteful cycling in central carbon metabolism^57,58^. Another contributing factor may be the metabolic cost of maintaining and regulating a broad enzymatic arsenal. Fully self-sufficient degraders must coordinate the expression of hundreds of genes, many of which are needed to process low-abundance monomers, making this strategy energetically inefficient. Finally, the continual loss of genes through neutral processes such as genetic drift, together with the capacity of coexisting degraders to compensate for missing functions, suggests that complete degraders may be evolutionarily unstable. By contrast, gene loss may drive the recurrent emergence of complementary types that together achieve full degradation.

Our findings have implications for both microbial ecology and biotechnology. First, they highlight the challenges associated with engineering single strains to degrade chemically complex substrates such as fucoidan, where identifying precise enzyme functions proves difficult. By contrast, harnessing microbial communities with pre-evolved metabolic complementarity presents a powerful and scalable alternative for biomass conversion. Thus, our work establishes a conceptual and practical framework to process increasingly important brown algal biomass and might extend to other polysaccharides with similar structures such as xylans^21^. Second, from an ecological perspective, our experimental and metagenomic analyses suggest that fucoidan degradation frequently emerges from assemblies of co-occurring degraders with complementary enzymatic repertoires. This reliance on the assembly of complementary partners may help explain how microbial diversity shapes fucoidan turnover and influences its persistence and contribution to marine carbon storage. Our research highlights an underrecognized link between microbial diversity, polysaccharide turnover and global biogeochemical cycles. In light of microbial communities responding to global ocean warming^59^, shifting community structures may ultimately influence the ocean’s functional capacity for carbon sequestration.

## Methods

### Chemicals and reagents

Fucoidan from *F. vesiculosus* (Sigma-Aldrich, F8190, lot no. 0000485452) was used as the primary substrate throughout this study. Additional *F. vesiculosus* fucoidans were obtained from Marinova (FVF2021547) and Biosynth (YF57714). Fucoidans from other species were sourced from *Fucus serratus* (Biosynth, YF09360), *Fucus evanescens* (OceanBasis and extracted as described previously^60^), *Cladosiphon okamuranus* (Biosynth, YF146834), *Durvillaea potatorum* (Biosynth, YF157165), *Ecklonia maxima* (Biosynth, YF157166) and *Laminaria hyperborea* (TheFucoidanStore, LowEndo Fucoidan). For derivatization, 1-phenyl-3-methyl-5-pyrazolone (PMP) was obtained from Sigma-Aldrich (M70800). Internal standards for LC–MS analysis included d-galactose-^13^C_6_ (Sigma-Aldrich, 605379), d-mannose-^13^C_6_ (Sigma-Aldrich, 592994) and PMP-[d_5_] (CAS 1228765-67-0), custom-synthesized by BOC Sciences (Shirley). LC–MS-grade solvents and reagents were acetonitrile (Honeywell), methanol (Honeywell), ethanol (Sigma-Aldrich), formic acid (Sigma-Aldrich) and ammonium formate (Merck). Ultrapure water was produced using a Q-POD system (Merck). Unless otherwise stated, all other chemicals were of analytical grade and sourced from Sigma-Aldrich.

### Bacterial growth media

Throughout this study, three distinct media detailed in Supplementary Table 1 were used for (1) enrichment of bacteria, (2) isolation of bacteria on solid medium and (3) routine cultivation of bacterial isolates in MBL medium^52^. All media mimicked the ionic composition of coastal seawater and contained 340 mM NaCl, 15 mM MgCl_2_, 6.75 mM KCl and 1 mM CaCl_2_. The pH was buffered to 8.0 with either bicarbonate in the enrichment and plate media or 50 mM HEPES in the MBL medium. Nutrients were ammonium chloride, sodium phosphate, sodium sulfate, trace metal mix and vitamin mix^52^. Enrichment medium contained 0.02% (w/v) fucoidan from *F. vesiculosus*. Solid medium was prepared with 2% (w/v) carrageenan (Sigma C1013) and a mix of carbon sources (acetate, citrate, xylose, galactose, mannose, glucose, fucose, cellobiose and tryptone) at 0.002% (w/v) each. Bacterial isolates were routinely cultured in MBL medium with carbon sources specific to their metabolic requirements. Verrucomicrobiota degraders were routinely grown with 0.2% (w/v) fucoidan from *F. vesiculosus*. Other degraders (Flavobacteriia and Gammaproteobacteria) and exploiters were grown on a mix of 0.2% (w/v) l-fucose and 0.2% (w/v) fucoidan from *F. vesiculosus*. Scavengers were grown in a mix of carbon sources (acetate, citrate, xylose, galactose, mannose, glucose, fucose, cellobiose and tryptone) at 0.02% (w/v) each.

### Enrichment of fucoidan-degrading communities

Surface seawater was collected on 30 March 2019 from a rocky shoreline covered with the brown algae *F. vesiculosus*, *Ascophyllum nodosum* and *Saccharina latissima* near the Marine Science Center of Northeastern University (Canoe Beach, Nahant, MA, USA; 42° 25′ 10.8732″ N, 70° 54′ 25.686″ W). The seawater was pre-filtered through a 10-μm PTFE membrane filter (Millipore, JCWP04700) and diluted 1:100 to inoculate three replicate enrichment cultures (25 ml each) containing 0.02% (w/v) *F. vesiculosus* fucoidan in 150 ml glass bottles sealed with rubber stoppers. Cultures were incubated at 20 °C in the dark and agitated at 50 rpm.

Enrichment cultures were monitored every 24–48 h for microbial growth (OD_600_) and fucoidan degradation, quantified via the phenol–sulfuric acid method^61^. For each measurement, 200 µl of culture was mixed with 1 ml of concentrated sulfuric acid and 200 µl of 5% (v/v) phenol, then incubated at 50 °C for 20 min. Absorbance was measured at 490 nm and fucoidan concentration was determined using an external standard curve of fucose. After 10 days, cultures reached 40–60% degradation of the initial fucoidan, at which point serial growth–dilution cycles were initiated by transferring 1:50 into freshly prepared medium every 2 days for a total of 12 cycles. At every second time point, 10 ml of culture was filtered onto a 0.22 μm Sterivex filter (Millipore, SVGPB1010) for DNA extraction using the DNeasy Blood & Tissue Kit (Qiagen) and subsequent metagenomic sequencing. Final enrichment communities were cryopreserved at −80 °C with 15% (v/v) glycerol.

### Isolation of bacterial strains

From each enrichment culture, cells were enumerated using a counting chamber by light microscopy. Aliquots corresponding to 10^2^, 10^3^ and 10^4^ cells were plated in triplicate onto solid medium in 150 mm Petri dishes (VWR 391-0616) and incubated for 14 days at ambient temperature in the dark. A total of 768 colonies were picked and re-streaked at least 3 times until only a single colony morphotype was observed. Colonies were lysed in 0.1% (v/v) Triton X-100 in TE buffer for Sanger sequencing of the 16S rRNA gene. Pure isolates were grown in MBL medium with a mix of carbon source and cryopreserved at −80 °C in 15% (v/v) glycerol. To de-replicate repeatedly isolated strains, we selected 96 isolates based on their 16S sequences for genomic DNA extraction using the DNAadvance kit (Beckman Coulter) and draft genome sequencing. To identify redundant isolates, pairwise average nucleotide identity (ANI) was computed using OrthoANIu v1.2^62^ yielding 28 unique bacterial strains. We additionally included the characterized degrader ‘*Lentimonas’* sp. CC4 as Verruco4 in the isolate collection^3^ resulting in a total of 29 strains in the isolate collection.

### Sequencing of metagenomes and isolate genomes

All sequencing work was carried out in collaboration at the BioMicroCenter at MIT. Metagenomes of enrichment cultures were sequenced with an Illumina NovaSeq6000 S4 flow cell with 150 nt paired-end reads. Metagenomes were assembled using SPAdes v3.13.0^63^ with the parameters --meta --only-assembler -k 21,33,55,77. MAGs were reconstructed using CONCOCT v1.0.0^64^, MaxBin v2.2.7^65^ and DAS Tool v1.1.2^66^ with default settings. Draft genomes of isolates were sequenced on an Illumina NextSeq 500 platform (150 nt paired-end reads, ~200× coverage) and assembled using SPAdes v3.13.0 with the parameters --only-assembler -k 21,33,55,77 --careful. To generate closed genomes for selected degraders (Flavo12, Flavo40, Flavo56, Flavo94, Gamma88, Verruco25 and Verruco69), genomic DNA was extracted from 1 ml of culture using the DNeasy Blood & Tissue Kit (Qiagen). Long-read sequencing was performed on Nanopore PromethION FLO-PRO002 flow cells. Hybrid assemblies combining Illumina short reads and Nanopore long reads were generated using Unicycler v0.4.8^67^ with the parameters --keep 3 --mode normal --min_fasta_length 1000 --kmers 77, yielding circularized genome assemblies. Completeness and contamination of MAGs was assessed using CheckM v1.1.2^68^ resulting in a total of 73 medium and high-quality genomes^69^ summarized in Supplementary Table 2. Taxonomic classification and phylogenetic reconstruction of isolate and metagenome-assembled genomes were performed using GTDB-Tk v2.0.0^70^ (release 202).

### Annotation of bacterial genomes

Open reading frames were predicted and annotated with DRAM v1.2.4^71^. CAZymes were identified by HMMER^72^ v3.3 with hidden Markov models from the dbCAN database^73^ (dbCAN-HMMdb-V10). HMM hits were validated via Diamond^74^ (v0.9.14.115; blastp mode, --more-sensitive) against the CAZy database (accessed September 2022), retaining only matches with bit scores >100. Sulfatases were identified by hmmsearch against PF00884 (sulfatase domain), using an e-value threshold of <10^−4^ and a minimum alignment length of 100 amino acids. Sulfatases were further classified into subfamilies based on Diamond (v0.9.14.115; blastp mode, --more-sensitive) against the SulfAtlas v1.2. database^75^.

### Classification of genomes into metabolic roles

We classified genomes into three metabolic roles, degraders, exploiters and scavengers, based on the presence or absence of key enzymes involved in fucoidan and l-fucose metabolism. Degraders were defined as genomes encoding at least five enzymes from a curated set of ten families of fucoidanase families^3^, comprising glycoside hydrolases (GH29, GH95, GH141, GH107 and GH168) and sulfatases (S1_15, S1_16, S1_17, S1_22 and S1_25). Exploiters lacked fucoidanases but encoded one of two alternative l-fucose catabolic pathways described in MetaCyc^57^: l-fucose degradation I or l-fucose degradation II. For each pathway, genomes were required to encode at least 75% of the constituent enzymes. For pathway I, this included: K07248 (lactaldehyde dehydrogenase), K02431 (l-fucose mutarotase), K00879 (l-fuculokinase), K01818 (l-fucose/d-arabinose isomerase) and K01628 (l-fuculose-phosphate aldolase). For pathway II, this included: K18333 (l-fucose dehydrogenase), K18334 (l-fuconate dehydratase), K18335 (2-keto-3-deoxy-l-fuconate dehydrogenase), K07046 (l-fuconolactonase), K18336 (2,4-didehydro-3-deoxy-l-rhamnonate hydrolase) and K01685 (altronate hydrolase). Scavengers were defined as genomes lacking all enzymes associated with both fucoidan degradation and l-fucose catabolism.

### Identification of fucoidan PULs

We searched closed genomes of cultured fucoidan degraders for candidate PULs using a sliding 12-gene window. Regions containing at least four CAZyme genes were flagged and those encoding two or more known fucoidanases (GH29, GH95, GH141, GH107, GH168, S1_15, S1_16, S1_17, S1_22 or S1_25) were retained as putative fucoidan PULs. To avoid misclassification, we manually curated these loci and removed those likely involved in the degradation of other polysaccharides—specifically, five loci from Flavobacteriia containing carrageenanases (GH16, GH82, GH150 and GH167). The final curated set comprised 34 fucoidan-associated PULs spanning 54 CAZyme families (Supplementary Table 3).

### Definition and comparison of fucoidan enzyme repertoires

For each degrader, the fucoidanase repertoire was defined as (1) all CAZymes and sulfatases within fucoidan PULs and (2) additional chromosomal genes belonging to enzymes families that target sulfated fucose (GH29, GH95, GH141, GH107, GH168, S1_15, S1_16, S1_17, S1_22 or S1_25) or that are predicted to act on rare sugars (GH30, GH31, GH36, GH39, GH92, GH97, GH115 and GH120). For the characterized degrader^3^, *Lentimonas* sp. CC4, only enzymes that were upregulated at the protein level during growth on *F. vesiculosus* fucoidan were included, specifically those in co-expression clusters 2–6.

To sort the sequence diversity into homologous groups of enzymes, we clustered all fucoidan-associated CAZymes using MMseqs2 v13.45111 (easy-cluster with --min-seq-id 0.6 -c 0.5 --cov-mode 0)^76^. This defined enzyme homologues at a threshold of ≥ 60% amino acid identity ≥ 50% alignment coverage for both query and target sequences. These clusters formed the basis for showing counts of enzyme families and comparing enzyme repertoires across isolates and pairwise similarities were calculated using the Jaccard index.

To explore how enzymatic diversity scales with community size, we performed a rarefaction analysis. In each of 10,000 iterations, we randomly selected *n* degrader strains (*n* = 1–29), counted the number of unique enzyme clusters, and estimated the expected total richness using the Chao1 estimator.

### Refined functional annotation of fucoidan-active CAZymes

The activities of fucoidan-associated enzymes found in isolated degraders were inferred by comparison to characterized CAZymes in the CAZy database (accessed March 2026) using Diamond (v0.9.14.115; blastp mode, --more-sensitive). For each domain, the top-scoring hit was used to assign putative function and EC number, where available. Proteins containing multiple catalytic domains were annotated at the domain level. Annotation confidence was defined as high (≥ 70% identity and ≥70% coverage), medium (≥ 30% identity and ≥50% coverage) and otherwise retained as family-level assignments.

Enzymes were then assigned to coarse-grained activity classes when the inferred EC number, CAZyme family or closest characterized homologue indicated an activity compatible with known fucoidan linkage chemistry. These classes included fucose-, galactose-, mannose-, xylose- and glucuronic acid-targeting activities. Specifically, GH107, GH141 and GH168 were classified as endo-acting fucoidanases; GH29 and GH95 as exo-α-l-fucosidases; GH36 and GH97 as α-galactosidases; GH2 as β-galactosidases; GH92 as α-mannosidases; GH115 as α-glucuronidases; and GH31, GH39, GH120 and GH30 as α-/β-xylosidases. Enzyme families without support for an activity on known fucoidan residues were classified as hypothetical. All inferred activities, EC numbers and annotation confidence levels are reported in Supplementary Table 3.

Phylogenetic validation of representative families was performed using MAFFT (L-INS-i; --localpair --maxiterate 100) for alignment, trimAl (-gt 0.1) for trimming and FastTree (LG + Γ model) for tree inference.

### Characterization of fucoidan-degrading abilities in monoculture

Substrate utilization and fucoidan-degrading capabilities of all 29 isolates were assessed by growth assays on defined carbon sources. Strains were revived from glycerol stocks in 3 ml MBL medium for 3–6 days using carbon sources matched to their metabolic requirements. Cultures were subsequently washed in carbon-free MBL medium and inoculated into 200 μl of fresh medium supplemented with a single carbon source at 0.2% (w/v), including l-fucose, d-galactose, d-mannose, d-xylose, d-glucuronic acid, or fucoidan from *F. vesiculosus*. Cultivations were performed in biological triplicates (*n* = 3) at 20 °C with orbital shaking (200 rpm) in 96-well microtiter plates (flat-bottom, polystyrene; Corning). Growth was monitored by OD_600_ measurements over 5 days using a Tecan Sunrise plate reader. To quantify fucoidan degradation, cultures grown on fucoidan were sampled at the final time point in early stationary phase. Cell-free supernatants were obtained by centrifugation (2,200 rpm for 10 min), remaining fucoidan was hydrolysed and quantified as described below.

For time-resolved characterization, the eight degraders were cultivated in 4 ml MBL medium supplemented with fucoidan from *F. vesiculosus* in 24-deep-well plates (Eppendorf) sealed with breathable lids (Kuhner) and sampled throughout growth. At each time point, supernatants were collected and analysed to quantify both fucoidan-derived monomers following acid hydrolysis and free extracellular monosaccharides.

### Acid hydrolysis of fucoidan in supernatants

To quantify the decrease of fucoidan-bound monomers after growth, acid hydrolysis was used to cleave the glycosidic linkages of fucoidan remaining in culture supernatants releasing its monomer constituents. Sampled supernatants (5 μl) were mixed with 45 μl of ddH_2_O and 50 μl of 2 M HCl. The HCl solution contained d-galactose-^13^C_6_ and d-mannose-^13^C_6_ at 15 μM each that were later used as ‘processing internal standards’ to correct technical variability introduced by the sample processing workflow. PCR plates with samples were sealed with plastic strips (Thermo Fisher Scientific AB0600 and AB0784). Acid hydrolysis was carried out for 24 h at 100 °C in an oven using a custom clamping device to prevent leakage from plates. After hydrolysis, samples were neutralized by addition of 4 M NaOH.

### Derivatization of monosaccharides with PMP

Acid hydrolysates (10 μl sample and 15 μl ddH_2_O) or free monosaccharide samples (25 μl sample) were derivatized with 75 μl of 0.1 M PMP in 2:1 methanol:ddH_2_O with 0.4 M ammonium hydroxide for 100 min at 70 °C following a previously published protocol^47^. For absolute quantification, we used an external calibration curve of a standard mix containing glucuronic acid, xylose, fucose, galactose, mannose ranging from 1 mM to 200 nM prepared in a matrix identical to samples. After derivatization, samples and standards were neutralized with 2 M HCl and diluted 1:50 in 0.1% (v/v) formic acid in ddH_2_O containing 50 nM of injection internal standards, which was used to correct variations in ionization efficiency caused by the ion source of the mass spectrometer. These internal standards consisted of a mix of glucuronic acid, xylose, fucose, galactose, mannose derivatized with heavy labelled PMP-[d5] yielding unique masses distinct from unlabelled PMP.

### Targeted acquisition of PMP derivatives with LC–MS

PMP derivatives were measured on a SCIEX qTRAP5500 and an Agilent 1290 Infinity II LC system. The system was equipped with a Waters CORTECS UPLC C18 Column, 90 Å, 1.6 μm, 2.1 mm × 50 mm reversed phase column with guard column and 0.2 μM inline filter. The mobile phase consisted of buffer A with 10 mM NH_4_Formate in ddH_2_O and 0.1% (v/v) formic acid and buffer B with 100% acetonitrile and 0.1% (v/v) formic acid. PMP derivatives were separated by an initial isocratic flow of 13% buffer B for 30 s, followed by a binary gradient from 13% to 36% Buffer B over 2 min, followed by a 30 s wash step with 100% buffer B and 30 s re-equilibration. The flow rate was constant at 0.5 ml min^−1^ with a constant pressure around 430 bar. A diverter valve was used to redirect the first 1.5 min of chromatography to waste. The temperature of the column compartment was maintained at 40 °C and the autosampler was cooled to 10 °C. The ESI source settings were 625 °C, with curtain gas set to 30, collision gas to medium, ion spray voltage to 5500 and ion source gas 1 and 2 to 90 (arbitrary units).

Data were acquired using multiple reaction monitoring with previously optimized transitions and collision energies in positive mode^46^. For example, a galactose derivative has an exact Q1 mass of 511.2 *m*/*z* and was fragmented with a collision energy of 35 V to yield the quantifier ion of 175.0 *m*/*z* and the diagnostic fragment of 217.2 *m*/*z*. All multiple reaction monitoring transitions and retention times used to identify compounds are listed in Supplementary Table 4.

Typically, each run included 150–250 samples, with randomized injection order. For quality control (QC) samples, we used the highest concentrated standard mix. QC samples and a water blank were injected every 15 samples to monitor consistency and carryover. Calibration curves were prepared in triplicate and each sample was analysed in technical duplicates. Guard columns and inline filters were replaced every 500 to 1,000 injections.

### Absolute quantification of monosaccharides

Chromatographic data were analysed using Skyline^77^ v24.1.0.414, with peak areas integrated using default settings and exported for downstream quantification in Python. A reproducible example workflow is provided under https://github.com/EnvSysMicroLAB/Sichert2025_Fucoidan. In brief, peak areas of target compounds and ^13^C-labelled processing standards were first corrected using the injection internal standards. Subsequently, the corrected ^13^C processing standards were used to normalize the target compound signals. Absolute concentrations of monomers were determined by linear regression against external calibration curves and technical duplicates were averaged to obtain final concentrations.

### Quantification of monomer degradation

To quantify fucoidan degradation at the monomer level, concentrations of the five constituent sugars—fucose, glucuronic acid, galactose, mannose and xylose—in culture supernatants were compared to an uninoculated medium control processed in parallel. Notably, the concentrations of ‘rare’ monomers are calculated from the sum of the four non-fucose sugars, while ‘total’ monomers represent the sum of all five monomers. Degradation *f* of a given monosaccharide *i* (*i* ∈ {Fuc, GlcA, Gal, Man, Xyl, Rare, Total}) by strain or strain combination *j*, was quantified as:

1 $${f}_{i,j}=100\times \frac{{[{\rm{S}}]}_{i,{\rm{c}}{\rm{o}}{\rm{n}}{\rm{t}}{\rm{r}}{\rm{o}}{\rm{l}}}-{[{\rm{S}}]}_{i,j}}{{[{\rm{S}}]}_{i,\mathrm{control}}}$$

where [S]_*i*,control_ is the concentration in the uninoculated control and [S]_*i*,*j*_ is the concentration after growth of strain *j* and *f*_*i*,*j*_ is the relative monomer degradation.

For every strain–monomer combination, we tested whether the observed decrease in concentration exceeded abiotic background using a one-sided *t*-test (alternative = “less”). Resulting *P* values were corrected for multiple comparisons using the Benjamini–Hochberg false discovery rate (FDR) procedure (*α* = 0.01). Degradation was considered significant when both the raw *P* values and the FDR-adjusted *q* were <0.01. Throughout this study, we generated seven distinct datasets, with monosaccharide concentrations and corresponding statistical analyses reported in Supplementary Table 4. Unless otherwise stated, all statistical analyses of degradation data involving Pearson correlation coefficients and associated *P* values were performed using replicate-level data from three biological replicates, without prior aggregation into means.

### High-throughput community experiments

To assess the effect of biological interactions on fucoidan degradation, we performed four high-throughput growth experiments using a standardized cultivation setup. All experiments were conducted in biological triplicates (*n* = 3) at 20 °C with orbital shaking at 200 rpm. Glycerol stocks were revived in 3 ml of MBL medium in plastic culture tubes for 3–6 days, using carbon sources matched to the metabolic requirements described above. Revived cultures were diluted 1:5 into fresh medium in 96-deep-well plates (1.8 ml per well), each containing a 4 mm glass bead (Sigma-Aldrich, Z143936) and incubated for 18 h to reach exponential phase (OD_600_ = 0.15–0.3). Exponential-phase cells were washed in carbon-free MBL and used to inoculate experimental cultures at an initial OD_600_ of 0.01 for each strain. Experimental cultures were incubated for up to 5 days in the same deep-well plate format, sealed with breathable lids (Kuhner, 104098 and 104106). At defined time points, 100 μl of culture was sampled for OD_600_ measurement using a Tecan Sunrise plate reader. Samples were centrifuged at 2,200 rpm for 10 min to separate cells and supernatant; supernatants were stored at –20 °C for further analysis.

### Verrucomicrobiota-centric pairwise co-cultures

To assess the influence of community members on the degradation activity of primary degraders, each of the 29 isolates was co-cultured in a 1:1 ratio (normalized by OD_600_) with one of the three primary degraders (V4, V25 or V69), yielding 87 distinct pairwise combinations. Cultures were incubated for 5 days and fucoidan degradation was quantified at the final time point. Growth dynamics in co-cultures were assessed using two complementary metrics: (1) total biomass, defined as the mean of the two highest OD_600_ values recorded during the time course and (2) apparent growth rate, calculated as the inverse of the time required to reach an OD_600_ threshold of 0.15 (1/*t*_th_). In co-cultures of V69 with strains F12, F40, F56 and G88, we observed aggregate formation that interfered with OD_600_-based biomass estimates. For these cases, we used an indirect approximation for bacterial biomass by quantifying bacterial DNA from 100 μl of culture using the DNAdvance Kit (Beckman Coulter) followed by absolute DNA quantification with the Femto Bacterial DNA Quantification Kit, according to the manufacturer’s protocol.

To identify potential synergistic or antagonistic effects in pairwise co-cultures, we compared biomass formation and fucoidan degradation to the corresponding monocultures. Differences in biomass were considered significant if both the *P* value and the false discovery rate (FDR, Benjamini–Hochberg correction, *α* = 0.01) were <0.01. Differences in degradation were considered significant if both the *P* value and FDR (*α* = 0.05) were <0.05 and the absolute log_2_-transformed fold change exceeded 0.1.

### qPCR for strain-specific cell quantification

To determine strain-specific abundances in co-culture, 100 µl of co-cultures were sampled and subjected to two freeze–thaw cycles prior to DNA extraction using the DNAdvance Kit (Beckman Coulter, A48705). Strain-specific qPCR primers were designed against ~100-bp regions of single-copy marker genes. qPCR reactions were performed in 20 µl volumes using GoTaq qPCR Master Mix (Promega) on a QuantStudio 3 Real-Time PCR System. Primer efficiencies ranged from 95–105%, and primer specificity and quantitative recovery were assessed in defined mock communities comprising all strains at known input ratios, yielding recoveries of 90–127% (Supplementary Table 6). Absolute abundances were quantified by comparison of *C*_t_ values to strain-specific standard curves generated from serial dilutions of genomic DNA (10 ng µl^−1^ to 10 pg µl^−1^). Resulting values represent strain-specific DNA concentrations, which were used to compare relative changes between monoculture and co-culture conditions and to classify interaction outcomes^48^.

### Synergism beyond resource partitioning

To assess synergistic interactions in pairwise co-cultures, we formulated a null model that estimates expected degradation outcomes based on monoculture performance, assuming no metabolic complementarity between strains. This model reflects a scenario in which both strains have fully overlapping substrate preferences, such that each monomer can be degraded only up to the level achieved by the better-performing monoculture. The difference between this null expectation and the observed co-culture degradation defines the synergism score *σ*(*i*,*j*), formally given as:

2 $$\sigma (i,j)={f}_{\mathrm{total}}(i,j)-\sum _{m\in \{{\rm{F}}\mathrm{uc},\mathrm{Man},\mathrm{Gal},\mathrm{Xyl},\mathrm{GlcA}\}}\max ({f}_{m}(i),{f}_{m}(j))$$

where *f*(*i*,*j*) is the total fraction of fucoidan degraded in co-culture of strains *i* and *j*, *f*_*m*_(*i*) and *f*_*m*_(*j*) are the fractions of monomer *m* degraded by strains *i* and *j* in monoculture. Note that all terms are expressed as stoichiometrically weighted fractions rather than raw percentages; that is, each monomer-specific contribution *f*_*m*_ is scaled by its relative abundance in the fucoidan composition before summation. This conservative formulation avoids overestimation of expected degradation by preventing redundant contributions from metabolically similar strains. Positive values of *σ*(*i*,*j*) thus indicate synergism through functional complementarity, whereas negative values suggest antagonism. To determine whether observed co-culture degradation significantly deviated from the null expectation, we applied a two-sided *t*-test. Resulting *P* values were corrected for multiple comparisons using the Benjamini–Hochberg procedure, and synergism scores were considered significant if they satisfied *P* < 0.01, *q* < 0.01, and exhibited an absolute effect size greater than 5 (|*σ*| > 5). Notably, this framework can be extended to communities with more than two members by using the maximum monomer degradation observed in the best-performing drop-out community as the null expectation.

### Cross-feeding of residual fucoidan

Partially degraded fucoidans were recovered from cultures of V4, V25 and V69 grown to late stationary phase in 1 l of MBL medium supplemented with 0.2% (w/v) fucoidan from *F. vesiculosus*. The sterile filtered supernatant was concentrated using an Amicon stirred ultrafiltration cell (EMD Millipore, UFSC20001) with a 1-kDa cellulose membrane (EMD Millipore, PLAC06210) on ice using nitrogen for gas pressure. Desalting was carried out by addition of ddH_2_O and continued concentration. Concentrated material was lyophilized.

To test how ‘secondary’ degraders could utilize the residual substrates of primary degraders, F12, F40, F56, F94, V25, V69 and V4 were grown in 1.5 ml of MBL medium containing 0.1% (w/v) of each residual fucoidan. Cultures were incubated for 4 days and degradation was quantified from supernatants collected at the final time point compared to initial concentrations.

To compare the additional degradation of residuals achieved by secondary degraders to the additional degradation observed in co-cultures, we used the relationship:

3 $${f}_{{\mathrm{total},\mathrm{residual}}_{j}}(i)={f}_{\mathrm{total},\mathrm{untreated}}(i,j)-{f}_{\mathrm{total},\mathrm{untreated}}(j)$$

Here, *f*_total,residual_(*i*) denotes the degradation of secondary degrader *i* on residuals of primary degrader *j*; *f*_total,untreated_(*i*,*j*) is the total degradation observed in co-culture of strains *i* and *j* on untreated fucoidan; and *f*_total,untreated_(*j*) is the degradation by strain *j* alone on the same substrate.

### Full combinatorial degrader communities

To systematically evaluate synergistic interactions in fucoidan degradation, we constructed all 127 possible combinations of the seven degraders F12, F40, F56, F94, V25, V69 and V4, along with a no-cell negative control. Each community was assembled by mixing strains at equal optical density (OD_600_) at 0.01 each and inoculating into 1.8 ml of MBL medium supplemented with 0.2% (w/v) fucoidan from *F. vesiculosus* as the sole carbon source. Cultures were incubated for 5 days (*n* = 3 biological replicates), after which supernatants were collected for degradation analysis.

### Modelling

To predict degradation from community composition, we developed a nonlinear trait-based model in which each bacterial strain contributes independently to the degradation of two classes of fucoidan-derived monomers: fucose and rare sugars. Each strain *i* was assigned two parameters (${w}_{i}^{{\rm{Fuc}}}$ and ${w}_{i}^{{\rm{Rare}}}$) representing its degradation capacity for these monomer types. For a given community composed of *m* strains, represented by a presence/absence vector *x*_*i*_ ∈ {0,1}, the total effective capacity to degrade each monomer pool was computed as the sum of contributions from all present strains:

4 $${C}^{\mathrm{Fuc}}=\mathop{\sum }\limits_{i=1}^{m}{w}_{i}^{\mathrm{Fuc}}\times {x}_{i}$$

To capture the saturating behaviour observed in experimental data, we applied a Hill function to these summed capacities, introducing nonlinearity and a threshold-like response:

5 $${D}^{\mathrm{Fuc}}=\frac{{({C}^{\mathrm{Fuc}})}^{n}}{{K}_{{\rm{m}}}^{n}+{({C}^{\mathrm{Fuc}})}^{n}}$$

6 $${D}^{\mathrm{Rare}}=\frac{{({C}^{\mathrm{Rare}})}^{n}}{{K}_{{\rm{m}}}^{n}+{({C}^{\mathrm{Rare}})}^{n}}$$

Here, ${K}_{{\rm{m}}}^{n}$ is the half-saturation constant and *n* is the Hill coefficient, both shared across the two monomer classes. The resulting values *D*^Fuc^ and *D*^Rare^ represent the predicted fraction of each monomer pool degraded by the community.

Experimental degradation values of 127 communities were used to fit the model parameters: one degradation capacity per strain and per monomer pool (${w}_{i}^{{\rm{Fuc}}}$ and ${w}_{i}^{{\rm{Rare}}}$) and shared parameters ${K}_{{\rm{m}}}^{n}$ and *n*. Model parameters were inferred by minimizing the root mean squared deviation between predicted and observed degradation. Optimization was performed in Julia using the LsqFit.jl package. Initial and final parameter values, as well as their effect on model fit are shown in Extended Data Fig. 9a–c. Goodness-of-fit was assessed via visual comparison and *R*^2^ statistics (Extended Data Fig. 9d). All code and data used in model construction and fitting are available here https://github.com/EnvSysMicroLAB/Sichert2025_Fucoidan.

### Degradation of diverse fucoidans

To evaluate the degradation capabilities of individual degraders across diverse fucoidans, we assembled a panel of eight additional brown algal fucoidans. These included fucoidans derived from six brown algal species selected for their differences in composition and structure, as well as two variants of *F. vesiculosus* sourced from two different vendors. The latter were included to capture subtle compositional and structural variations suspected to arise from differences in sampling time, sampling season and extraction method^6,33^.

For community experiments, degraders were grouped to minimize redundant combinations and maximize functional complementarity: Group A (V25, V4 and F56), Group B (F12, F40 and F94) and Group C (V69). These groups were tested individually, in all pairwise combinations and as a triplet (seven configurations total). Each community was inoculated with equal OD_600_ contributions from each strain into 1.8 ml of MBL medium containing 0.2% (w/v) of 1 of the 8 fucoidans and incubated for 5 days (*n* = 3). Degradation was quantified by full monosaccharide analysis and monosaccharide-specific depletion was calculated as described above (equation (1)). These data—63 community–substrate combinations—were used to validate the model with out-of-sample predictions. To account for variability in monosaccharide composition across polymers, the contribution of each monomer to total degradation was weighted by its relative abundance in the polysaccharide.

To account for potential co-extracted polysaccharides, we performed full monosaccharide profiling using an extended 10 min LC–MS method resolving 21 monosaccharides. Acid hydrolysis, PMP derivatization, internal controls and data processing were performed as described above. Chromatographic separation differed only in the gradient, with an initial isocratic hold at 15% buffer B for 2.0 min, followed by a linear gradient from 15% to 20% buffer B over 5.5 min, a rapid increase to 100% buffer B at 7.5 min, a 1 min wash step at 100% buffer B, and re-equilibration to initial conditions until 9.5 min at a constant flow rate of 0.5 ml min^−1^ and a column temperature of 50 °C. The standard mix comprised fucose, galactose, xylose, mannose, glucuronic acid, glucose, mannuronic acid, guluronic acid, rhamnose, glucosamine, galactosamine, gulose, allose, idose, galacturonic acid, lyxose, ribose, arabinose, iduronic acid, talose and altrose, prepared in matrix-matched conditions across a concentration range of 100 nM to 500 µM. Monosaccharide concentrations were converted to anhydro-corrected masses to approximate polysaccharide yields and sulfate content was inferred from literature-reported weight fractions using the fucoidan-associated monosaccharide pool. This enabled a quantitative mass balance for each fucoidan, yielding estimates of total hydrolysable carbohydrates and unaccounted fractions (Supplementary Table 9).

### Heterologous enzyme expression and purification

The protein-coding sequences of 17 glycoside hydrolases, omitting their native signal peptides^78^, were codon-optimized, synthesized, and cloned into the pET-28a(+) expression vector harbouring an N-terminal 6×His-tag by Twist Bioscience. The resulting plasmids were transformed into BL21(DE3) competent *Escherichia coli* (New England Biolabs) according to the manufacturer’s instructions. Expression strains were cultured in 200 ml of Luria-Bertani (LB) medium supplemented with kanamycin at 37 °C until an OD_600_ of 0.8 was reached. Protein expression was induced by the addition of 0.1 mM isopropyl β-d-1-thiogalactopyranoside (IPTG), followed by incubation at 12 °C for 16 h. Cells were collected by centrifugation at 3,000*g* for 20 min at 4 °C. Cell pellets were lysed using B-PER Bacterial Protein Extraction Reagent (Thermo Fisher Scientific). The proteins were purified from the soluble fraction by immobilized metal affinity chromatography (IMAC) using His GraviTrap TALON columns (Cytiva 29-0005-94). Columns were equilibrated in lysis buffer prior to loading. Nonspecifically bound proteins were removed by sequential washes consisting of two 10 ml washes with IMAC 20 buffer (50 mM Tris-HCl, 500 mM NaCl, 5% glycerol, 20 mM imidazole, pH 8) and two 10 ml washes with IMAC 40 buffer (50 mM Tris-HCl, 500 mM NaCl, 5% glycerol, 40 mM imidazole, pH 8). The His-tagged enzymes were subsequently eluted using 2 ml of IMAC 200 buffer (50 mM Tris-HCl, 500 mM NaCl, 5% glycerol, 200 mM imidazole, pH 8).

Successful expression, purity, and the expected molecular weights of the 17 enzymes were assessed and confirmed by SDS-PAGE followed by Coomassie staining. In total, nine proteins were successfully expressed in the soluble fraction. These purified enzymes were dialysed overnight at 4 °C against a buffer consisting of 50 mM Tris-HCl and 500 mM NaCl (pH 8.0). Final protein concentrations were quantified using the broad-range Qubit Protein BR Assay (Thermo Fisher Scientific). Nine proteins were soluble: V25|GH97_A, V25|GH36, F56|GH39, F56|GH130, F56|GH92_C, F56|GH92_E, F56|GH115_C, V69|GH97_A and V69|GH97_B. Full construct names, accession numbers, construct sequences, molecular weights, signal peptide predictions and solubility are detailed in Supplementary Table 7.

### Enzyme activity assays

All enzyme assays were performed in MBL medium supplemented with 50 mM phosphate buffer (pH 8.0) at 20 °C, using a final enzyme concentration of 5 nM. Initial functional screens were conducted on a panel of pNP-labelled substrate analogues at a concentration of 1 mM, including pNP-α-d-galactopyranoside, pNP-α-d-mannopyranoside, pNP-α-d-xylopyranoside, pNP-β-d-galactopyranoside, pNP-β-d-mannopyranoside and pNP-β-d-xylopyranoside. Enzymatic activity was quantified by monitoring the release of para-nitrophenol at 410 nm using a microplate reader.

Michaelis–Menten kinetics for F56|GH39 and V25|GH36 were determined from initial rates measured across a substrate gradient (0.1–100 mM) using pNP-labelled substrate analogues. Initial velocities were calculated from the linear phase of product formation. Kinetic parameters (*K*_m_, *V*_max_) were estimated by nonlinear least-squares fitting to the Michaelis–Menten equation.

To assess activity on native substrates, enzymes were incubated with 0.2% (w/v) fucoidan from *F. vesiculosus* and 0.1% (w/v) residual fucoidan recovered from cultures of the primary degraders V4, V25 and V69. Reactions were incubated for 24 h and released monosaccharides were quantified following PMP derivatization by LC–MS.

### Distribution of fucoidan-degrading isolates

To determine the distribution of fucoidan-degrading isolates across ocean environments, we assessed their detection in global rRNA gene– and metagenome-based databases. First, 16S rRNA genes were extracted from isolate genomes using pyBarrnap v0.5.1 (evalue = 10^−6^, lencutoff = 0.8, reject = 0.25) and compared to the MicrobeAtlas database^79^, with matches defined as ≥99% sequence identity. In parallel, isolate genomes were compared to species in the mOTUs database^55^—a global, species-resolved collection of genomes from isolates and metagenomes—using the classify function of the mOTUs profiler, which aligns ten single-copy marker genes to representative genomes.

As the mOTUs database contains relatively few macroalgae-associated microbiome samples, we additionally compared isolates to two recent macroalgae-associated datasets. The first was an amplicon sequence variant (ASV) dataset from an annual sampling campaign of macroalgal thalli along the Roscoff coastline^80^; ASVs were aligned to full-length isolate 16S rRNA genes and retained if ≥99% identical. The second comprised isolate genomes and metagenome-assembled genomes from epiphytic microbiomes of macroalgae in a coastal region of China^81^; genomes were compared using species-level clustering with dRep v3.5.0 (−comp 50 −con 10 −sa 0.95 −nc 0.3), using a 95% ANI threshold for species-level assignment. Following the comparisons of isolates to these reference databases and recently published datasets, we extracted and combined the information on where each of the matched references has been detected from the respective resources and visualized global distributions in R v4.3.3 using ggplot2, rnaturalearth and sf packages.

### Diversity and dynamics of fucoidan degrader and exploiter species across the global oceans

To investigate the distribution and dynamics of fucoidan degraders across the global ocean and assess the potential ecological relevance of synergistic degradation, we analysed species in the mOTUs database. Species-representative genomes were retrieved and restricted to those recovered from isolates or ocean metagenomes. For initial screening, representative genomes were annotated against dbCAN v14^73^ using HMMsearch^72^ (horizontal coverage >0.25; e-value < 1 × 10^−10^) and genomes encoding at least one fucoidanase GH families were retained (*n* = 3,692). These candidates were then subjected to a more comprehensive annotation workflow comprising Diamond^74^ (blastp mode) searches against CAZyDB^82^ and SulfAtlas^75^, and HMMsearch against KEGG^83^ and PFAM. Degrader species (mOTUs) were defined as those encoding at least five fucoidan-targeting GH and sulfatase families. For each degrader species, fucoidan degradation capacity was estimated as the number of fucoidanase genes per genome and fucoidan-degrading PULs were annotated as described in ‘Identification of fucoidan PULs’.

To provide ecological context, the distribution of degrader species was profiled across >11,000 ocean metagenomes using the profile function of the mOTUs profiler. Samples containing at least one degrader mOTUs were retained (*n* = 12,347). For each sample, we calculated the species diversity of detected degraders as well as their relative abundance, determined as the proportion of genomes they represented within the community.

To assess the potential prevalence of synergistic degradation across ocean environments, we analysed the per-sample heterogeneity in fucoidan degradation capacity of co-occurring degraders using two complementary approaches. First, we examined the composition of degrader-encoded fucoidan-targeting PULs in terms of enzymes repertoires targeting the sulfated fucose backbone versus targeting rare-sugar monomers. The species were subsequently grouped into quartiles based on the proportion of fucose-targeting enzymes in their PULs. In each sample, we subsequently defined potential synergistic degradation as the co-occurrence of a top and bottom quartile degrader species—that is, co-occurrence of species with fucose- and rare-sugar monomer specialized PULs. To complement this, we also assessed the median and standard deviation of the fucoidanase gene content of co-occurring degraders in each sample.

### Reporting summary

Further information on research design is available in the Nature Portfolio Reporting Summary linked to this article.

## Online content

Any methods, additional references, Nature Portfolio reporting summaries, source data, extended data, supplementary information, acknowledgements, peer review information; details of author contributions and competing interests; and statements of data and code availability are available at https://doi.org/10.1038/s41586-026-10980-z.

## Supplementary information

Supplementary Information Supplementary Notes 1–4, Supplementary Figs. 1–9, legends for Supplementary Tables 1–9 (tables supplied separately) and gel source data.

Reporting Summary

Supplementary Table 1

Supplementary Table 2

Supplementary Table 3

Supplementary Table 4

Supplementary Table 5

Supplementary Table 6

Supplementary Table 7

Supplementary Table 8

Supplementary Table 9

Peer Review File

## Source data

Source Data Figs. 1, 2, 3

## Data Availability

All data supporting the findings of this study are available within the paper and its Supplementary Information. Raw LC–MS data are available via Panorama Public at https://doi.org/10.6069/r2z3-7b10. Genome assemblies and associated sequencing data have been deposited in the NCBI Sequence Read Archive under accession number PRJNA996876. Accession numbers for all genomes used in this study are listed in Supplementary Table 2. All data underlying the analysis of global ocean metagenomes, including fucoidan degrader annotations, PUL sequences, genome summaries and community profiles, are publicly available on Zenodo (https://zenodo.org/records/19591477 (ref. ^84^)) and https://github.com/tpriest0/Global_ocean_microbial_fucoidan_degrader_analysis. Publicly available databases used in this study were: the CAZy database (http://www.cazy.org; accessed September 2022 and March 2026), dbCAN v10 and v14 (https://bcb.unl.edu/dbCAN2/), SulfAtlas v1.2 (https://sulfatlas.sb-roscoff.fr/), Pfam (https://www.ebi.ac.uk/interpro/), MetaCyc (https://metacyc.org/), KEGG (https://www.genome.jp/kegg/), GTDB release 202 (https://gtdb.ecogenomic.org/), the mOTUs database (https://motu-tool.org/) and the MicrobeAtlas database (https://microbeatlas.org/). No data in this study are subject to restricted or controlled access. Source data are provided with this paper.

## Code availability

A reproducible workflow for calculating degradation from LC–MS data, along with code for model implementation and validation, is available at: https://github.com/EnvSysMicroLAB/Sichert2025_Fucoidan. Code for the analysis isolate distribution and metagenome analysis is available at: https://github.com/tpriest0/Global_ocean_microbial_fucoidan_degrader_analysis.
