# Supplementary material for: Synergistic degradation of fucoidans in the ocean
Source: Nature. 2026 Aug 26;658(8136):682–9. doi: 10.1038/s41586-026-10980-z (PMC13668718; doi:10.1038/s41586-026-10980-z)
Supplement: Supplementary file 1 [file 41586_2026_10980_MOESM12_ESM.pdf]

nature portfolio

Peer Review File

Synergistic degradation of fucoidans in the ocean

Corresponding Author: Professor Uwe Sauer

Any redactions in this file are there to maintain patient confidentiality, the confidentiality of unpublished data, or to remove third-party material.

This file contains all reviewer reports in order by version, followed by all author rebuttals in order by version.

Version 0:

Reviewer comments:

Referee #1

(Remarks to the Author)
The manuscript by Sichert et al investigates the degradation of fucoidans, complex polysaccharides from brown algae and diatoms that are generally resistant to microbial breakdown. Using seawater enrichments, metagenomics, and cultured isolates, the authors demonstrate the ability of MAGs and isolates to degrade fucoidans, and highlight synergistic interactions (division of labor) among different taxa. The study combines genomics, culturing, chemical quantification, and modeling in a well-written and methodologically diverse presentation.nning genomics, culturing, chemical quantification of monomers and modeling.

Major Comments
1) The central novelty is the division of labor in fucoidan degradation. While single bacterial degradation of fucoidans is already well established (including isolates described here), the cooperative aspect stands out. However, division of labor in marine bacteria has previously been proposed as a driver of complex algal polysaccharide breakdown during blooms (e.g., Teeling et al., Science 2012, 10.1126/science.1218344). The manuscript would benefit from positioning the findings more clearly in this existing context.
2) The ecological framework is underdeveloped. It remains unclear whether this division of labor occurs in natural environments. For example: Do these bacteria form a significant fraction of diatom or brown algal microbiomes? Do they co-occur around hosts that produce fucoidans?
How would their fucoidans degradation activity change when competing substrates (e.g., simple sugars, amino acids) are available, as is typical near live hosts?

Specific Comments
The focus is largely on monomers. What evidence supports uptake of oligosaccharides or larger fragments? Are there genomic features (e.g., receptors, transporters) that point to this? Clarifying the role of intermediates would strengthen the interpretation.

Supplementary Table 1 (media used) is confusing: media are not labeled by name, and compositions are not linked to experimental use. This should be clarified to make Methods more interpretable.

Figure 1a: the sugar linkage/isomer diagram is difficult to interpret. Is this a representative structure or a schematic of all possible linkages? More explanation would help, especially since enzyme specificity depends on bond context.

Figure 1d: controls for abiotic degradation of fucoidans appear missing. For LC-MS quantification, further detail is needed on calibration: how external standards were constituted and how matrix effects were controlled.

Line 365: please define the criteria for MAG quality used in Extended Data Fig. b. Without this, the robustness of the heat map cannot be assessed.

(Remarks on code availability)

Referee #2

(Remarks to the Author)
This study isolates bacteria that can grow on fucoidan, a key complex carbohydrate made by seaweed and diatoms. After isolating a number of species that can grow on fucoidan, the authors then ask how degradation of fucoidan is affected when there are cocultures of two or more species. This reveals that adding additional species can often increase the degradation. They use a measure of degradation when species are in monoculture vs coculture to show that there can be a form of synergism in degradation and, from there, argue that fucoidan degradation rests upon frequent cooperation and a division of labor. Overall, I found the study and data to be informative and interesting but I was not confident that there is a meaningful division of labor or cooperation based on these data. To explain why, I will outline two hypothetical scenarios for the breakdown of a complex molecule, one which is cooperation and a division of labor in a meaningful sense, one which is not. I do this as I think it is important for the authors to clearly demonstrate which one best represents their study system. It may be they can do this with data they already have in hand, but I think it may require additional data and analyses to distinguish the two scenarios.

Case 1: the cooperative division of labor. As a simplified version of fucoidan, imagine a complex molecule made of two monomers A and B, where A is attached to other As to form a backbone, and B is a side moiety that is attached as a single monomer to some of the A monomers on the backbone. Now take two species, i and j, where i can digest the A-A links (and uses A as a nutrient) and j can digest the A-B links (and consumes B). However, because of the way that that two monomers arrange, it is hard for i to digest A-A when B is attached to one of the As. In addition, when A is in a long chain, it is hard for species j to remove B. As a result, when species i cuts some of the A-A links, this allows j to remove the Bs better, which means it can now gain nutrients more easily. In addition, the removal of the Bs helps A to digest the A-A chain and gain A monomers to feed it. The details are not so important for the point but what is important is that both species here help the other to perform their digestion and obtain nutrients. So, this can be argued to be a meaningful division of labor, where both species benefit from the full breakdown of fucoidan, and it also looks like a cooperative interaction (so long that both species also increase in abundance due to the presence of the other strain).

Case 2: no cooperation, no division of labor. Take the same hypothetical compound and two species. However, this time while species i helps j to perform its digestion, species j has no impact on species i. Firstly, this is a commensalism and not cooperation but it is also not a meaningful division of labor because species i has no functional interest or benefit in the degradation by species j and they are not dividing labor to achieve any end goal. Species i is doing what it always does and only the second species benefits from the interaction. If I understand it correctly, the synergism measure in the manuscript would identify this case (as well as the last one) as synergism, cooperation and a division of labor. But the two cases are quite different because in this last case, the goal of complete degradation of the complex molecule only exists in the mind of the experimenter, it is not something that the bacteria have an ecological or evolutionary shared interest in achieving. Distinguishing between these two scenarios does not seem possible based on the current data, unless I am missing something. For example, there seem to be no measurements of the abundances of individual species in the cocultures to show that both parties benefit from the actions of the other and are increasing in coculture when the interpretation of cooperation and division of labor are made. Do the authors have these and other data to help demonstrate one of the two scenarios? I may have missed it in the manuscript, but I could not find a way to do it on my read through.

To end, I want to make clear that I believe the manuscript and data to be important and interesting whether or not the biology is a formal division of labor/cooperation or not, so I don’t feel the outcome is important in that regard. It is though important to work this out for clarity and linking up to the existing literature.

(Remarks on code availability)
NA

Referee #3

(Remarks to the Author)
Fucoidans are among the most complex marine glycan substrates and could potentially act as a carbon sink in the oceans. So far, little is known about microbial strategies for utilising them. This knowledge gap is addressed by the study of Sichert et al.
Although the importance of the synergistic effects of microbial communities in their natural habitats has frequently been described, this is the first study to investigate such an effect in relation to the marine polysaccharide fucoidan. The study is based on a systematic approach that enabled the authors to isolate and determine selected bacterial strains involved in fucoidan metabolism. While no new mechanistic details of metabolism have been revealed, this study clearly demonstrates the collaborative efforts of natural microbial communities in fucoidan recycling processes.
This study takes a unique approach by not only isolating and characterising natural fucoidan-using microbial communities, but also conducting detailed growth experiments involving selected key bacteria and co-cultures, monosaccharide analyses, and mathematical calculations.
The study convincingly reveals that the efficient and complete degradation as well as metabolisation of such complex glycans in nature is only possible through the teamwork of microbial communities and the division of labour. Thus, this study provides valuable insights into how different microbes might cooperate in utilising complex glycans such as fucoidans.

My main concern is that the authors draw highly generalised conclusions based on preliminary sequence analyses of the putative enzyme repertoire of the isolates, although no biochemical enzyme characterisation was performed in this study. Many of the CAZyme functions may simply not be understood well enough to substantiate the inferred enzyme functions in the manuscript. Therefore, categorising the isolates as “degraders”, “exploiters” or “scavengers” may be misleading due to the overgeneralisation of specific enzymatic or metabolic activities of the individual strains, and this categorisation may not be applicable to every isolate investigated.
How precisely can the group of 'backbone-degrading enzymes' be defined? For example, GH141 can be an endoxylanase (https://doi.org/10.1038/s41598-017-11598-y).
Classifying sulfatases is even more difficult. How could the proposed group of sulfatases (S1_15, S1_16, S1_17, S1_22 and S1_25) be defined as being as essential as 'backbone-degrading enzymes' for 'degraders' but not 'exploiters'?
If distinct CAZymes and sulfatases are seen within putative fucoidan PULs, then it could be concluded that these enzymes are involved in disassembly of diverse fucoidan structures. However, what about the other included enzymes whose encoding genes are not clustered in a PUL? How can you be sure that the chosen glycoside families (GH115, GH92, GH130, GH39, GH120, GH36, GH97 and GH117) are indeed involved in fucoidan degradation?

MAG17 and MAG23 dominate ComA enrichment cultures, while MAG25 and MAG26 dominate ComC enrichment cultures. It would be interesting to analyse these MAGs in more detail, deducing and discussing potential reasons for their dominant role in these growth experiments. How does the dominance of these non-isolated strains align with your broader hypothesis regarding labour division in this study, which is based on the experimental analysis of pure, culturable strains but that played a minor role in the fucoidan enrichment cultures (with the exception of isolate V69)?

In general, the figures in this study look good, but they are difficult to read and evaluate. In many cases, the legend does not provide sufficient explanation of the type of data shown and its source. Most of the legends are not self-explanatory.

I have the same problem with the Supplementary Tables, which are also difficult to understand. For example, lines 514–516 in the manuscript state that 'Throughout this study, we generated five distinct datasets, with monosaccharide concentrations and corresponding statistical analyses reported in Supplementary Table 5'. However, the raw data are not given in the table. It is also unclear what is shown in the different sheets of the table. The legend is not self-explanatory.
I recommend including relevant raw and processed data in the Supplementary Tables and revealing how the individual data sets were processed, including statistical analyses, with detailed, self-explanatory legends.
In general, add and explain the source and the processing of all data sets which are used in the figures of this study in supplementary tables (see also below).

Further comments:
Figure 1:
How accurate are the enzyme affiliations to specific substrates as shown in Fig. 1C, including exo- and endo-activities? Could you please provide the enzyme annotation and classification data for each investigated genome and MAG in a supplementary table? Without detailed functional characterisations, how can you be sure that all proposed enzymes are indeed involved in fucoidan degradation processes?
How many biological replicates were used for the data shown in Fig. 1D? If biological replicates were used and mean values are shown, what was the range of fluctuation in the monosaccharide values?
For Fig. 1E, how was the correlation between the relative abundance of 'backbone-degrading enzymes' and the proportion of fucose degradation performed?

Figure 2:
In general, how can you discriminate between real synergistic metabolic interactions on one hand and potential competitive growth conditions due to vitamin or other nutrient relevant activities on the other? What about the secondary metabolism of the individual strains investigated? Can you exclude that such activities influenced the growth activities of the co-cultures and thus the enzymatic-based fucoidan degradation processes?
Fig. 2a, what do you define as „Verruco“? Please specify the 87 pairwise co-cultures of Verrucomicrobiota strains. Where are these raw data shown?
Fig. 2C is difficult to evaluate. Please give more details.
What is exactly shown in Fig. 2D, are these averaged endpoint values of biological replicates? How much did the values of individual experiments vary depending on the length of cultivation? How variable were the values in the individual biological replicates? Have you been able to determine the fragmentation of all fucoidan fragments and the consumption of all released monosaccharides in the “total degradation” value shown?
How was the “residual substrate fraction” in Fig. 2E determined?

Figure 3:
Where are the raw and processed data for Figure 3A? Is the 'total degradation' value of 97.1%, for example, an averaged value? If so, how diverse were the data sets of the individual biological replicates? Which community reached this high fucoidan degradation value? Please specify the dataset for Fig. 3a.
Which communities were selected to generate the data shown in Fig. 3d? Where can these data be found?

Extended Data Figure 1 to 10
It is necessary to provide all the required data sets, together with a thorough description of the data processing to be able to evaluate the figures that are shown.

Check throughout the text the writing style of e.g. “minutes” or “min”, “days” or “d”, “ml” or “mL”, space before “°” etc.

Line 64: delete „of“
Line 85-86: Do you indeed affiliate the previously characterized Lentimonas sp. CC4 (V4)3 strain to the “scavenger”-group? This is in contradiction to the previously published paper of Sichert et al. (https://doi.org/10.1038/s41564-020-0720-2) which reported that this isolate posseses 284 putative fucoidanases and is able to grow on different fucoidan substrates.
Lines 103-108: In lines 92 to 94 of the previous chapter, you mentioned that within the detected PUL regions „canonical “backbone” enzymes targeting sulfate and fucose co-localized with putative “debranching” enzymes belonging to 44 CAZyme families, some of which act on rare sugars.“. Why do you speculate here in lines 103 to 108 on “varying levels of genomic specialization for monomer degradation”? Do you really mean here enzymes which degrade monosaccharides or do you rather mean “debranching” enzymes which you also refer to in the following sentence?
Lines 110-111: You state that “the rare-sugar specialists F12, F40, and F94 lacked endo-acting fucoidanases, which are required to cleave the main chain.” How do you know this? Is your preliminary sequence analysis approach so strict and successful that you can indeed exclude the presence if such enzyme activities in these strains?
Lines 130-144: You work with very defined % values for the “monomer” degradation capacity of each investigated isolate. Is this really a suitable defined value or rather a snapshot and could vary depending on the culture time or growth conditions for each investigated bacterial strain?
Lines 183-184: What do you mean by “demonstrating that synergism can occur independently of direct cell–cell contact or the exchange of signalling molecules.”? It is obvious that the exometabolome in concert with extracellular enzyme activities might be crucial in the postulated division of labour between individual strains in the co-culture communities.
Line 317/326: “Fucus vesiculosus” in italics.
Line 341-342: How could you use such an exact cell number for plating experiments?
Line 432: Here you speak about 29 isolates, in Line 350 about “28 unique bacterial strains”?
Line 439: Add “and were cultivated…”.
Line 451: “for 24 h at 100 °C…”. Check additional missing spaces throughout the text.
Line 441-442: In Line 438 it is mentioned that growth experiments were performed for 5 days. How could you ensure for all investigated isolates that the cell-free supernatants always were harvested at the early stationary phase although different strains revealed different growth rates?
Line 500-503: Please check the sentence, a verb is missing.
Line 546-550: Please indicate in the text that you used an indirect biomass assay. Have you been able to correlate the determined DNA concentration and the real dry biomass in your growth experiments?
Please enlarge the font of a, b, c, d in the Extended Data Fig. 1.
Please describe the exact source of the given MAGs in Supplementary Table 2. Maybe I have overlooked this information, but where have the genome sequences of the 29 isolates and the deduced MAG sequences been deposited? Please add this information if not given. Please explain the difference between the different enrichment cultures ComA-C.

(Remarks on code availability)

Referee #4

(Remarks to the Author)
RE: Review correspondence for Manuscript NY-610A-SN&MTS

Dear Editor:

The manuscript, entitled: “A division of labor controls the degradation of fucoidans in the ocean” by Sauer et al, provides insights into how communities collaborate to metabolize the heterogeneous brown alga polysaccharide, fucoidan. Fucoidan has long been realized to be chemically complex, a feature which is believed to contribute to its recalcitrance and persistence in the environment. Descriptions of carbohydrate active enzymes (CAZymes) involved in dismantling the polysaccharide are limited in the literature. The authors have taken an ecological approach to identify guilds of marine bacteria that exclusively consume either the sidechains or backbone of fucoidans, and determined that these guilds partner together to perform more complete degradation of fucoidan. These relationships appear to be conserved independently of the fucoidan source and chemistry (although the structure of these fucoidans were not characterized) and maintain a high correlation between communities. Although an exciting concept, it was reminiscent of what has been described for other syntrophic relationships. Surprisingly, an in-depth structural characterization of the commercially sourced polysaccharides (e.g., 1D and 2D 1H NMR, glycome profiling, linkage analysis, oligosaccharide chromatography with characterized standards) was not conducted in the study, and no new structures of fucoidans were described. Relying on commercial sources that are crudely characterized limits the reach of their conclusions. In addition, the authors did not provide new mechanistic insights (families, catalytic mechanisms, or structures) into how enzymes operate on the different substrate groupings. Without these or other insights to support mechanisms driving the foraging behaviors, it is difficult to justify acceptance in a journal as prestigious as Nature. More details on these topics are provided below.

Fucoidan Structure
Based upon the title and abstract, the expectation for this study was that there would be systematic characterization of fucoidan structure demonstrating firstly, the fine chemistry of the substrates, and secondly, how this structure systematically changes based upon encoded enzyme activities. Based upon the abstract (Line 23-24): “Despite variation in fucoidan structure across different types of algae, the metabolic functions of degraders remained conserved…”. I was expecting a

detailed analysis on the variation in fucoidan structure. Also, it was not clear where the schematic structure shown in Fig 1A was derived from. Some linkages are shown, some are unknown. What method was used to derive this structure? Knowing the polysaccharide fine-chemistry enables the substrate to be partnered with PUL CAZyme specificity. There is precedent for in the CAZyme field using multiple complex polysaccharides (RG-II (10.1038/nature21725), yeast alpha-mannan (10.1038/nature13995), xyloglucan (10.1038/nature12907), pectin (10.1038/s41564-017-0079-1), mucin (10.1038/s41564-024-01911-7), etc) . Of note, three of these papers were published in Nature.
The authors use language such as “side-branches” and “rare monomers”, which add intrigue, but provide little fundamental insights into the actual substrate structure. For example, a side-branch could mean a side chain (oligosaccharide) or branching point. More relevant description would be the monosaccharide, linkage, and degree of polymerization of the side change. Also, “rare” in this case implies it is not common, but based upon the explanation, this seems to mean less abundant than the sulfate fucosyl residues in the backbone. “Rare” is an ambiguous term, and one could argue that even it present at low amounts (<5%) of the total composition, if the polysaccharide is abundant, the rare monomers would become an abundant nutrient. Rare monosaccharides usually describe chemically distinguished or biological limited monosaccharides, neither of which is applicable here. Also, it is widely recognized problem that polysaccharide purity can chemistry can vary between sources and preparations.
For these reasons, I believe a comprehensive glycomics analysis of fucoidan structure would strengthen the claims of this study. Relying on descriptions of commercial products and monosaccharide analysis does not account for biological and preparative variation, and importantly limits the ability to draw valuable, detailed conclusions on side chain chemistry, branching density, linkage, sulfation patterns, and other structural features.

Guilds
Groups are divided into three ecological guilds: Degraders, exploiters, and scavengers (Line 77-82), which seems based on earlier nomenclature presented in D’Souza et al. (10.1073/pnas.2305198120 and references therein) from their study on alginate. These roles seem analogous to other defining labels used, such as primary degraders, keystone species, etc and so forth described for other systems. Degraders are defined as microorganisms that contain sulfatases and fucosidases. It would seem that primary degraders would need access to the backbone, and in this capacity, inclusion of sulfatases makes complete sense. Fucosidases, however, require the presentation of terminal fucose which can be abundent in oligosaccharides or side chains, but need to be generated by fucanases (endo-acting enzymes) in polysaccharides. Worth mentioning here is that enzyme nomenclature could be more precise and there is overlapping use of fucosidase, fucanase, and “fucoidanase”. See line 144. Fucoidanase has been given the EC number: 3.2.1.211 (formally 3.2.1.44; doi.org/10.3389/fmars.2023.1129982), and defined as a poly[(1->3)-alpha-L-fucoside-2/4-sulfate] glycanohydrolase. This reference activity does not seem to be exclusively assigned to the activities mentioned in the manuscript. It would be helpful for the more general reader if the enzyme activities could be defined and used consistently.
On Line 111 the authors introduce “generalists”. Doesn’t this weaken the hypothesis that bacteria are clustered into functional guilds? The concept of the generalist should be introduced earlier as another functional grouping. Lines 140-146 describe the observation that enzyme copy number and diversity do not necessary correlate with fucoidan degradation. This was a very intriguing result, and the authors use it to support their hypothesis about the division of labor being a requirement for fucoidan degradation. Could it also be explained by substrate heterogeneity and availability in Nature (i.e., Chemically distinct fucoidan from different sources)?

Polysaccharide Utilization Loci
PUL description (Line 91-92). Extended Data Fig. 2A appears to be a relatedness chart of enzymes belonging to families associated with fucoidan degradation. This is not the conventional way to depict PULs in the literature, which are contiguous regions of genes active on a common substrate, under co-regulation, usually contain a TonB-dependent transport system (SusC/D-like proteins). PULs also typically contain unknown proteins or enzymes from other families not commonly associated with a substrate. Representing the complete set of genes in the figure is commonly done to inform enzyme discovery and is of interest to CAZymologists. If the authors do not wish to disclose this information, it does not seem prudent to describe these gene collections as “PULs”. In addition, displaying PUL architectures is useful for visualizing syntenic relationships between microbes. It would be very interesting to see the synteny or relatedness between genes that clustered on a plasmid (G88) and genomically (F56) (Lines 97-98).

Enzyme Classification
Classification of CAZymes (Lines 395-412). The authors have selected a low resolution approach to defining enzyme functions (dbCAN family assignment). Many of the families mentioned are polyspecific, and therefore, a more detailed analysis of sequence (e.g., dbCAN subfamily assignment, SACCHARIS) or structural motif relatedness (e.g. CUPP, SSNpipe) would be helpful. For example, the authors indicate GH92 and GH130 are fucoidan-associated GH families due a predicted mannosidase activities; however, these families encode alpha- and beta- activities, respectively. Are the authors suggesting that fucoidan contains alpha- and beta-linked mannose? Also, it is not clear what is meant by “debranching”. Typically, debranching refers to an enzyme that cleaves an oligosaccharide at a branch point and removes it from the main chain (e.g., starch pullulanase). It is not clear if the enzymes discussed in this study are predicted to be exo-acting, i.e. systematically trimming monosaccharides from the non-reducing end to the backbone, cleave internally along the sequence of the branched oligosaccharide releasing small oligosaccharides (e.g. endo-glycosidase), or specifically cleave the branch point, releasing the entire oligosaccharide? If a combination of the above, perhaps referring to these as side-chain degrading enzymes would clarify this for the reader?
Lastly, many enzymes were identified, assigned to PULs, and putative activities assumed; however, there was no biochemical characterization of enzymes conducted in this study. With the discovery of so many putative CAZymes with functions in dismantling the side chains and backbone of fucoidan, defining properties of the degrader and scavenger roles, structure-function characterization of representative enzymes is warranted.

(Remarks on code availability)

Referee #5

(Remarks to the Author)
I co-reviewed this manuscript with one of the reviewers who provided the listed reports.

(Remarks on code availability)

Version 1:

Reviewer comments:

Referee #2

(Remarks to the Author)
The authors have now added data to classify the ecological interactions among the strains. These data are very helpful and informative and show that the great majority of interactions between the strains are either negative or, when there is a positive effect, they are either commensal or parasitism. There is one example of mutualism in what looks like around 20 interactions that have at least one positive edge(Fig 2b).

To have everything here, I will paste in here to two scenarios that the previous version of the manuscript left open:

“Case 1: the cooperative division of labor. As a simplified version of fucoidan, imagine a complex molecule made of two monomers A and B, where A is attached to other As to form a backbone, and B is a side moiety that is attached as a single monomer to some of the A monomers on the backbone. Now take two species, i and j, where i can digest the A-A links (and uses A as a nutrient) and j can digest the A-B links (and consumes B). However, because of the way that that two monomers arrange, it is hard for i to digest A-A when B is attached to one of the As. In addition, when A is in a long chain, it is hard for species j to remove B. As a result, when species i cuts some of the A-A links, this allows j to remove the Bs better, which means it can now gain
nutrients more easily. In addition, the removal of the Bs helps A to digest the A-A chain and gain A monomers to feed it. The details are not so important for the point but what is important is that both species here help the other to perform their digestion and obtain nutrients. So, this can be argued to be a meaningful division of labor, where both species benefit from the full breakdown of fucoidan, and it also looks like a cooperative interaction (so long that both species also increase in abundance due to the presence of the other strain).

Case 2: no cooperation, no division of labor. Take the same hypothetical compound and two species. However, this time while species i helps j to perform its digestion, species j has no impact on species i. Firstly, this is a commensalism and not cooperation but it is also not a meaningful division of labor because species i has no functional interest or benefit in the degradation by species j and they are not dividing labor to achieve any end goal. Species i is doing what it always does and only the second species benefits from the interaction. If I understand it correctly, the synergism measure in the manuscript would identify this case (as well as the last one) as synergism, cooperation and a division of labor. But the two cases are quite different because in this last case, the goal of complete degradation of the complex molecule only exists in the mind of the experimenter, it is not something that the bacteria have an ecological or evolutionary shared interest in achieving.”

As the authors also state in their response, the new data support the second scenario i.e. there is no cooperation and no true division of labor, beyond that perceived by an experimenter who choses fucoidan degradation as a perceived goal. The reason I am repeating myself above is that, despite having removed the term from the title, the authors are still presenting things as a division of labor throughout and are, therefore, still implying to many that the bacterial species are working cooperatively to achieve a goal.

For example, the results section ends with the concluding sentence: “Together, these findings suggest that functionally specialized and complementary fucoidan degraders frequently co-occur in the ocean, supporting synergistic interactions and division of labour as an ecologically relevant strategy for the degradation of complex marine fucoidans. “

I believe that such statements will be confusing to readers and I am sure many people will miss the one figure panel (2b) and brief text that states that the positive effects are actually all tied up in commensal or parasitic relationships.

If the authors wish to discuss the division of labor, for clarity, I would suggest i) removing the use of the term division of labor from the main body of the text and ii) adding a paragraph to the discussion that cover the ecological and evolutionary bases for the degradation i.e. the authors find evidence that it can emerge spontaneously and robustly from the evolutionary self-interest of each strain, without the need for coordination and cooperation. This finding is itself an important result for the field and one that helps to explain the evolutionary and ecological stability of this fascinating biology, so I would hope including this discussion will help raise the impact and clarity of the paper. Moreover, to bring in the division of labor, this section can also discuss that in one case it appears that there is an example of two species working together and so, in a small number of cases, there may well be a division of labor and cooperation too in these systems.

For the abstract, the use of division of labor could be in a sentence like “Most degradation is driven by negative or commensal relationships among species but in one case out of X we find evidence for a cooperative division of labor”, but otherwise the term can be replaced with ‘interaction’ or something similar.

(Remarks on code availability)

Referee #3

(Remarks to the Author)
The authors have satisfactorily addressed all of my points of criticism. In my view, the statistical analysis of the data—wherever possible in the experiments—has also been conducted appropriately.

(Remarks on code availability)

Referee #4

(Remarks to the Author)
Reviewer 4
The authors have done an excellent job of addressing my previous comments. Below are some final thoughts with some minor corrections suggested to further improve the manuscript.

R4.1 the justification for previous characterization of Fucus vesiculosus fucoidan is sufficient, and the authors have addressed the issue of contamination in R4.2, identifying the presence of laminarian. The monosaccharide data (Fig. 3d and E Data Fig. 10) has strengthened the study.
PULs. The inclusion of Supplementary Fig.8 is welcomed and strengthens the study. I would recommend adopting the colour scheme introduced by Martens et al (2008) 10.1016/j.chom.2008.09.007 for consistency. Similarly, the sugar symbols in Figure 1A (and throughout) should adhere to the Symbol Nomenclature for Glycans (SNFG) system: https://www.ncbi.nlm.nih.gov/glycans/snfg.html.

The addition of phylogenetic analysis of CAZymes for prediction of specificities, inclusion of trees (Supplementary Fig. 6&7), and preliminary enzyme kinetics provide support the authors conclusions. Biochemistry of predicted enzymes was lacking previously, and has strengthened the study. For example, the demonstration of GH92 activity on fucoidan, confirms the presence of alpha-mannose in fucoidans. The inactive GH130 also is consistent with its expected activity and the absence of beta-mannose linkages. The selection of pH8 for GH assays was surprisingly high. Did the authors conduct pH optima to determine this condition?

Line 136-7: Amount of free fucoidan appears inversely proportional to free fucose in V4 (Ext. Data Fig 5). There is a similar effect for free xylose in V25 and V69. I am not sure if the authors fully address this observation. I am curious if the accumulation may be related to transport efficiencies as monosaccharides (generated by hydrolytic processing) are not optimal substrates for SusC/D transport. Is there a potential relationship here? V4 seems to be lacking the fucose permease, TonB-dependent transporter, and other transport systems.

Reviewer 5
Apart from the proper formatting for D/L enantiomeric configuration (e.g., Line 523-4; italicised and large capitals, Line540), all my other concerns have been addressed.

(Remarks on code availability)

Referee #5

(Remarks to the Author)
I co-reviewed this manuscript with one of the reviewers who provided the listed reports.

(Remarks on code availability)

Version 2:

Reviewer comments:

Referee #2

(Remarks to the Author)
The authors have responded to my comments and clarified the issues I had in the last version. I congratulate them on an excellent study.

(Remarks on code availability)

Open Access This Peer Review File is licensed under a Creative Commons Attribution 4.0 International License, which permits use, sharing, adaptation, distribution and reproduction in any medium or format, as long as you give appropriate credit to the original author(s) and the source, provide a link to the Creative Commons license, and indicate if changes were made.
In cases where reviewers are anonymous, credit should be given to 'Anonymous Referee' and the source.
The images or other third party material in this Peer Review File are included in the article's Creative Commons license, unless indicated otherwise in a credit line to the material. If material is not included in the article's Creative Commons license and your intended use is not permitted by statutory regulation or exceeds the permitted use, you will need to obtain permission directly from the copyright holder.
To view a copy of this license, visit https://creativecommons.org/licenses/by/4.0/

Referee #1: marine biogeochemistry

The manuscript by Sichert et al investigates the degradation of fucoidans, complex polysaccharides from brown algae and diatoms that are generally resistant to microbial breakdown. Using seawater enrichments, metagenomics, and cultured isolates, the authors demonstrate the ability of MAGs and isolates to degrade fucoidans, and highlight synergistic interactions (division of labor) among different taxa. The study combines genomics, culturing, chemical quantification, and modeling in a well-written and methodologically diverse presentation.nning genomics, culturing, chemical quantification of monomers and modeling.

Major Comments

1) The central novelty is the division of labor in fucoidan degradation. While single bacterial degradation of fucoidans is already well established (including isolates described here), the cooperative aspect stands out. However, division of labor in marine bacteria has previously been proposed as a driver of complex algal polysaccharide breakdown during blooms (e.g., Teeling et al., Science 2012, 10.1126/science.1218344). The manuscript would benefit from positioning the findings more clearly in this existing context.

Indeed, prior studies described division of labour in marine bacteria, providing the initial motivation for our bottom-up approach with isolates. However, division of labor in Teeling2012 and similar studies typically refers to the mere co-existence of degraders in the presence of <u>different</u> polysaccharides (resource partitioning) or the exchange of metabolites between degraders and scavengers (cross-feeding). True synergistic interactions among degraders towards increased degradation was not subject in these studies. We now reinforced that our mechanistic and quantitative study resolves synergistic interactions between species towards improved degradation of a single, particularly hard to digest marine polysaccharide.

2) The ecological framework is underdeveloped thyey co-occur around hosts that produce fucoidans? How would their fucoidans degradation activity change when competing substrates (e.g., simple sugars, amino acids) are available, as is typical near live hosts?It remains unclear whether this division of labor occurs in natural environments. For example: Do these bacteria form a significant fraction of diatom or brown algal microbiomes? Do they co-occur around hosts that produce fucoidans? How would their fucoidans degradation activity change when competing substrates (e.g., simple sugars, amino acids) are available, as is typical near live hosts?

Response R1.1. ecology:

We thank the reviewer for these comments, which prompted us to substantially strengthen the ecological framework of the manuscript. In the revised version, we now include an analysis of >10,000 publicly available marine metagenomes, demonstrating the widespread co-occurrence of distinct fucoidan degraders and their potential for synergistic interactions in the global ocean (Fig. 4).

Q1: Co-occurrence in algal microbiomes

16S-based searches against the MicrobeAtlas database show that the strongest rare-sugar degrader (Verruco69) co-occurs with the strongest fucose degrader (Verruco4) in >2,000 samples, providing direct

evidence that functionally complementary degraders from our isolate collection coexist in natural communities (Supplementary Note 2; Supplementary Fig. 2). We also found co-existence via full genome matches in a handful of samples in the mOTUs database. This limited functional resolution using taxonomy based methods motivated our metagenome-scale analysis.

Q2: Effects of competing substrates

We have clarified in the main text that degradation yield—and thus interaction outcomes—is primarily constrained by enzymatic repertoires and remains stable under carbon-limited conditions. Consistent with this, we show (Response R4.2) that additional substrates (e.g., laminarin impurities) are fully consumed under nitrogen-replete conditions and do not alter interaction outcomes (Extended Data Fig. 10). However, when systems shift away from complete carbon utilization (e.g., under nitrogen limitation or excess labile substrates), it remains an open question how alternative resources affect interactions and community dynamics (e.g., DOI:10.1038/s41467-021-22177-5).

Specific Comments

The focus is largely on monomers. What evidence supports uptake of oligosaccharides or larger fragments? Are there genomic features (e.g., receptors, transporters) that point to this? Clarifying the role of intermediates would strengthen the interpretation.

Response R1.2.: transporters

We now included all annotated transporters and receptors within fucoidan PULs (Supplementary Table 3; Supplementary Fig. 3). SusD-like homologs potentially bind and transport oligosaccharides, but are restricted to Flavobacteriia. Fucoidan-specific oligosaccharide transporters have not been functionally characterized, and the structural complexity and the transiently low concentrations of intermediates currently preclude direct detection.

While oligosaccharide uptake may occur, we demonstrate that fucose is the primary intermediate driving interactions. Specifically, we provide direct evidence for monosaccharide intermediates (Extended Data Fig. 5). Time-resolved measurements show extracellular accumulation of free fucose (up to 15 µM in Verruco25 and 150 µM in Verruco4; see Responses R3.7 and R5.1), and fucose permeases encoded within PULs further support its role as a shared intermediate (Supplementary Fig. 8).

Supplementary Table 1 (media used) is confusing: media are not labeled by name, and compositions are not linked to experimental use. This should be clarified to make Methods more interpretable.

We revisited the table and ensured that the experimental use of each medium is listed and described in Materials and Methods line 374ff.

Figure 1a: the sugar linkage/isomer diagram is difficult to interpret. Is this a representative structure or a schematic of all possible linkages? More explanation would help, especially since enzyme specificity depends on bond context.

We clarified the structure of this fucoidan in detail in the main text, figure caption and Supplementary Note 1. We revisited the shown structure and adapted it for clarity.

Also, see our response R4.1.

Figure 1d: controls for abiotic degradation of fucoidans appear missing. For LC-MS quantification, further detail is needed on calibration: how external standards were constituted and how matrix effects were controlled.

Response R1.3.: LCMS

Abiotic controls were included in all growth experiments, measured alongside samples, and used for degradation calculations (Eq. 1), as described in Materials and Methods, lines 592ff. The exact values of these blanks are reported alongside measured samples in Supplementary Table 4.

External calibration standards were prepared in the same matrix as samples (MBL medium with HCl/NaOH), with monosaccharide concentrations defined based on their maximal abundance in *Fucus vesiculosus* fucoidan to ensure appropriate dynamic range, as described in Materials and Methods, lines 547ff.

As described above, matrix effects are minimized by matching standard and sample matrices. Additionally, acid hydrolysis homogenizes complex biological samples and the PMP derivatization is insensitive to salts and highly specific for carbohydrates (Rührmann, 2014). Moreover, samples are effectively ~10,000-fold diluted throughout the entire workflow, resulting in highly homogenous chromatography and ionization conditions, as described in Materials and Methods, lines 537–557.

Line 365: please define the criteria for MAG quality used in Extended Data Fig. b. Without this, the robustness of the heat map cannot be assessed.

We clarified that only medium and high quality MAGs are shown and updated Supplementary Table 2 according to MIMAG standards (Bowers et al., 2017).

Referee #2: microbiome

This study isolates bacteria that can grow on fucoidan, a key complex carbohydrate made by seaweed and diatoms. After isolating a number of species that can grow on fucoidan, the authors then ask how degradation of fucoidan is affected when there are cocultures of two or more species. This reveals that adding additional species can often increase the degradation. They use a measure of degradation when species are in monoculture vs coculture to show that there can be a form of synergism in degradation and, from there, argue that fucoidan degradation rests upon frequent cooperation and a division of labor. Overall, I found the study and data to be informative and interesting but I was not confident that there is a meaningful division of labor or cooperation based on these data. To explain why, I will outline two hypothetical scenarios for the breakdown of a complex molecule, one which is cooperation and a division of labor in a meaningful sense, one which is not. I do this as I think it is important for the authors to clearly demonstrate which one best represents their study system. It may be they can do this with data they already have in hand, but I think it may require additional data and analyses to distinguish the two scenarios.

Case 1: the cooperative division of labor. As a simplified version of fucoidan, imagine a complex molecule made of two monomers A and B, where A is attached to other As to form a backbone, and B is a side moiety that is attached as a single monomer to some of the A monomers on the backbone. Now take two species, i and j, where i can digest the A-A links (and uses A as a nutrient) and j can digest the A-B links (and consumes B). However, because of the way that that two monomers arrange, it is hard for i to digest A-A when B is attached to one of the As. In addition, when A is in a long chain, it is hard for species j to remove B. As a result, when species i cuts some of the A-A links, this allows j to remove the Bs better, which means it can now gain

nutrients more easily. In addition, the removal of the Bs helps A to digest the A-A chain and gain A monomers to feed it. The details are not so important for the point but what is important is that both species here help the other to perform their digestion and obtain nutrients. So, this can be argued to be a meaningful division of labor, where both species benefit from the full breakdown of fucoidan, and it also looks like a cooperative interaction (so long that both species also increase in abundance due to the presence of the other strain).

Case 2: no cooperation, no division of labor. Take the same hypothetical compound and two species. However, this time while species i helps j to perform its digestion, species j has no impact on species i. Firstly, this is a commensalism and not cooperation but it is also not a meaningful division of labor because species i has no functional interest or benefit in the degradation by species j and they are not dividing labor to achieve any end goal. Species i is doing what it always does and only the second species benefits from the interaction. If I understand it correctly, the synergism measure in the manuscript would identify this case (as well as the last one) as synergism, cooperation and a division of labor. But the two cases are quite different because in this last case, the goal of complete degradation of the complex molecule only exists in the mind of the experimenter, it is not something that the bacteria have an ecological or evolutionary shared interest in achieving. Distinguishing between these two scenarios does not seem possible based on the current data, unless I am missing something. For example, there seem to be no measurements of the abundances of individual species in the cocultures to show that both parties benefit from the actions of the other and are increasing in coculture when the interpretation of cooperation and division of labor are made. Do the authors have these and other data to help demonstrate one of the two scenarios? I may have missed it in the manuscript, but I could not find a way to do it on my read through.

To end, I want to make clear that I believe the manuscript and data to be important and interesting whether or not the biology is a formal division of labor/cooperation or not, so I don't feel the outcome is important in that regard. It is though important to work this out for clarity and linking up to the existing literature.

Referee #2 (Remarks on code availability):

NA

We thank the reviewer for highlighting the ecological distinction between a true cooperative division of labor (Case 1) and commensalism (Case 2). To directly test which scenario applies to our system, we performed qPCR to measure the absolute abundances of individual species in mono- versus cocultures and resolve reciprocal fitness benefits.

The new qPCR data revealed:

1. Most interactions are Commensal (Case 2): In the majority of our cocultures, the primary degrader received no reciprocal fitness benefit from the secondary strain. These are commensal interactions, not a true division of labor.

2. Verruco69 and Gamma88 show True Division of Labor (Case 1): We found a distinct exception in the interaction between Verruco69 and Gamma88. Our qPCR data demonstrated clear reciprocal fitness benefits, with both species significantly increasing in abundance when grown together. This mutualism confirms a true, cooperative division of labor.

Changes made to the manuscript:

- We have added the new qPCR abundance data to the results.
- We revised the text to explicitly differentiate interaction types that underlie synergistic degradation outcomes.
- We now strictly reserve the terms "cooperation" and "division of labor" for the Verruco69-Gamma88 pairing, aligning our terminology with established ecological definitions (e.g., Giri et al., 2019).
- We removed the term division of labor from the title.

Referee #3: marine polysaccharides

Fucoidans are among the most complex marine glycan substrates and could potentially act as a carbon sink in the oceans. So far, little is known about microbial strategies for utilising them. This knowledge gap is addressed by the study of Sichert et al. Although the importance of the synergistic effects of microbial communities in their natural habitats has frequently been described, this is the first study to investigate such an effect in relation to the marine polysaccharide fucoidan. The study is based on a systematic approach that enabled the authors to isolate and determine selected bacterial strains involved in fucoidan metabolism. While no new mechanistic details of metabolism have been revealed, this study clearly demonstrates the collaborative efforts of natural microbial communities in fucoidan recycling processes.
This study takes a unique approach by not only isolating and characterising natural fucoidan-using microbial communities, but also conducting detailed growth experiments involving selected key bacteria and co-cultures, monosaccharide analyses, and mathematical calculations. The study convincingly reveals that the efficient and complete degradation as well as metabolisation of such complex glycans in nature is only possible through the teamwork of microbial communities and the division of labour. Thus, this study provides valuable insights into how different microbes might cooperate in utilising complex glycans such as fucoidans.

My main concern is that the authors draw highly generalised conclusions based on preliminary sequence analyses of the putative enzyme repertoire of the isolates, although no biochemical enzyme characterisation was performed in this study. Many of the CAZyme functions may simply not be understood well enough to substantiate the inferred enzyme functions in the manuscript. Therefore, categorising the isolates as “degraders”, “exploiters” or “scavengers” may be misleading due to the overgeneralisation of specific enzymatic or metabolic activities of the individual strains, and this categorisation may not be applicable to every isolate investigated.

Response R3.1. Guilds:
We included additional information confirming the classification of isolates in two ways. A) Growth assays on fucoidan and fucoidan monosaccharides (Supplementary Figure 1) demonstrate that degraders can grow on fucose and fucoidan, exploiters only on fucose, and scavengers on neither. B) LC-MS analysis of all strains (Extended Data Fig. 5d) shows that only degraders degrade fucoidan. The classification based on genomic information alone is in full agreement with these additional physiological validations.

How precisely can the group of 'backbone-degrading enzymes' be defined? For example, GH141 can be an endoxylanase (https://doi.org/10.1038/s41598-017-11598-y). Classifying sulfatases is even more difficult.
Response R3.2. backbone:
We clarified in Main Text and Supplementary Note 3 that these enzymes are classified based on our current understanding of fucoidan degradation, namely:
- Proteomic and transcriptomic studies show that these enzyme families are highly upregulated in response to fucoidan.
- Members of GH107, GH168, GH141, GH29, GH95, S1_17, S1_22, and S1_25 are demonstrated fucoidan active enzymes.
- All members of the GH141 show homology to the newly identified endo-fucoidanse activity (https://pmc.ncbi.nlm.nih.gov/articles/PMC12786026/) which led us to change Main Text & Figure 1C accordingly.

While the increasing biochemical characterization of enzymes might change details about enzyme functions, the main conclusion of genomically specialized degraders was verified with metabolomics.
Note that we generally refined our sequence analysis of CAZymes, see response R4.3 for details.

How could the proposed group of sulfatases (S1_15, S1_16, S1_17, S1_22 and S1_25) be defined as being as essential as 'backbone-degrading enzymes' for 'degraders' but not 'exploiters'?
We agree that classifying sulfatases is inherently challenging, and we did not intend to imply that these sulfatase families are individually essential for fucoidan degradation. As outlined in Response R3.2 and Supplementary Note 3, these S1 families (S1_15, S1_16, S1_17, S1_22 and S1_25) are implicated in fucoidan degradation based on previous biochemical characterizations and omics studies. In our dataset, they are consistently found in degraders but absent from exploiters and scavengers (Extended Data Fig. 1), that do not degrade fucoidan (Extended Data Fig. 5). We therefore interpret these enzymes as associated with the fucoidan-degrading machinery of degraders. We did not define them as universally essential genes.

If distinct CAZymes and sulfatases are seen within putative fucoidan PULs, then it could be concluded that these enzymes are involved in disassembly of diverse fucoidan structures. However, what about the other included enzymes whose encoding genes are not clustered in a PUL?
While fucoidan-targeting CAZymes are often co-localized within PULs, a subset of enzymes is encoded outside these loci. To capture the full repertoire, we extracted all non-PUL enzymes from Supplementary Table 3, which revealed that only strains Verruco25 and Verruco69 encode a substantial number of such genes (Table 1). Because genomic clustering does not necessarily reflect functional relevance and may instead arise from evolutionary history or regulatory organization, we included both PUL-associated and non-PUL enzymes from characterized and putative fucoidanases to avoid bias in enzyme repertoire

comparisons. Notably, two non-PUL enzymes, V25|GH97_A and V69|GH97_A, were experimentally validated as fucoidan-active α-galactosidases.

Table 1: Total number of fucoidanases in the genomes of isolated degraders and number of fucoidanases that are not clustered in PULs.

Strain	Total fucoidanases	Non-PUL fucoidanases
Flavo12	36	0
Verruco25	48	12
Flavo40	67	1
Flavo56	127	1
Verruco69	36	27
Gamma88	13	0
Flavo94	52	4
Verruco4	72	5

How can you be sure that the chosen glycoside families (GH115, GH92, GH130, GH39, GH120, GH36, GH97 and GH117) are indeed involved in fucoidan degradation?

Response R3.3. enzymes:

As originally described, these enzyme families are enriched in organisms that preferentially consume galactose, xylose, glucuronic acid and mannose. We now clarify that these enzymes are exo-acting and predicted to target specific fucoidan-associated monosaccharides (see also response R4.3.). To demonstrate their involvement in fucoidan degradation, we selected 17 enzymes for heterologous expression.

- For six enzymes, we experimentally confirmed the predicted exo-acting activity on fucoidan (Fig. 2h), namely:
    - V25|GH97_A, V69|GH97_A, and V69|GH97_B are exo-acting alpha-galactosidases
    - V25|GH36 is an exo-acting alpha-galactosidases
    - F56|GH39 is an exo-acting alpha-xylosidase
    - F56|GH92_E is exo-acting alpha-mannosidase
- For two of these enzymes, we further determined kinetic parameters using pNP substrate analogues (Extended Data Fig. 7).

Together, these results provide direct experimental evidence that these enzyme families contribute to fucoidan degradation. Due to the structural complexity of fucoidans and the lack of defined oligosaccharide standards, precise linkage specificity cannot currently be resolved.

MAG17 and MAG23 dominate ComA enrichment cultures, while MAG25 and MAG26 dominate ComC enrichment cultures. It would be interesting to analyse these MAGs in more detail, deducing and discussing potential reasons for their dominant role in these growth experiments. How does the dominance of these non-isolated strains align with your broader hypothesis regarding labour division in this study, which is based on the experimental analysis of pure, culturable strains but that played a minor role in the fucoidan enrichment cultures (with the exception of isolate V69)?

Response R3.4. mags

Despite extensive efforts (>800 colonies screened), the dominant MAGs could not be isolated under our cultivation conditions, and the reasons for their lack of culturability remain unclear. However, as shown in Extended Data Fig. 1 and 4, our isolated degraders span the full range of genomic specialization

observed in the enrichment cultures. We therefore expect that the interactions resolved in isolates capture the same functional dynamics present in the enrichment communities.
As you pointed out, MAG23 and MAG25 reached >80% abundance at the final timepoint in the enrichments, indicating high fitness under the applied selection regime. Importantly, these MAGs represent distinct functional strategies: MAG23 is highly similar to isolate Verruco25 and encodes a specialized repertoire (~30 enzymes) primarily targeting fucose, whereas MAG25 encodes a broader repertoire (>100 enzymes), consistent with a more generalist degradation strategy similar to Flavo56. This shows that different strategies can dominate under similar conditions and the incomplete capabilities of MAG23 suggests the synergistic interactions in the enrichments. Due to the stochastic nature of community assembly and the limited number of replicates (n = 3), we could not robustly infer statistically significant co-occurrence patterns between specific degrader types. In response R1.1., we include additional ocean metagenomic data, showing that many different types of degraders co-exist in natural samples.

In general, the figures in this study look good, but they are difficult to read and evaluate. In many cases, the legend does not provide sufficient explanation of the type of data shown and its source. Most of the legends are not self-explanatory.
I have the same problem with the Supplementary Tables, which are also difficult to understand. For example, lines 514–516 in the manuscript state that 'Throughout this study, we generated five distinct datasets, with monosaccharide concentrations and corresponding statistical analyses reported in Supplementary Table 5'. However, the raw data are not given in the table. It is also unclear what is shown in the different sheets of the table. The legend is not self-explanatory.
I recommend including relevant raw and processed data in the Supplementary Tables and revealing how the individual data sets were processed, including statistical analyses, with detailed, self-explanatory legends.
In general, add and explain the source and the processing of all data sets which are used in the figures of this study in supplementary tables (see also below).
We revisited all Figures and Tables and improved according to your recommendations in the revised manuscript. All annotated raw MS data are deposited at Panorama (https://doi.org/10.6069/r2z3-7b10). Data processing is described in detail in the Materials and Methods, including Eq. 1, lines 591–661, and the original script used to convert raw MS data into concentrations is available on GitHub (https://github.com/EnvSysMicroLAB/Sichert2025_Fucoidan). All raw concentrations, inferred degradation values, statistical analysis including p- and q-values are included in Supplementary Table 4. A Source Data Table containing replicate level data is included in the revised manuscript.

Further comments:
Figure 1:
How accurate are the enzyme affiliations to specific substrates as shown in Fig. 1C, including exo- and endo-activities?
See Response R4.3; we have refined our sequence-based annotation approach. Briefly, we now provide confidence scores, EC numbers and inferred activities for each enzyme in Supplementary Table 3, with detailed discussion in the Supplementary Note 3 and Extended Data Fig. 3.
Most enzyme families analysed here exhibit one major activity, which can be assigned to the family level with reasonable confidence. Even for the GH141, which is known to be polyspecific with multiple reported activities, we show homology to newly identified endo-fucoidanse activity (https://pmc.ncbi.nlm.nih.gov/articles/PMC12786026/), re-classified it as endo-acting and changed

Figure 1C accordingly, with full details available in Extended Data Fig. 3 and Supplementary Table 3.

Could you please provide the enzyme annotation and classification data for each investigated genome and MAG in a supplementary table?

The classification of each MAG is provided in Supplementary Table 2 and enzyme annotation in Supplementary Table 3.

Without detailed functional characterisations, how can you be sure that all proposed enzymes are indeed involved in fucoidan degradation processes?

We refined our sequence analysis approach (Response R4.3) and provided detailed biochemical characterization of six novel fucoidan acting enzymes (Response R3.3).

How many biological replicates were used for the data shown in Fig. 1D? If biological replicates were used and mean values are shown, what was the range of fluctuation in the monosaccharide values?

We clarified in figure legend that Fig 1D shows two example chromatograms before and after growth of degrader V69.

Response R3.5. variability

In general, all monomer degradation presented throughout this paper rely on at least three biological independent growth experiments, measured in technical duplicates along with no-cells abiotic controls (see response R1.3.). Our statistical workflow to detect significant degradation (corrected for multiple testing and false discovery rate) is described in Materials and Methods, lines 602ff. Example code can be found on github. Raw concentrations, statistics and inferred degradation are reported in Supplementary Table 4 and Source Data Table.

For the 127 tested biological conditions tested in the 7-member community experiment, the %CVs across 3 biological replicates were:

Fucose: 2.09%

Galactose: 6.98%

GlucuronicAcid: 4.43%

Mannose: 5.71%

Xylose: 2.52%

Total: 2.33%

Rare: 3.44%

For Fig. 1E, how was the correlation between the relative abundance of 'backbone-degrading enzymes' and the proportion of fucose degradation performed?

For each degrader, monomer preference was defined as the fraction of fucose consumption, calculated as fucose degraded (mM) divided by the sum of all measured fucoidan-derived monosaccharides (Fuc / [Fuc + Gal + Xyl + Man + GlcA]) from end-point LC–MS measurements. Genomic investment into fucose backbone degradation was defined as the proportion of fucose and sulfate targeting enzymes (GH29, GH95, GH141, GH107, GH168, S1_15, S1_16, S1_17, S1_22 and S1_25) relative to the total set of annotated fucoidan-degrading enzymes in each genome. The relationship between these variables across strains was evaluated using ordinary least squares regression (Fig. 1E). All underlying measurements, replicate-level data, and regression statistics (slope, intercept, R^2, p-value, n) are provided in the Source Data for Fig. 1E.

Figure 2:

In general, how can you discriminate between real synergistic metabolic interactions on one hand and potential competitive growth conditions due to vitamin or other nutrient relevant activities on the other? What about the secondary metabolism of the individual strains investigated? Can you exclude that such activities influenced the growth activities of the co-cultures and thus the enzymatic-based fucoidan degradation processes?

Response R3.6. cross-feeding:

A common way to discriminate between these scenarios is cross-feeding of culture supernatants (https://www.nature.com/articles/s41564-024-01625-w). By purifying the residual fucoidans of primary degraders, we remove small metabolites such as vitamins and show that secondary degraders degrade it to an extent that perfectly matches the expectation from co-cultures (Fig 2 e,f,g). Hence, we can rule out other mechanisms than enzymatic synergism.

Fig. 2a, what do you define as „Verruco"? Please specify the 87 pairwise co-cultures of Verrucomicrobiota strains. Where are these raw data shown?

We clarified in the main text, figure and legend that Verruco means Verrucomicrobiota. The raw data are shown in Extended Data Figure 6, Supplementary Table 4, 5, and Source Data File.

Fig. 2C is difficult to evaluate. Please give more details.

For now Fig. 2D in the revised manuscript, the y-axis represents the synergism score (σ), calculated as defined in the equation shown in Fig. 2C and Eq. 2 in Materials and Methods, lines 660ff. Briefly, for each strain, the monosaccharide degradation profile in monoculture (Fuc, Gal, Xyl, Man, GlcA) was normalized by z-scoring to obtain a standardized consumption vector. Pairwise similarity between strains was quantified as the correlation between these z-scored vectors. The resulting similarity metric was related to the σ score when respective strains are combined. All underlying vectors, pairwise correlation values, and σ scores, together with the corresponding statistical analysis on a replicate level, are provided in the Source Data for Fig. 2D.

What is exactly shown in Fig. 2D, are these averaged endpoint values of biological replicates?

We clarified in the main text and figure legend that this shows the mean degradation of three biological replicates. Source data are now included.

How much did the values of individual experiments vary depending on the length of cultivation?

Response R3.7. dynamics

We now include time-course measurements of monomer degradation for the eight degraders (Extended Data Fig. 5). Monomer concentrations decrease as OD600 increases, indicating a tight coupling between degradation and growth. Due to the high analytical load of LC–MS time-course measurements, we did not resolve full degradation dynamics for all co-cultures, but relied on end-point measurements when the total degradation yield was saturated (see also response to R1.2).

How variable were the values in the individual biological replicates?

We went through the figures and included error bars of biological replicates whenever appropriate and possible (see also response to R3.5) and replicate level source data is included.

Have you been able to determine the fragmentation of all fucoidan fragments and the consumption of all released monosaccharides in the "total degradation" value shown?

Response R3.8. mass recovery:
Fucoidans are generally fully acid-hydrolyzable, enabling quantitative mass recovery of their constituent monosaccharides. The sum of measured anhydro-corrected fucoidan monosaccharides, together with literature-based sulfate contents, nearly closes the mass balance for high-purity fucoidans. For the main fucoidan from *Fucus vesiculosus*, we achieve a mass recovery of 96.4 ± 2.1% (n = 3). Thus, monomer degradation analysis captures nearly all available substrate carbon. In response to R4.2, we now provide full mass balance calculations for all fucoidans in Supplementary Table 9.

How was the "residual substrate fraction" in Fig. 2E determined?
Our degradation assay explicitly relies on measuring the concentration before growth (input) and after growth (residual). The concentrations of all residuals measured are reported in the Source Data and Supplementary Table 4, which we used to calculate degradation using Equation 1.

Figure 3:
Where are the raw and processed data for Figure 3A? Is the 'total degradation' value of 97.1%, for example, an averaged value? If so, how diverse were the data sets of the individual biological replicates? Which community reached this high fucoidan degradation value? Please specify the dataset for Fig. 3a.
Which communities were selected to generate the data shown in Fig. 3d? Where can these data be found?
We made it more clear now that the requested information can be found in Supplementary Table 4, Source Data File and included Extended Data Figure 8 and 10 that visualized the degradation for each community. We made it clear in the main text that 97.1% is the maximum degradation found in one specific strain combination averaged from three biological replicates.

Extended Data Figure 1 to 10
It is necessary to provide all the required data sets, together with a thorough description of the data processing to be able to evaluate the figures that are shown.
We went through the manuscript again, clarified where the data sets can be found and added details to ED figure 1 to 10 when possible.

Check throughout the text the writing style of e.g. "minutes" or "min", "days" or "d", "ml" or "mL", space before "°" etc.
Done

Line 64: delete „of"
Done

Line 85-86: Do you indeed affiliate the previously characterized Lentimonas sp. CC4 (V4)3 strain to the "scavenger"-group? This is in contradiction to the previously published paper of Sichert et al. (https://doi.org/10.1038/s41564-020-0720-2) which reported that this isolate posseses 284 putative fucoidanases and is able to grow on different fucoidan substrates.
We changed the wording of the main text to clarify that Lentimonas CC4 (V4) is a degrader.

Lines 103-108: In lines 92 to 94 of the previous chapter, you mentioned that within the detected PUL regions „canonical "backbone" enzymes targeting sulfate and fucose co-localized with

putative "debranching" enzymes belonging to 44 CAZyme families, some of which act on rare sugars.". Why do you speculate here in lines 103 to 108 on "varying levels of genomic specialization for monomer degradation"? Do you really mean here enzymes which degrade monosaccharides or do you rather mean "debranching" enzymes which you also refer to in the following sentence?
We clarify in the main text that we mean "debranching" enzymes.

Lines 110-111: You state that "the rare-sugar specialists F12, F40, and F94 lacked endo-acting fucoidanases, which are required to cleave the main chain." How do you know this? Is your preliminary sequence analysis approach so strict and successful that you can indeed exclude the presence if such enzyme activities in these strains?
In our original submission, we did not detect homologs of the canonical endo-acting fucoidanases GH107 and GH168 in these strains, consistent with CAZy annotations (https://www.cazy.org/b36830.html). Following the recent characterization of endo-acting GH141 enzymes (https://pmc.ncbi.nlm.nih.gov/articles/PMC12786026/), we updated Fig. 1c and the main text accordingly. While we cannot exclude the presence of endo-acting activities, their classification as rare-sugar specialists is supported by limited growth and low fucose degradation, consistent with a reduced capacity to cleave the fucoidan backbone.

Lines 130-144: You work with very defined % values for the "monomer" degradation capacity of each investigated isolate. Is this really a suitable defined value or rather a snapshot and could vary depending on the culture time or growth conditions for each investigated bacterial strain?
See Response R3.7 for time-course data and Response R3.5 for variability. In brief, monomer degradation closely follows growth, and the reported % degradation values represent robust end-point measurements. Across biological replicates and conditions, variation was below 5%, indicating that these values are reproducible rather than transient snapshots. Replicate level degradation data for each Figure can be found in the Source Data File.

Lines 183-184: What do you mean by "demonstrating that synergism can occur independently of direct cell–cell contact or the exchange of signalling molecules."? It is obvious that the exometabolome in concert with extracellular enzyme activities might be crucial in the postulated division of labour between individual strains in the co-culture communities.
We clarified that this experiment demonstrates that different degraders don't need to be in direct contact and their interaction depends on the enzymatic complementarity. As discussed in Response R3.6., the degradation in bacterial co-cultures can indirectly be driven by exchange of many different extracellular effector molecules such as vitamins, antibiotics or siderophores.

Line 317/326: "Fucus vesiculosus" in italics.
Done
Line 341-342: How could you use such an exact cell number for plating experiments?
Clarified that a counting chamber and microscope was used.
Line 432: Here you speak about 29 isolates, in Line 350 about "28 unique bacterial strains"?
We clarified in text that this is due to the additional inclusion of Verruco4 as an available control strain.
Line 439: Add "and were cultivated…".
Done
Line 451: "for 24 h at 100 °C…". Check additional missing spaces throughout the text.

Thank you, done

Line 441-442: In Line 438 it is mentioned that growth experiments were performed for 5 days. How could you ensure for all investigated isolates that the cell-free supernatants always were harvested at the early stationary phase although different strains revealed different growth rates?

Note that the relevant section has changed, since we measured the full-time course of all degraders, which shows that degradation yields stabilize over growth (see Response 3.7.). In the original submission, early stationary time points were determined using OD600. In community experiments, we ensured that growth yield saturated by monitoring OD600.

Line 500-503: Please check the sentence, a verb is missing.

Done

Line 546-550: Please indicate in the text that you used an indirect biomass assay. Have you been able to correlate the determined DNA concentration and the real dry biomass in your growth experiments?

Updated in the text accordingly. We did not attempt to correlate DNA concentrations to dry biomass, because the high salt concentrations (~20 g/l) of any residual medium will interfere with the accurate measurements of low bacterial biomass (mg/l).

Please enlarge the font of a, b, c, d in the Extended Data Fig. 1.

Done

Please describe the exact source of the given MAGs in Supplementary Table 2. Maybe I have overlooked this information, but where have the genome sequences of the 29 isolates and the deduced MAG sequences been deposited? Please add this information if not given. Please explain the difference between the different enrichment cultures ComA-C.

The source of each MAG is a co-assembly of metagenomic reads from 14 sequenced samples. The abundance of each MAG in individual enrichment replicates was determined by read mapping. Com A, B, and C represent three replicate enrichment cultures initiated from the same seawater sample but propagated in separate bottles. These communities showed similar sugar degradation profiles (Extended Data Fig. 1a) and broadly consistent community composition at the coarse-grained taxonomic level. Extended Data Fig. 1b shows that most strains were detected in at least two out of three replicates. However, we were unable to extract robust patterns of co-occurrence due to the limited sample size and the stochasticity of community assembly (see Response R3.4).

All raw sequencing reads, assembled MAGs, and isolate genomes are available under https://www.ncbi.nlm.nih.gov/bioproject/PRJNA996876, in accordance with FAIR principles. Accession numbers for each genome are provided in Supplementary Table 2 and reported according to MIMAG standards.

Referee #4: biochemistry

The manuscript, entitled: "A division of labor controls the degradation of fucoidans in the ocean" by Sauer et al, provides insights into how communities collaborate to metabolize the heterogeneous brown alga polysaccharide, fucoidan. Fucoidan has long been realized to be chemically complex, a feature which is believed to contribute to its recalcitrance and persistence in the environment. Descriptions of carbohydrate active enzymes (CAZymes) involved in dismantling the polysaccharide are limited in the literature. The authors have taken an ecological approach to identify guilds of marine bacteria that exclusively consume either the sidechains or

backbone of fucoidans, and determined that these guilds partner together to perform more complete degradation of fucoidan. These relationships appear to be conserved independently of the fucoidan source and chemistry (although the structure of these fucoidans were not characterized) and maintain a high correlation between communities. Although an exciting concept, it was reminiscent of what has been described for other syntrophic relationships. Surprisingly, an in-depth structural characterization of the commercially sourced polysaccharides (e.g., 1D and 2D 1H NMR, glycome profiling, linkage analysis, oligosaccharide chromatography with characterized standards) was not conducted in the study, and no new structures of fucoidans were described. Relying on commercial sources that are crudely characterized limits the reach of their conclusions. In addition, the authors did not provide new mechanistic insights (families, catalytic mechanisms, or structures) into how enzymes operate on the different substrate groupings. Without these or other insights to support mechanisms driving the foraging behaviors, it is difficult to justify acceptance in a journal as prestigious as Nature. More details on these topics are provided below.

Fucoidan Structure

Based upon the title and abstract, the expectation for this study was that there would be systematic characterization of fucoidan structure demonstrating firstly, the fine chemistry of the substrates, and secondly, how this structure systematically changes based upon encoded enzyme activities. Based upon the abstract (Line 23-24): "Despite variation in fucoidan structure across different types of algae, the metabolic functions of degraders remained conserved…". I was expecting a detailed analysis on the variation in fucoidan structure. Also, it was not clear where the schematic structure shown in Fig 1A was derived from. Some linkages are shown, some are unknown. What method was used to derive this structure?

Knowing the polysaccharide fine-chemistry enables the substrate to be partnered with PUL CAZyme specificity. There is precedent for in the CAZyme field using multiple complex polysaccharides (RG-II (10.1038/nature21725), yeast alpha-mannan (10.1038/nature13995), xyloglucan (10.1038/nature12907), pectin (10.1038/s41564-017-0079-1), mucin (10.1038/s41564-024-01911-7), etc) . Of note, three of these papers were published in Nature.

The authors use language such as "side-branches" and "rare monomers", which add intrigue, but provide little fundamental insights into the actual substrate structure. For example, a side-branch could mean a side chain (oligosaccharide) or branching point. More relevant description would be the monosaccharide, linkage, and degree of polymerization of the side change. Also, "rare" in this case implies it is not common, but based upon the explanation, this seems to mean less abundant than the sulfate fucosyl residues in the backbone. "Rare" is an ambiguous term, and one could argue that even it present at low amounts (<5%) of the total composition, if the polysaccharide is abundant, the rare monomers would become an abundant nutrient. Rare monosaccharides usually describe chemically distinguished or biological limited monosaccharides, neither of which is applicable here.

Also, it is widely recognized problem that polysaccharide purity can chemistry can vary between sources and preparations.

For these reasons, I believe a comprehensive glycomics analysis of fucoidan structure would strengthen the claims of this study. Relying on descriptions of commercial products and monosaccharide analysis does not account for biological and preparative variation, and importantly limits the ability to draw valuable, detailed conclusions on side chain chemistry, branching density, linkage, sulfation patterns, and other structural features.

We respect the reviewer's interest in structural differences between different fucoidans, but this work focuses not on the material itself but on how it is degraded. In fact, we demonstrate that despite structural variations the synergistic metabolic functions of degraders were surprisingly conserved. Thus, degradation outcomes can be quantitatively predicted from community composition - independent of fucoidan structures. Moreover, as described below, the requested structural information is already available for many cases.

Response R4.1. structure:

In the main text and Supplementary Note 1, we now clarify that the structure of *Fucus vesiculosus* fucoidan is extensively characterized (n = 6 studies). Fig. 1A represents a consensus repeating unit derived from solvolytic desulphation, linkage analysis, and multidimensional NMR (^{1}H, ROESY, COSY), with unresolved linkages explicitly indicated; notably, independent studies on the same commercial preparation report no major structural differences.

For the fucoidan panel, we summarize structural variation across species (n = 9 studies) in Supplementary Note 4, including backbone type, branching, sulfation, and side-chain composition. These inter-species differences clearly exceed expected variation between preparations, and for most substrates structural data are available for the exact materials used.

Given the extensive existing literature and intrinsic analytical limitations, additional de novo glycomics is unlikely to resolve remaining ambiguities. Our conclusions therefore rely on conserved architectural features—specifically a sulfated fucose backbone and lower-abundance side-chain sugars—which provide a sufficient structural basis for the observed functional patterns.

Response R4.2. purity:

We thank the reviewer for raising this critical point. Substrate purity was assessed by comprehensive monosaccharide profiling combined with mass recovery calculations (Supplementary Table 9). Most fucoidans were highly pure; only two substrates, *Fucus serratus* and *Durvillaea potatorum*, contained substantial glucose (73% and 45%, respectively), consistent with co-extracted laminarin. Reanalysis of community degradation at full monomer resolution shows that laminarin-derived glucose is rapidly and nearly completely depleted across all communities, irrespective of strain composition. In contrast, simple communities (e.g., F12, F40, F94) do not access fucoidan-bound fucose. Degradation increases only upon addition of complementary strains, indicating that community function is constrained by enzyme repertoire rather than the availability of alternative substrates. For these two substrates, all measurements and model predictions refer exclusively to the fucoidan-derived fraction of the resource pool. These revisions are reflected in the main text, Fig. 3d, Extended Data Fig. 10, Materials and Methods, Supplementary Note 4, and Supplementary Table 9.

Guilds

Groups are divided into three ecological guilds: Degraders, exploiters, and scavengers (Line 77-82), which seems based on earlier nomenclature presented in D'Souza et al. (10.1073/pnas.2305198120 and references therein) from their study on alginate.

These roles seem analogous to other defining labels used, such as primary degraders, keystone species, etc and so forth described for other systems. Degraders are defined as microorganisms that contain sulfatases and fucosidases. It would seem that primary degraders would need access to the backbone, and in this capacity, inclusion of sulfatases makes complete sense. Fucosidases, however, require the presentation of terminal fucose which can be abundent in oligosaccharides or side chains, but need to be generated by fucanases (endo-acting enzymes) in polysaccharides. Worth mentioning here is that enzyme nomenclature could be more precise

and there is overlapping use of fucosidase, fucanase, and "fucoidanase". See line 144. Fucoidanase has been given the EC number: 3.2.1.211 (formally 3.2.1.44; doi.org/10.3389/fmars.2023.1129982), and defined as a poly[(1->3)-alpha-L-fucoside-2/4-sulfate] glycanohydrolase. This reference activity does not seem to be exclusively assigned to the activities mentioned in the manuscript. It would be helpful for the more general reader if the enzyme activities could be defined and used consistently.

Response R4.2 EC numbers.:

We thank the reviewer for this important point. We now cite Pollak et al. (2021) and Pontrelli et al. (2022) to acknowledge the origin of the guild terminology. We further reworked our sequence annotation and report EC numbers, where available, in Supplementary Table 4 as detailed in Response R4.2. In addition, we added Supplementary Note 3 that summarizes the current state of fucoidan-degrading enzymes and associated EC classifications. Finally, we revised the manuscript throughout to ensure consistent and precise use of enzyme nomenclature.

On Line 111 the authors introduce "generalists". Doesn't this weaken the hypothesis that bacteria are clustered into functional guilds? The concept of the generalist should be introduced earlier as another functional grouping.

The presence of generalists does not contradict the guild framework, as metabolic traits are expected to form a continuum rather than discrete categories. We therefore introduce generalists at the point where experimental evidence supports their classification and clarify this in the revised manuscript.

Lines 140-146 describe the observation that enzyme copy number and diversity do not necessary correlate with fucoidan degradation. This was a very intriguing result, and the authors use it to support their hypothesis about the division of labor being a requirement for fucoidan degradation. Could it also be explained by substrate heterogeneity and availability in Nature (i.e., Chemically distinct fucoidan from different sources)?

We clarified our intended meaning of lines 140-146 in the main text. We agree that substrate heterogeneity may contribute to variation in degradation capacity. However, our data indicate that interaction-dependent effects are a dominant factor: for example, strain F56, despite encoding >100 enzymes but showing only ~5% degradation in monoculture, reaches ~17% degradation on residual substrates of V69 and ~50% in co-culture with F94.

Polysaccharide Utilization Loci

PUL description (Line 91-92). Extended Data Fig. 2A appears to be a relatedness chart of enzymes belonging to families associated with fucoidan degradation. This is not the conventional way to depict PULs in the literature, which are contiguous regions of genes active on a common substrate, under co-regulation, usually contain a TonB-dependent transport system (SusC/D-like proteins). PULs also typically contain unknown proteins or enzymes from other families not commonly associated with a substrate. Representing the complete set of genes in the figure is commonly done to inform enzyme discovery and is of interest to CAZymologists. If the authors do not wish to disclose this information, it does not seem prudent to describe these gene collections as "PULs". In addition, displaying PUL architectures is useful for visualizing syntenic relationships between microbes. It would be very interesting to see the synteny or relatedness between genes that clustered on a plasmid (G88) and genomically (F56) (Lines 97-98).

We thank the reviewer for this important point. We now explicitly show the presence of SusC/D-like transporters in Bacteroidetes PULs (Response R1.1) and clarify terminology in the manuscript. We note

that similar polysaccharide-degrading gene clusters lacking SusC/D have been described in other taxa and are commonly referred to as non-canonical or PUL-like systems (McKee et al., 2021).
Extended Data Fig. 2A,B demonstrate that both enzyme composition and sequence content of these loci are highly diverse, with limited conservation across strains; only a small number of PULs (e.g., PUL_12|Flavo56 vs PUL_8|Flavo40) show significant similarity. We therefore opted not to emphasize synteny, as it is largely uninformative in this system. Instead, we highlight representative PULs (e.g., PUL6|Verruco25; PUL1/2|Flavo56) that contain experimentally validated enzymes in Supplementary Fig. 8, and show the plasmid (G88) and chromosomal localization (e.g., in F56) in Supplementary Fig. 4. Fully annotated loci are provided in Supplementary Data 3 in GFF3 format.

Enzyme Classification

Classification of CAZymes (Lines 395-412). The authors have selected a low resolution approach to defining enzyme functions (dbCAN family assignment). Many of the families mentioned are polyspecific, and therefore, a more detailed analysis of sequence (e.g., dbCAN subfamily assignment, SACCHARIS) or structural motif relatedness (e.g. CUPP, SSNpipe) would be helpful.

Response R4.3. enzyme classification:

We refined our sequence analysis approach by comparing all identified fucoidan enzymes to characterized CAZymes using BLAST and phylogenetic trees (SACCHARIS) described in Materials and Methods, lines 497ff. In short:

- We now report closest homologs, EC numbers (where available), and annotation confidence levels in Supplementary Table 3.
- We incorporated recent functional updates, including assignment of GH141 homologs as endo-fucoidanases based on recent characterization (https://pmc.ncbi.nlm.nih.gov/articles/PMC12786026/).
- For 211/313 enzymes, predicted activities are consistent with known fucoidan linkage chemistry. In addition to established fucoidan-active enzymes, predicted rare-sugar activities include GH36/GH97 (α-galactosidase), GH92 (α-mannosidase), GH115 (α-glucuronidase), and GH31/GH39/GH120/GH30 (xylosidases). The activity of GH36, GH39, GH92, and GH97 was also confirmed experimentally (Fig. 2h and see response R3.3).
- Due to strong sequence divergence, exact linkage specificities cannot be reliably resolved; however, at the level relevant to fucoidan degradation, key CAZyme families map to one dominant coarse-grained activity classes, which we detail in Extended Data Figure 3.
- Phylogenies for selected families (e.g., GH97, GH92) are provided in the Supplementary Fig 6 and 7.
- dbCAN subfamily assignments are not available for the families analyzed here.

For example, the authors indicate GH92 and GH130 are fucoidan-associated GH families due a predicted mannosidase activities; however, these families encode alpha- and beta- activities, respectively. Are the authors suggesting that fucoidan contains alpha- and beta-linked mannose?

We now provide biochemical evidence that F56|GH92_E is a fucoidan mannosidase with 32% homology to AAQ66720.1 | α-1,3-mannosidase (PG1711) (Supplementary Figure 6 &7). Our tested GH130 remained inactive. Notably, by characterizing four novel alpha-galactosidases active on fucoidan, we show the presence of alpha-linked galactose in this fucoidan. See also response R3.3).

Also, it is not clear what is meant by "debranching". Typically, debranching refers to an enzyme that cleaves an oligosaccharide at a branch point and removes it from the main chain (e.g., starch pullulanase). It is not clear if the enzymes discussed in this study are predicted to be exo-acting, i.e. systematically trimming monosaccharides from the non-reducing end to the backbone, cleave internally along the sequence of the branched oligosaccharide releasing small oligosaccharides (e.g. endo-glycosidase), or specifically cleave the branch point, releasing the entire oligosaccharide? If a combination of the above, perhaps referring to these as side-chain degrading enzymes would clarify this for the reader?

We thank the reviewer for this important point. We have abandoned the term "debranching" and now use precise EC-based terminology where possible, or refer more generally to enzymes targeting fucose and sulfate linkages vs enzymes that target side monosaccharides.

As clarified in Responses to R4.2 and R3.3, these enzymes are predominantly predicted to be exo-acting glycosidases acting on non-fucose residues, and there is currently no evidence to distinguish between specific debranching mechanisms (e.g., branch-point cleavage versus terminal trimming).

Lastly, many enzymes were identified, assigned to PULs, and putative activities assumed; however, there was no biochemical characterization of enzymes conducted in this study. With the discovery of so many putative CAZymes with functions in dismantling the side chains and backbone of fucoidan, defining properties of the degrader and scavenger roles, structure-function characterization of representative enzymes is warranted.

Done, see response to R3.1. and R3.3.

Referee #5: co-reviewed with Referee #4

Major Comment

My expertise is in carbohydrate analytical chemistry, and my review focuses on this aspect of the manuscript. The study relies primarily on LC-MS-based monosaccharide profiling before and after bacterial growth. Although rapid, this approach has uncertain reliability for sulfated polysaccharides such as fucoidans, which are strongly anionic and may bind to charged surfaces of cells, particles, or pellets. As a result, only a fraction of the fucoidan may remain in the supernatant after bacterial growth. Evidence of complete recovery in the supernatant was not provided, and additional data are needed to rule out nonspecific binding. Moreover, monosaccharide analysis alone cannot capture critical structural features such as linkage patterns or the degree and position of sulfation. More importantly, the commercial fucoidans were not adequately characterized: at a minimum, glycosidic linkage analysis and high-resolution solutionstate 1D and 2D 1H and 13C NMR spectra are required to establish structural features and to exclude contamination by other polysaccharides (e.g., laminarin, alginate). Molecular weight and molecular weight distribution of the fucoidans must also be reported. Commercial lots can vary substantially with species, provider, harvest season, and environmental conditions. For fucoidans degraded by bacteria, LC-ESI-MS/MS analysis of released oligosaccharides in medium supernatant, even on selected samples, would provide essential mechanistic support. Without these data, the work remains overly dependent on monosaccharide-level measurements, leaving the structural and mechanistic conclusions insufficiently supported. In addition, the

accuracy and consistency of carbohydrate terminology and nomenclature should be improved, as detailed in the Minor Comments.

As discussed in response to other reviewers, we consider that the concerns raised here have already been comprehensively addressed.

- Analytical validity and recovery: We demonstrate quantitative mass recovery, supporting the robustness of the LC–MS approach for fucoidan (Response R3.8.).
- Structural sufficiency: Existing structural knowledge is extensive and now summarized (Response R4.1.); additional structural analyses would - in our view - not strengthen the conclusions regarding microbial interactions.
- Substrate purity and definition: Composition, molecular weight, and potential co-extracted polysaccharides were explicitly assessed and accounted for (Response R4.2.).
- Mechanistic support: Independent evidence from enzyme characterization (Response R3.3.) and species abundance patterns (Reviewer 2) provides complementary support for the inferred mechanisms.
- Terminology and clarity: Carbohydrate nomenclature and descriptions have been revised for consistency and precision throughout the manuscript.

Minor Comments.

Line 117: "the concentrations of monomers bound within fucoidan." What about the free fucose present in the medium, as well as the fuco-oligosaccharides in the supernatant?

Response R5.1.:

See response R1.2. & R3.7.: We now included time-course measurements of free monosasccharides in the culture supernatants, showing transient accumulation of fucose, xylose and galactose in a few degraders strains. Regarding oligosaccharides, currently there is no analytical method nor analytical standard available to resolve the expectedly low concentrations of transient fucoidan oligosaccharides in a salty matrix.

Furthermore, how were negatively charged fucoidans bound to the bacterial surface, particles, or pellets in medium, and therefore excluded from the supernatant, accounted for? Is there any evidence to rule out such binding?

In response R3.8., we show that the mass recovery for the main Fucus vesiculosus fucoidan is 99%. The time-course measurements show that if not degraded, concentrations of fucoidans stay constant during growth in the presence of cells. To our knowledge, the interaction between fucoidan and proteins occurs with excess protein at pH 3.8 (https://www.nature.com/articles/s41564-025-02240-z), which is different from our buffered pH 8 conditions in the medium.

Line 177–178: "We investigated cross-feeding using purified residual fucoidan (>1 kDa)." How was it determined that the residual fucoidan has a molecular weight greater than 1 kDa? Was any chromatographic or mass spectrometry method used to verify this? Furthermore, what is the molecular weight or molecular weight distribution of the commercial fucoidans prior to enzymatic treatment?

In Supplementary Note 1, we now describe the average molecular weight of fucoidans to be 100 kDa. A dialysis membrane with a 1 kDa cutoff was used to purify residual fucoidans and it is generally accepted that the residuals material would have a molecular weight larger 1 kDa. No further analysis was performed.

Line 256: “with dozens of glycosidic linkages.” This statement would be more convincing if glycosidic linkage analysis (ideally methylation-GC-MS) were conducted to characterize the linkage patterns of the substrates. For the oligosaccharides released by enzymes, it would also be informative to analyze their linkages using LC-ESI-MS/MS.
As outlined in response R4.1., fucoidan structures are sufficiently characterized, and additional linkage analysis is not required to support our conclusions. In Supplementary Note 1, we summarize available data for Fucus fucoidan, where methylation–GC–MS studies have resolved at least 17 distinct glycosidic linkages, already indicating substantial complexity. This is likely an underestimate, as sulfation introduces additional heterogeneity that is not captured by standard linkage analysis, and while NMR or Raman spectroscopy can quantify sulfate content, they do not resolve the precise linkage context of sulfated residues in intact polymers. Although LC–ESI–MS/MS of oligosaccharides can provide higher structural resolution, fucoidan lacks defined depolymerization strategies, and chromatographic separation of sulfated oligosaccharides typically requires specialized LC–MS setups (e.g., ion-pairing methods), limiting comprehensive reconstruction of native linkage patterns.

Line 282–285: “Thus, our work establishes a conceptual and practical framework to process increasingly important brown algal biomass and might extend to other polysaccharides with similar structures such as xylans and mucins.” Caution is needed when referring to applications 1 beyond fucoidans. The glycosidic linkage patterns of mucins and xylans differ from those of fucoidans. Moreover, grouping them together as “other polysaccharides” is inaccurate: xylans are polysaccharides, whereas mucins are glycoproteins with a central polypeptide core extensively decorated with O-linked oligosaccharide side chains. Please revise this description to avoid confusion and improve accuracy.
We revisited the statement and decided to only keep xylans as possible examples.

Line 296: “Fucoidan from Fucus vesiculosus was obtained from Sigma-Aldrich (F8190). Sources for other fucoidans were Fucus vesiculosus (Marinova FVF2021547), Fucus vesiculosus (Biosynth 298 YF57714)….” The description is unclear and confusing. Why are fucoidans from the same species, Fucus vesiculosus, listed separately, even though they all originate from the same seaweed? It would be clearer to group Fucus vesiculosus fucoidans obtained from different providers in one sentence, and then describe fucoidans from other brown seaweed sources in a separate sentence. In addition, consider including a table summarizing the structural differences among these fucoidan samples (e.g., monosaccharide composition, linkage composition, molecular weight). It is also important to ensure that these commercial samples are not contaminated with alginates, another major class of brown seaweed polysaccharides. The purity of commercial polysaccharides can vary between lots; therefore, it is advisable to characterize commercial samples used as substrates for bacterial growth. Finally, please provide the lot numbers of the commercial products so that others can repeat the experiments with the same lots whenever possible.
We clarified the text section according to your suggestion and included batch numbers when applicable. According to your suggestion, we included Supplementary Note 1 and 4 that described the structure of the used fucoidans in detail. Hence, the functional conclusions in this manuscript are sufficiently supported by the compositional data shown in the main text, see response R4.1. Purity is addressed in Response R4.2.

Line 302–304: "Internal standards for LC-MS analysis included D-galactose-13C6 (Sigma-Aldrich, 605379), D-mannose-13C6 (Sigma-Aldrich, 592994), and PMP-[d_5] (CAS 1228765-67-0), custom-synthesized by BOC Sciences (Shirley, NY, USA)." The inclusion of 13C-labeled mannose and galactose as internal standards is appropriate; however, because fucose is the major monosaccharide in fucoidan, it would be beneficial for quantitation if 13C-labeled fucose were also included as an internal standard.

Fully labelled L-fucose-$^{13}C_6$ is not commercially available. In our workflow, ^{13}C-labelled mannose and galactose are used as process controls to correct for inter-sample variability during extraction, hydrolysis, and derivatization, which are not expected to be further improved by inclusion of an additional monosaccharide standard. To account for compound-specific differences in ionization efficiency, including for fucose, we employed deuterated PMP (PMP-[d_5]) as an internal standard, which corrects for variability at the ion-source level across all derivatized monosaccharides.

Lines 681–683 (Caption of Figure 1): "a, Structure of fucoidan from Fucus vesiculosus and corresponding monosaccharide composition shown as a bar graph. Monosaccharides are depicted following the Symbol Nomenclature for Glycans; glycosidic linkages are indicated as text labels." For Figure 1a, how was the structure of fucoidan determined in terms of monosaccharide composition and glycosidic linkage pattern? Is there supporting evidence from glycosidic linkage analysis (e.g., methylation-GC-MS) or NMR to substantiate the proposed structure?

We revisited the structure and clarified accordingly. The structure of this fucoidan is now extensively described in Supplementary Note 1.

As noted in Lines 296–298, Fucus vesiculosus fucoidans were obtained from three different companies. How pure are these commercial fucoidans, and how variable are they among the three sources in terms of monosaccharide composition, glycosidic linkage distribution, and degree of sulfation at different positions?

Monosaccharide composition is shown in Figure 3d and Extended Data Figure 10a. No additional characterization was performed. Purity is addressed in response R4.2.

It is confusing to use the same three-letter abbreviations for both enzymes in Figure 1c and monosaccharides in Figure 1f. In carbohydrate chemistry, three-letter abbreviations are 2 conventionally reserved for monosaccharides. For example, "Gal" is used exclusively for galactose (as shown in Figure 1f) and should not be used to denote galactosidases (as shown in Figure 1c).

Corrected.

Line 21, Line 128, Line 243, and Line 247 use "rare monomers"; Line 135, Line 175, Line 208, Line 220, and Line 228 use "rare monomer"; and Line 260 uses "rare-monomer." The term "rare monomers" is confusing in carbohydrate analytical chemistry, as it may be misunderstood as referring to rare sugars (e.g., anhydro sugars) that are uncommon in nature. However, the monosaccharides found in the side chains of fucoidans are not rare; they are common sugars that also occur in higher plant cell walls and are simply present in minor amounts in fucoidans. It is therefore recommended to replace the term with "minor monomers" or "minor monosaccharides" for greater accuracy.

In lines 74 and 1214, we now define "rare-sugar monomers" as the less abundant non-fucose monosaccharides.

Line 329–330: Please add a reference for the phenol-sulfuric acid method.
Done

Minor comments:
Line 7 and Line 10 use "centre," while Line 323 and Line 730 use "center." Please make this consistent.
Done

Line 19: "Metabolomic analysis" is too broad in this context. The actual method used is LC-MSbased monosaccharide analysis. I therefore suggest changing it to "LC-MS-based monosaccharide analysis" or simply "glycomic analysis," which would be more accurate.
We respectfully disagree and prefer to stick with the original term that is sufficiently defined in this context.

Line 26: "complex macromolecules." The term "complex macromolecules" should be replaced with "complex polysaccharides," as "macromolecules" is too broad in this context. If the authors prefer to retain the original expression, I suggest revising it to "complex biomacromolecules" for greater accuracy.
Line 31, "macromolecules". Change to "biomacromolecules" for term accuracy.
We thank the reviewer for this suggestion. We replaced "complex macromolecules" with "biopolymers," which provides a more precise and field-appropriate description by explicitly capturing their polymeric structure and biological origin, while avoiding the broader and less specific term "macromolecules."

Line 53: "monomer composition." I suggest changing this to "monosaccharide and linkage composition."
We reworked this sentence according to your suggestion.

Line 64: Typo "of from" should be corrected. Line 68, Line 94, Line 605, and Line 730 use "rare sugars";
done

Line 109 uses "rare side-chain sugars"; and Line 110 uses "rare-sugar." As noted above, please change these to "minor sugars," "minor side-chain sugars," and "minor-sugar," respectively.
We have carefully reworked our terminology, clarified its definition in the main text, and now use it consistently throughout the manuscript.

Line 114: The term "Monomer degradation" is confusing. Please change it to "monosaccharide composition analysis" or "backbone depolymerization." Accordingly, the subtitle should be revised to either "Monosaccharide composition analysis reveals functional complementarity among degraders" or "Backbone depolymerization reveals functional complementarity among degraders."
The respective paragraph changed in the revised manuscript and we define our preferred term "monomer degradation" there.

Line 116: The phrase "measure degradation at the monomer level" should be revised to "residual monosaccharides in enzyme-treated fucoidans" for greater accuracy.

We use residual monosaccharides to infer degradation and we ensured the our preferred term of "monomer degradation" is defined in the manuscript.

Line 119: Change "monomers" to "released monosaccharides."

Done

Line 234: Change "monomer composition" to "monosaccharide composition."

Done

Line 245: Change "F. vesiculosus" to "Fucus vesiculosus" for format consistency.

We spell out *Fucus vesiculosus* the first time in each section and then abbreviate *F. vesiculosus* afterwards.

Line 298: "Fucus evanescence" is an incorrect Latin name; the correct form is "Fucus evanescens."

Done

Line 302: "D-galactose";

Done

Line 303: "D-mannose." The capital letter "D" should be formatted as a small capital for accuracy in carbohydrate nomenclature, following IUPAC recommendations. The same applies to all instances of "D" and "L" designations for monosaccharides in Lines 385–394 and throughout the manuscript.

Done

Lines 313–314: "The pH was buffered pH=8.0 by either bicarbonate in both the enrichment and plate media…." The phrasing is awkward and repeats "pH" unnecessarily. Please revise to: "The pH was buffered to 8.0 with bicarbonate in both the enrichment and plate media…."

Done

Lines 317 and 326: "Fucus vesiculosus" is incorrectly formatted. Latin binomials should be italicized.

Done

Lines 318–319: "carbon sources (Acetate, Citrate, Xylose, Galactose, Mannose, Glucose, Fucose, Cellobiose, Tryptone)." These names should not be capitalized. Please revise to lowercase: acetate, citrate, xylose, galactose, mannose, glucose, fucose, cellobiose, and tryptone.

Done

Line 321: Change "30 March 2019" to "March 30, 2019" (or "March 30th, 2019"), for consistency with standard date formatting.

Done

Line 323, Line 456, Line 472, and Line 474 use "minutes," while Line 442 and Line 473 use "min." For consistency, please standardize the format by using either "minutes" or "min" throughout.

Line 331: Change "1 mL concentrated sulfuric acid" to "1 mL of concentrated sulfuric acid."

Done

Line 332: "50 °C"; Line 339; Line 346: "−80 °C";

Done

Line 524: "20 °C"; Line 536: "–20 °C." In contrast, Line 451: "100°C"; Line 456: "70°C"; Line 476: "40°C," "10°C," and "625°C." These are just some examples. The spacing between the number and the °C unit is inconsistent. Please standardize the formatting throughout the manuscript.

Done

Line 433: Change "3 mL MBL medium" to "3 mL of MBL medium."

Done

Line 455: "were derivatized with 75 μL of 0.1M PMP in 2:1 methanol:ddH2O with 0.4 M ammonium hydroxide." There is a space between "0.4" and "M" but not between "0.1" and "M." Please improve the formatting consistency.

Done

Line 468: "2.1 mm X 50 mm reversed-phase column." The format of "X" is incorrect. It should not be a capital letter "X"; instead, it should be the multiplication sign "×". 4

Done

Line 505: i (i ∈ {Fuc, GlcA, Gal, Man, Xyl, Rare, Total}). In contrast, in Equation (3) below Line 565: m ∈ {fuc, man, gal, xyl, glcA}. Please change "fuc, man, gal, xyl, glcA" to "Fuc, Man, Gal, Xyl, GlcA" for accuracy and consistency. The formatting for listing items (e.g., "A, B, and C" vs. "A, B and C") is inconsistent. For example, Line 313: "340 mM NaCl, 15 mM MgCl2, 6.75 mM KCl and 1 mM CaCl2" (no comma after KCl), whereas Line 510: "fucose, glucuronic acid, galactose, mannose, and xylose" (with a comma after mannose). Both formats are acceptable, but one style should be chosen and applied consistently throughout the manuscript. Please correct this inconsistency.

Capitalization and list formatting were standardized consistently throughout the manuscript, with the Oxford comma applied only where needed for clarity.

In Figure 1a, I suggest using the short names Fuc, Man, Gal, Xyl, and GlcA instead of the full monosaccharide names. If the authors prefer to retain the full names, "GlucuronicAcid" should be corrected to "Glucuronic acid."

Done, we kept it full name.

In Figures 1c and 1f, the first letters of the monosaccharide abbreviations "fuc," "gal," "xyl," "glcA," and "man" should be capitalized.

Done

In Figure 3, the image appears to be partially trimmed on the left, as the left side of the letter "d" in panel 3d is cut off. For Figure 3d, I suggest using the short names Fuc, Man, Gal, Xyl, and GlcA instead of the full monosaccharide names. If the authors prefer to retain the full names, "GlucuronicAcid" should be corrected to "Glucuronic acid."

Done

Please add units to the monosaccharide composition in Figure 3d. For example, if the unit is molar composition, a bracket with "mol%" should be included. This unit should also be added to the caption of the figure, "d. Monosaccharide composition." In addition, if the values are expressed as molar percentage (mol%), I suggest changing the scale in Figure 3d from 0.01.0 to 0–100.

Done

Lines 224 and 745: e.g. should be italicized.

Done

Page 22, Extended Data Fig. 5: I suggest using the short names Fuc, Man, Gal, Xyl, and GlcA instead of the full monosaccharide names. If the authors prefer to retain the full names, "GlucuronicAcid" should be corrected to "Glucuronic acid."

In the reference list, References #2, #5, #9, #10, #14, #16, #23, #34, and #59 have all words in their titles capitalized, whereas in the other entries only the first word of the title is capitalized. Please correct this inconsistency.

We thank the reviewer for pointing this out. We have standardized the capitalization of all reference titles to ensure consistency with journal style.

Referees' comments:

Referee #2 (Remarks to the Author):

The authors have now added data to classify the ecological interactions among the strains. These data are very helpful and informative and show that the great majority of interactions between the strains are either negative or, when there is a positive effect, they are either commensal or parasitism. There is one example of mutualism in what looks like around 20 interactions that have at least one positive edge(Fig 2b).

To have everything here, I will paste in here to two scenarios that the previous version of the manuscript left open:

"Case 1: the cooperative division of labor. As a simplified version of fucoidan, imagine a complex molecule made of two monomers A and B, where A is attached to other As to form a backbone, and B is a side moiety that is attached as a single monomer to some of the A monomers on the backbone. Now take two species, i and j, where i can digest the A-A links (and uses A as a nutrient) and j can digest the A-B links (and consumes B). However, because of the way that that two monomers arrange, it is hard for i to digest A-A when B is attached to one of the As. In addition, when A is in a long chain, it is hard for species j to remove B. As a result, when species i cuts some of the A-A links, this allows j to remove the Bs better, which means it can now gain nutrients more easily. In addition, the removal of the Bs helps A to digest the A-A chain and gain A monomers to feed it. The details are not so important for the point but what is important is that both species here help the other to perform their digestion and obtain nutrients. So, this can be argued to be a meaningful division of labor, where both species benefit from the full breakdown of fucoidan, and it also looks like a cooperative interaction (so long that both species also increase in abundance due to the presence of the other strain).

Case 2: no cooperation, no division of labor. Take the same hypothetical compound and two species. However, this time while species i helps j to perform its digestion, species j has no impact on species i. Firstly, this is a commensalism and not cooperation but it is also not a meaningful division of labor because species i has no functional interest or benefit in the degradation by species j and they are not dividing labor to achieve any end goal. Species i is doing what it always does and only the second species benefits from the interaction. If I understand it correctly, the synergism measure in the manuscript would identify this case (as well as the last one) as synergism, cooperation and a division of labor. But the two cases are quite different because in this last case, the goal of complete degradation of the complex molecule only exists in the mind of the experimenter, it is not something that the bacteria have an ecological or evolutionary shared interest in achieving."

As the authors also state in their response, the new data support the second scenario i.e. there is no cooperation and no true division of labor, beyond that perceived by an experimenter who choses

fucoidan degradation as a perceived goal. The reason I am repeating myself above is that, despite having removed the term from the title, the authors are still presenting things as a division of labor throughout and are, therefore, still implying to many that the bacterial species are working cooperatively to achieve a goal.

For example, the results section ends with the concluding sentence: "Together, these findings suggest that functionally specialized and complementary fucoidan degraders frequently co-occur in the ocean, supporting synergistic interactions and division of labour as an ecologically relevant strategy for the degradation of complex marine fucoidans. "

I believe that such statements will be confusing to readers and I am sure many people will miss the one figure panel (2b) and brief text that states that the positive effects are actually all tied up in commensal or parasitic relationships.

If the authors wish to discuss the division of labor, for clarity, I would suggest i) removing the use of the term division of labor from the main body of the text and ii) adding a paragraph to the discussion that cover the ecological and evolutionary bases for the degradation i.e. the authors find evidence that it can emerge spontaneously and robustly from the evolutionary self-interest of each strain, without the need for coordination and cooperation. This finding is itself an important result for the field and one that helps to explain the evolutionary and ecological stability of this fascinating biology, so I would hope including this discussion will help raise the impact and clarity of the paper. Moreover, to bring in the division of labor, this section can also discuss that in one case it appears that there is an example of two species working together and so, in a small number of cases, there may well be a division of labor and cooperation too in these systems.

For the abstract, the use of division of labor could be in a sentence like "Most degradation is driven by negative or commensal relationships among species but in one case out of X we find evidence for a cooperative division of labor", but otherwise the term can be replaced with 'interaction' or something similar.

We thank the reviewer for the careful restatement, which helped us resolve ambiguities between the ecological/evolutionary scale of species-level data (Fig. 2b) and synergistic degradation on the functional level.

In our study, we use division of labour in a strictly mechanistic/functional sense: polymer deconstruction is partitioned across enzyme classes (e.g., backbone-active vs side-chain-active enzymes), and these complementary activities are distributed across different taxa. Our evidence supports enzyme-level functional complementarity and synergistic substrate processing. This functional usage is well established in this literature, including for complex-polysaccharide degradation specifically (Lindemann 2020, our ref 18; Tsoi et al. 2018; Enke et al. 2019), and carries no implication of altruism or coordination. This distinction between the functional (mechanistic) and evolutionary levels of division of labour is itself emphasised in the DOL literature (West & Cooper 2016; Giri et al. 2019), and we are explicit that our claims are functional.

Following the reviewer's suggestion, we made this separation explicit in the Results and Discussion and no longer infer an evolutionary cause for the observed partitioning. As the reviewer notes, the more parsimonious interpretation is that complete degradation emerges from the metabolic self-interest of each strain, without altruism. The expanded Discussion now sets this out, including how it could arise through neutral processes such as gene loss under drift, alongside physiological trade-offs and environmental filtering.

Changes made to the manuscript:

- We reserve "functional division of labour" for a single, defined use at first mention in the abstract, as a broad and familiar entry point for readers — a term that is standard and informative for this readership. We use "synergism" and "functional complementarity" throughout the main text.
- We expanded the Discussion to flag the Verruco69–Gamma88 pair as a genuine but exceptional case of cooperative division of labour, and to lay out the non-cooperative routes to the observed partitioning.
- We revised the Results section to make the distinction between species-level and functional level more explicit.

Referee #3 (Remarks to the Author):

The authors have satisfactorily addressed all of my points of criticism. In my view, the statistical analysis of the data—wherever possible in the experiments—has also been conducted appropriately.

Thank you for this positive evaluation and helping to improve the quality of the manuscript.

Referee #4 (Remarks to the Author):

Reviewer 4

The authors have done an excellent job of addressing my previous comments. Below are some final thoughts with some minor corrections suggested to further improve the manuscript.

Thank you

R4.1 the justification for previous characterization of Fucus vesiculosus fucoidan is sufficient, and the authors have addressed the issue of contamination in R4.2, identifying the presence of laminarian. The monosaccharide data (Fig. 3d and E Data Fig. 10) has strengthened the study. PULs. The inclusion of Supplementary Fig.8 is welcomed and strengthens the study. I would recommend adopting the colour scheme introduced by Martens et al (2008) 10.1016/j.chom.2008.09.007 for consistency. Similarly, the sugar symbols in Figure 1A (and throughout) should adhere to the Symbol Nomenclature for Glycans (SNFG) system: https://www.ncbi.nlm.nih.gov/glycans/snfg.html.

Done

The addition of phylogenetic analysis of CAZymes for prediction of specificities, inclusion of trees (Supplementary Fig. 6&7), and preliminary enzyme kinetics provide support the authors conclusions. Biochemistry of predicted enzymes was lacking previously, and has strengthened the study. For example, the demonstration of GH92 activity on fucoidan, confirms the presence of alpha-mannose in fucoidans. The inactive GH130 also is consistent with its expected activity and the absence of beta-mannose linkages. The selection of pH8 for GH assays was surprisingly high. Did the authors conduct pH optima to determine this condition?

We did not determine the pH optima of the tested enzymes. Instead, we performed the assays at pH 8.0 using the same buffer and salt conditions as in the bacterial growth medium. We acknowledge that individual enzymes may exhibit different pH optima and that the measured activities therefore do not necessarily represent their maximal catalytic rates. However, our objective was to assess enzyme activity under conditions approximating the marine environment and the physiological conditions experienced by the source organisms, rather than to perform a complete biochemical characterization.

Line 136-7: Amount of free fucoidan appears inversely proportional to free fucose in V4 (Ext. Data Fig 5). There is a similar effect for free xylose in V25 and V69. I am not sure if the authors fully address this observation. I am curious if the accumulation may be related to transport efficiencies as monosaccharides (generated by hydrolytic processing) are not optimal substrates for SusC/D transport. Is there a potential relationship here? V4 seems to be lacking the fucose permease, TonB-dependent transporter, and other transport systems.

We thank the reviewer for this suggestion. We have clarified in the main text that transient extracellular monosaccharide accumulation reflects hydrolytic release temporarily exceeding subsequent uptake. We also note that V4 encodes a fucose permease, as shown in Extended Data Fig. 1. Supplementary Fig. 4 shows only the SusC/D-like transport systems associated with the predicted PULs and therefore does not represent the complete transporter repertoire of V4.

Reviewer 5

Apart from the proper formatting for D/L enantiomeric configuration (e.g., Line 523-4; italicised and large capitals, Line540), all my other concerns have been addressed.

We thank the reviewer for noting that their other concerns have been fully resolved. Regarding the formatting, IUPAC-IUBMB guidelines (Rule 2-Carb-4.1, *Nomenclature of Carbohydrates*) mandates that D and L be formatted as upright (roman) small capitals. We have verified that all instances in our manuscript have been consistently formatted to this international standard. We appreciate the reviewer's excellent attention to detail.

Referee #5 (Remarks to the Author):

I co-reviewed this manuscript with one of the reviewers who provided the listed reports.
