# Supplementary material for: Synergistic degradation of fucoidans in the ocean
Source: Nature. 2026 Aug 26;658(8136):682–9. doi: 10.1038/s41586-026-10980-z (PMC13668718; doi:10.1038/s41586-026-10980-z)
Supplement: Supplementary file 2 [file 41586_2026_10980_MOESM1_ESM.pdf]

nature portfolio

https://doi.org/10.1038/s41586-026-10980-z

# Supplementary information

# Synergistic degradation of fucoidans in the ocean

In the format provided by the authors and unedited

# Supplementary Information for

# Synergistic degradation of fucoidans in the ocean

Andreas Sichert[1,2], Shaul Pollak[3], Taylor Priest[4], Akshit Goyal[5], Samuel Miravet-Verde[4], Shinichi Sunagawa[4], Otto X. Cordero[2]*, Uwe Sauer[1]*

**Affiliations**

[1] Institute of Molecular Systems Biology, ETH Zürich; 8093 Zürich, Switzerland.

[2] Department of Civil and Environmental Engineering, Massachusetts Institute of Technology; 02139 Cambridge, USA.

[3] Centre for Microbiology and Environmental Systems Science, Department of Microbiology and Ecosystem Science, Division of Microbial Ecology, University of Vienna; 1030 Vienna, Austria.

[4] Institute of Microbiology, Department of Biology, ETH Zürich; 8093 Zürich, Switzerland.

[5] International Centre for Theoretical Sciences, Tata Institute of Fundamental Research; 560089 Bengaluru, India.

*Corresponding authors. Email: sauer@imsb.biol.ethz.ch (U.S.); ottox@mit.edu (O.X.C.)

**Supplementary information - Table of contents:**

Supplementary Note 1: Structure of *Fucus vesiculosus* fucoidan ........ 2
Supplementary Note 2: Occurrence of isolates ........ 2
Supplementary Note 3: Enzyme classification and activity ........ 3
Supplementary Note 4: Fucoidan diversity ........ 3
Supplementary Figure 1 ........ 5
Supplementary Figure 2 ........ 6
Supplementary Figure 3 ........ 7
Supplementary Figure 4 ........ 8
Supplementary Figure 5 ........ 9
Supplementary Figure 6 ........ 10
Supplementary Figure 7 ........ 11
Supplementary Figure 8 ........ 12
Supplementary Figure 9 ........ 13
Supplementary Table Legends ........ 14
Gel source data ........ 16

## Supplementary Note 1: Structure of *Fucus vesiculosus* fucoidan

Fucoidan from *Fucus vesiculosus* is a sulfated heteropolysaccharide dominated by fucose but containing smaller amounts of xylose, galactose, mannose and glucuronic acid[33,35,84]. The commercial fucoidan used in this study has an average molecular weight of ~105 kDa and a sulfate content of 24% (w/w)[33]. Structural analyses show that fucoidan consists of an alternating α-(1→3)/α-(1→4)-linked L-fucose main chain sulfated primarily at O-2 and O-3[34]. Branching of the main chain mainly occurs by substitutions of every second fucose at position C-4 by sulfate, α-(1→4)-linked fucose or β-(1→4)-linked xylose. Linkage analysis shows that 3-fucose, 4–and 3,4-fucose together account for ~52% of fucose, while a high proportion of terminal fucose residues (~27%) indicates numerous short fucose branches[33,85].

Together, the α-(1→3)/α-(1→4) fucose main chain and its short fucose branches form the fucose backbone of this fucoidan, accounting for ~80% of monosaccharides. In contrast, xylose, galactose, mannose and glucuronic acid occur only at low abundance and are therefore termed rare-sugars monomers that form partially resolved side-chain decorations on the fucose main chain. Consistent with this interpretation, linkage analysis detected ten minor non-fucose linkages (0.1–2 mol%), including multiple galactose linkages, indicating the presence of structurally heterogeneous rare-sugar side branches. Notably, short galactose-containing branches and glucuronic-acid substitutions have been structurally resolved in fucoidans from the closely related species *Fucus evanescens* and *Fucus serratus*, supporting the presence of analogous rare-sugar side branches in fucoidan from *F. vesiculosus*[86,87].

## Supplementary Note 2: Occurrence of isolates

Isolates of the fucoidan-degrading community are largely undescribed and underrepresented in current genome databases. Genome-based comparison to type-strain references using TYGS[88] showed that the degraders (Verruco25, Flavo12, Flavo56, Gamma88), the exploiters (Alpha02, Alpha14, Gamma21) and the scavengers (Alpha16, Alpha59, Alpha60, Alpha92, Alpha95, Flavo48, Flavo72, Gamma71, Gamma84) represent previously undescribed species (dDDH_d4 < 70%). To assess their environmental occurrence, we compared the degraders to genomes of the mOTUs database[55], which comprises a collection of >120,000 species represented by genomes from isolates and metagenome-assembled genomes. Comparisons to the mOTUs database was performed using the *classify* function of the mOTUs tool[89], which matches genomes to species through alignments of ten single-copy marker genes. Only four isolates (Gamma88, Verruco4, Flavo94 and Flavo40) were represented by species in the mOTUs database, which were distributed in up to 64 macroalgae-influenced coastal samples spanning the German Bight, North Atlantic, North Pacific and an isolation study from the algae *Saccharina sp.* in coastal China[81] (Supplementary Fig. 2a). Across the dataset, four samples contained distinct combinations of two degraders, indicating that members of our isolate collection can co-occur across geographically distant algal habitats (Supplementary Fig. 2b).

To gain further insights, 16S rRNA genes from the isolates were compared to the MicrobeAtlas database[79] using a 99% identity threshold (Supplementary Fig. 2c). Four degraders (Gamma88, Verruco4, Verruco69 and Flavo40) were globally distributed with >3,000 matches, whereas Verruco25 and Flavo12 were each detected only once and Flavo56 remained undetected. We detected the 16S rRNA gene of all isolates except Flavo56 and Flavo94 in a survey of macroalgal thallus-attached communities over an annual cycle in Roscoff, France[80]. This study identified *Roseibacillus*—represented by Verruco69 in our collection—as a core member of the *Ascophyllum nodosum* microbiome. Notably, Verruco69 co-occurred with Verruco4 in >2,000 samples, a pair that we show in our subsequent characterization exhibits a strong synergistic interaction. Together, these observations establish a direct link between our in vitro characterization and environmental co-occurrence patterns.

**Supplementary Note 3: Enzyme classification and activity**

Recent transcriptomic and proteomic analyses of fucoidan-degrading bacteria revealed the core enzymatic machinery responsible for cleavage of sulfated fucose residues. The most strongly upregulated enzymes during growth on fucoidan substrates belong to CAZyme families GH29, GH95, GH107, GH141 and GH168 and sulfatase families S1_15, S1_16, S1_17 and S1_22[3,4]. Fucoidan degraders often express more than ten different enzyme homologs within each family, which likely enables degradation of the highly ramified fucoidan structures. Biochemical characterization of representative enzymes supports this interpretation. Endo-acting fucoidanases from GH107, GH141 and GH168 cleave internal glycosidic linkages within sulfated fucans (EC 3.2.1.211–3.2.1.212)[39,44,45], whereas GH29 and GH95 α-L-fucosidases (EC 3.2.1.51 and EC 3.2.1.63) remove terminal fucose residues from partially desulfated fuco-oligosaccharides[40,43]. These reactions require S1-family sulfatases (EC 3.1.6.–), which remove sulfate esters that otherwise block glycosidic bond hydrolysis. In particular, sulfatases from the S1_25 and S1_22 subfamilies have been biochemically characterized on fucoidan: S1_25 enzymes function as exo-2-O-sulfatases that remove sulfate groups from terminal fucose residues in sulfated fuco-oligosaccharides, whereas S1_22 enzymes act as endo-sulfatases that remove sulfate esters from internal fucose residues within fucoidan chains[42]. To reflect their central role in fucoidan cleavage and desulfation, we collectively refer to them as enzymes that target sulfate and fucose linkages.

In addition, co-localization within fucoidan PULs of isolated degraders identified a broader set of putative enzymes predicted to act on non-fucose residues present in fucoidan side chains (Extended Data Fig. 3; Supplementary Table 3). These assignments were based on homology to characterized exo-acting glycoside hydrolases and compatibility with known fucoidan linkage chemistry. We grouped these enzymes into coarse-grained activity classes targeting galactose, mannose, glucuronic acid and xylose residues. These included α-galactosidases (GH36 and GH97; EC 3.2.1.22), β-galactosidases (GH2; EC 3.2.1.23), α-mannosidases (GH92; EC 3.2.1.24), α-glucuronidases (GH115; EC 3.2.1.131), α-xylosidases (GH31; EC 3.2.1.177) and β-xylosidases (GH39, GH120 and additional GH3/GH30 homologs; EC 3.2.1.37). Together, these putative exo-acting activities may remove galactose-, mannose-, glucuronic acid- and xylose-containing decorations from fucoidan side chains, although remain experimentally uncharacterized in the context of fucoidan degradation. Fucoidan PULs also encoded additional CAZyme families, such as GH117 β-galactofuranosidases (EC 3.2.1.146), for which no compatible substrate residue was detected in the fucoidans analyses here. These enzymes were therefore classified as hypothetical fucoidan-associated enzymes, highlighting unresolved functions within fucoidan PULs.

**Supplementary Note 4: Fucoidan diversity**

Fucoidans from Fucus species represent the most structurally resolved class of brown-algal fucoidans. *Fucus evanescens* is composed primarily of alternating α-(1→3)- and α-(1→4)-linked L-fucose residues, with sulfate groups predominantly at C2 and partly at C4, together with occasional acetylation[90,91]. The closely related *Fucus serratus* fucoidan exhibits the same alternating backbone but displays substantially higher branching, as approximately half of the 3-linked fucose residues carry trifucoside side chains attached at C4, while minor amounts of xylose and galactose occur in additional short side chains[87]. Minor constituents such as glucuronic acid have also been detected in this fucoidan class[33].

Despite this conserved backbone, fucoidans from Fucus species show pronounced temporal and spatial variability in fucose content and degree of sulfation, with sulfate levels typically higher in winter[54]. In addition, environmental conditions and population differences influence monosaccharide composition and sulfation patterns[92]. To test for these possible subtle

structural variabilities, we tested degradation of three *F. vesiculosus* preparation from two additional vendors located in UK and Australia.

Fucoidans from other brown algae exhibit distinct architectures. The fucoidan from *Cladosiphon okamuranus* consists primarily of α-(1→3)-linked fucose residues sulfated mainly at C4, carrying α-(1→2)-linked glucuronic acid branches[93,94]. In *Laminaria hyperborea*, the fucoidan is almost exclusively composed of fucose (~98 %) and forms a highly sulfated, branched α-fucan dominated by 1→3 linkages, with smaller contributions from 1→2- and 1→4-linked fucose residues and only trace amounts of galactose[85]. The fucoidan from *Durvillaea potatorum* contains an α-(1→3)-linked fucose backbone decorated with β-linked galactose branches, including β-(1→6)- and β-(1→3)-galactose residues, indicating a branched galactofucan architecture[33,95]. In contrast, the fucoidan from *Ecklonia maxima* contains a mixture of 4-linked fucose, galactose-containing linkages, xylose residues and uronic acids, together with detectable O-acetylation, suggesting a structurally heterogeneous sulfated polysaccharide with multiple backbone and branch motifs[33].

To quantify compositional variability across this substrate panel, we performed full quantitative monosaccharide analysis and combined these measurements with literature-reported sulfate contents[33,85,91] to establish a mass balance for each fucoidan (Supplementary Table 9). For five of nine fucoidans obtained from commercial suppliers, the sum of anhydro-corrected monosaccharides and estimated sulfate accounted for the majority of the weighed-in material, indicating a largely closed mass balance and high purity. In contrast, *F. serratus* and *D. potatorum* exhibited elevated glucose levels, indicative of co-extracted laminarin[84]. Additionally, *D. potatorum*, *E. maxima* and *C. okamuranus* displayed substantial unaccounted fractions (20–40%), likely attributable to non-carbohydrate components such as proteins. Together, these results indicate that LC–MS-based fucoidan degradation measurements capture between 19% and 100% of the available resource pool.

**Supplementary Figure 1**

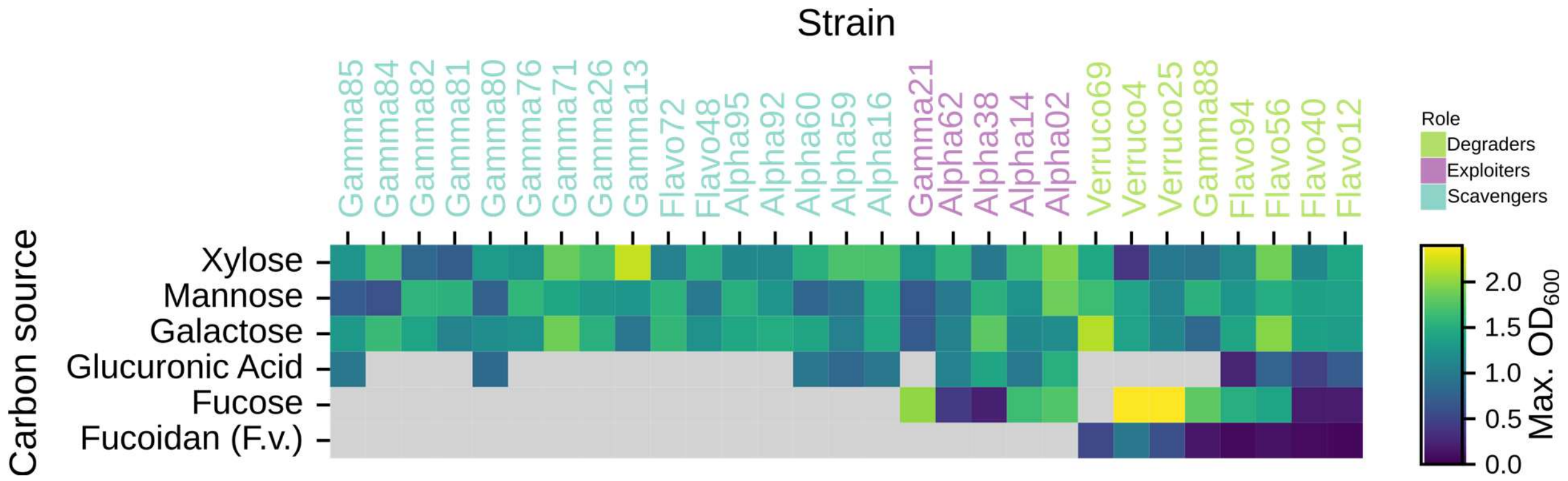


*Supplementary Fig. 1:* **Growth of isolates on different carbon sources.** The heatmap shows the average maximal growth of 29 bacterial strains on different carbon sources determined in three biological replicates. Strains are colour coded according to their predicted ecological role. Grey fields denote insignificant growth $OD_{600}$ <0.05.

**Supplementary Figure 2**

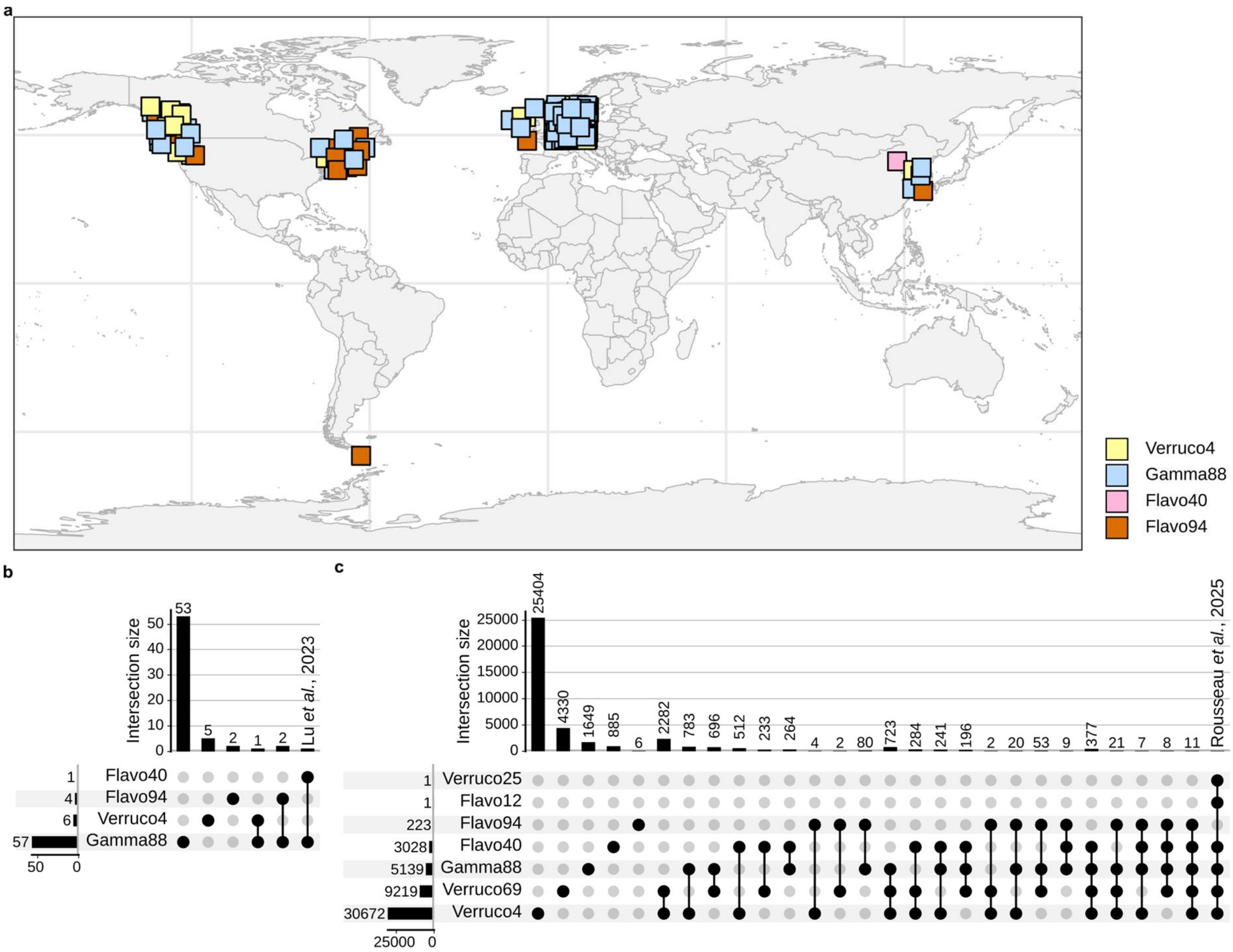


*Supplementary Fig. 2:* **Global distribution and co-occurrence of fucoidan-degrading isolates. a,** Geographic distribution of exact genome matches for representative degraders (Verruco4, Gamma88, Flavo40, Flavo94) across samples in the mOTUs database. Each square denotes a sample containing a genome matching the corresponding isolate. **b,** UpSet plot showing the number of samples in the mOTUs database containing exact genome matches to individual isolates or their combinations. Bars indicate intersection sizes and connected dots denote specific isolate combinations co-occurring within the same sample. **c,** UpSet plot showing the number of samples in the MicrobeAtlas database containing isolates or their combinations based on >99% 16S rRNA gene identity. Bars indicate intersection sizes and connected dots denote specific isolate combinations co-occurring within the same sample, highlighting co-occurrence patterns across environments.

**Supplementary Figure 3**

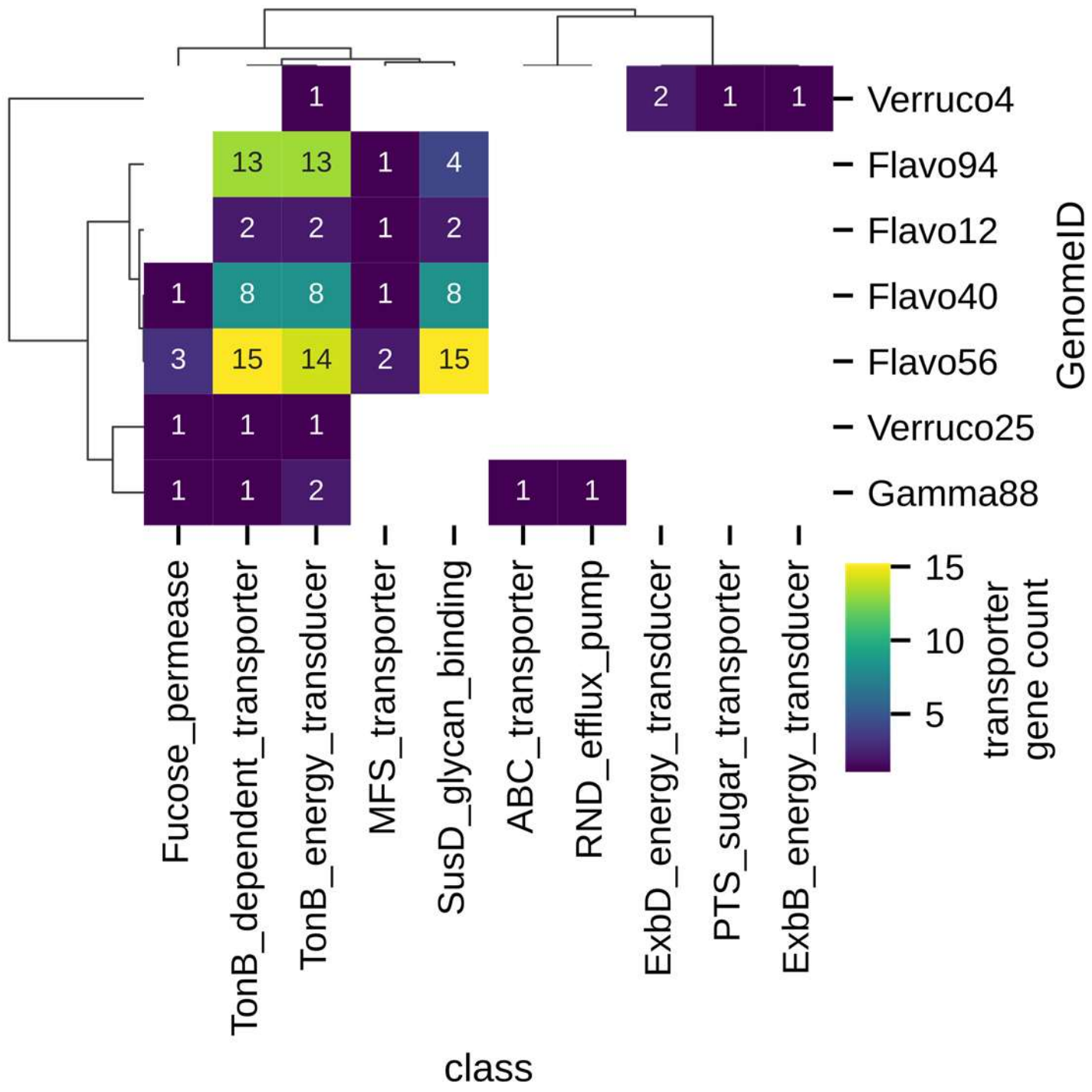


*Supplementary Fig. 3:* **Transporters co-localizing within PULs of isolated fucoidan degraders.** The heatmap shows the total number of different transporters that are found in fucoidan PULs of degraders. Dendrograms represent hierarchical clustering.

**Supplementary Figure 4**

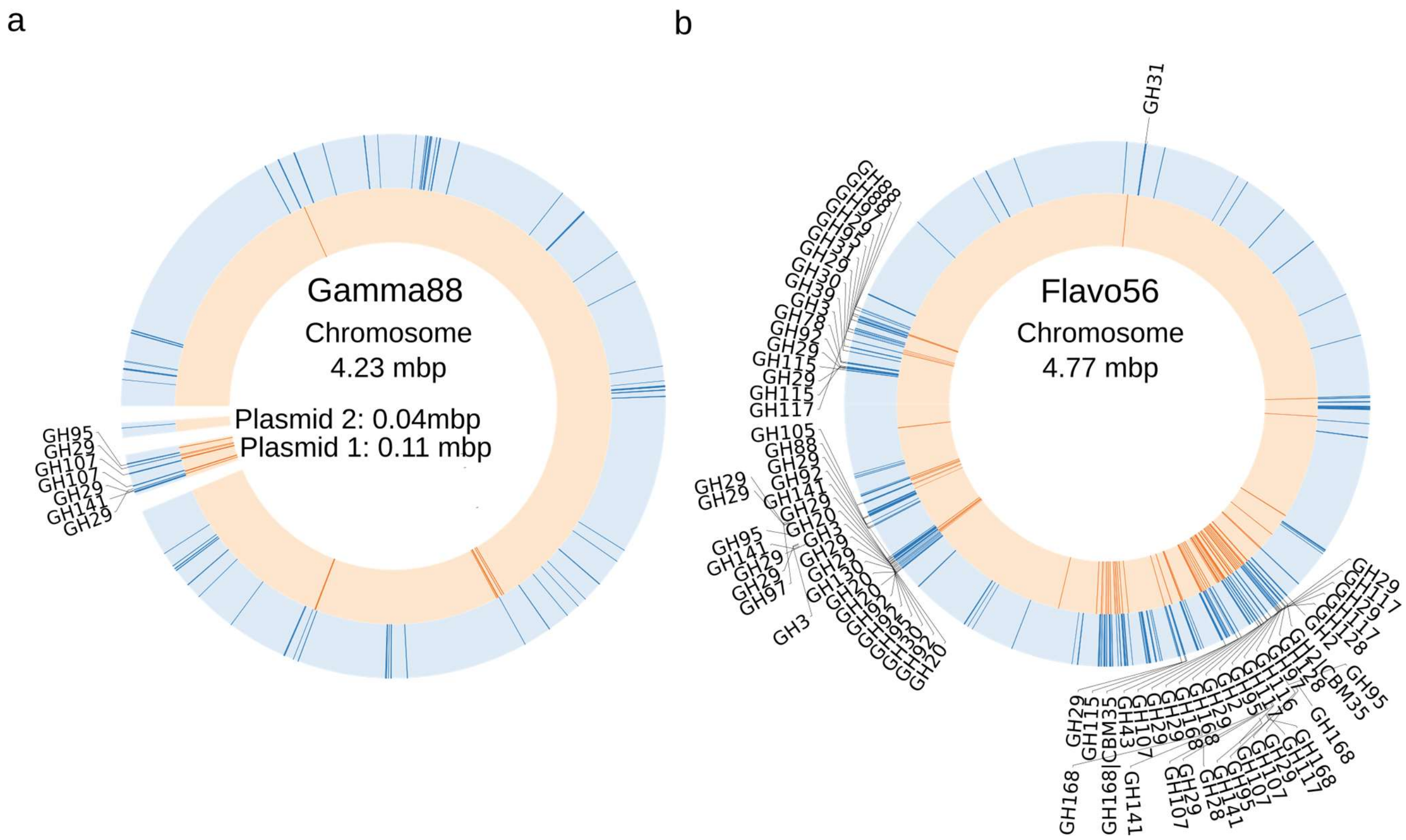


*Supplementary Fig. 4:* **Genomic localization of fucoidan-targeting CAZymes.** Circular genome maps of the isolated fucoidan degraders Gamma88 (**a**) and Flavo56 **(b)**, showing chromosomal and plasmid-encoded repertoires of carbohydrate-active enzymes (CAZymes). Coloured radial bars indicate the genomic positions of annotated CAZymes (blue) and sulfatases (orange). Glycoside hydrolases implicated in fucoidan degradation are annotated with family-level labels.

**Supplementary Figure 5**

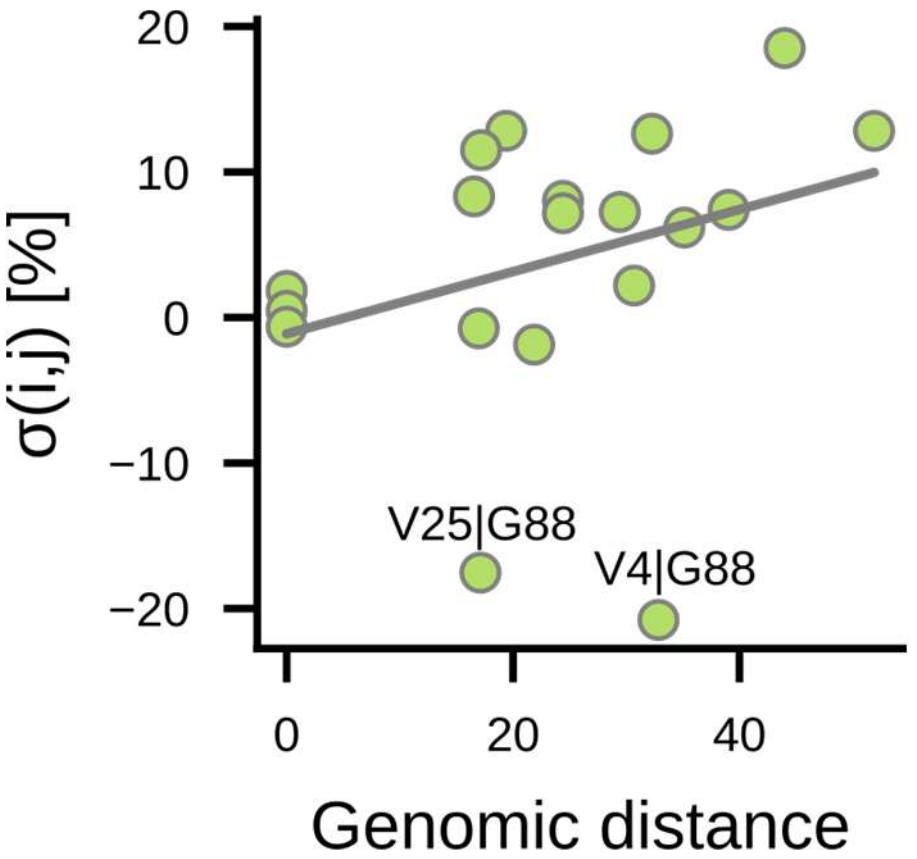


*Supplementary Fig. 5:* **Synergism scores in degrader co-cultures as a function of genomic distance.** Pairwise synergism scores (σ(i,j)) plotted against the Euclidean distance in the proportion of fucose and sulfate targeting enzymes relative to total fucoidan-associated enzymes. Each point represents the mean synergism score from three biological replicates for one of 24 degrader–degrader co-cultures (n=24 strain pairs) from the Verrucomicrobia-centric co-cultures. The grey line indicates an ordinary least-squares linear regression and the association was assessed using a two-sided Pearson correlation (r = 0.53, *P* = 0.02). Selected strain pairs are labelled. The co-cultures V4|G88 and V25|G88 are included in the analysis but fall outside the main trend.

**Supplementary Figure 6**

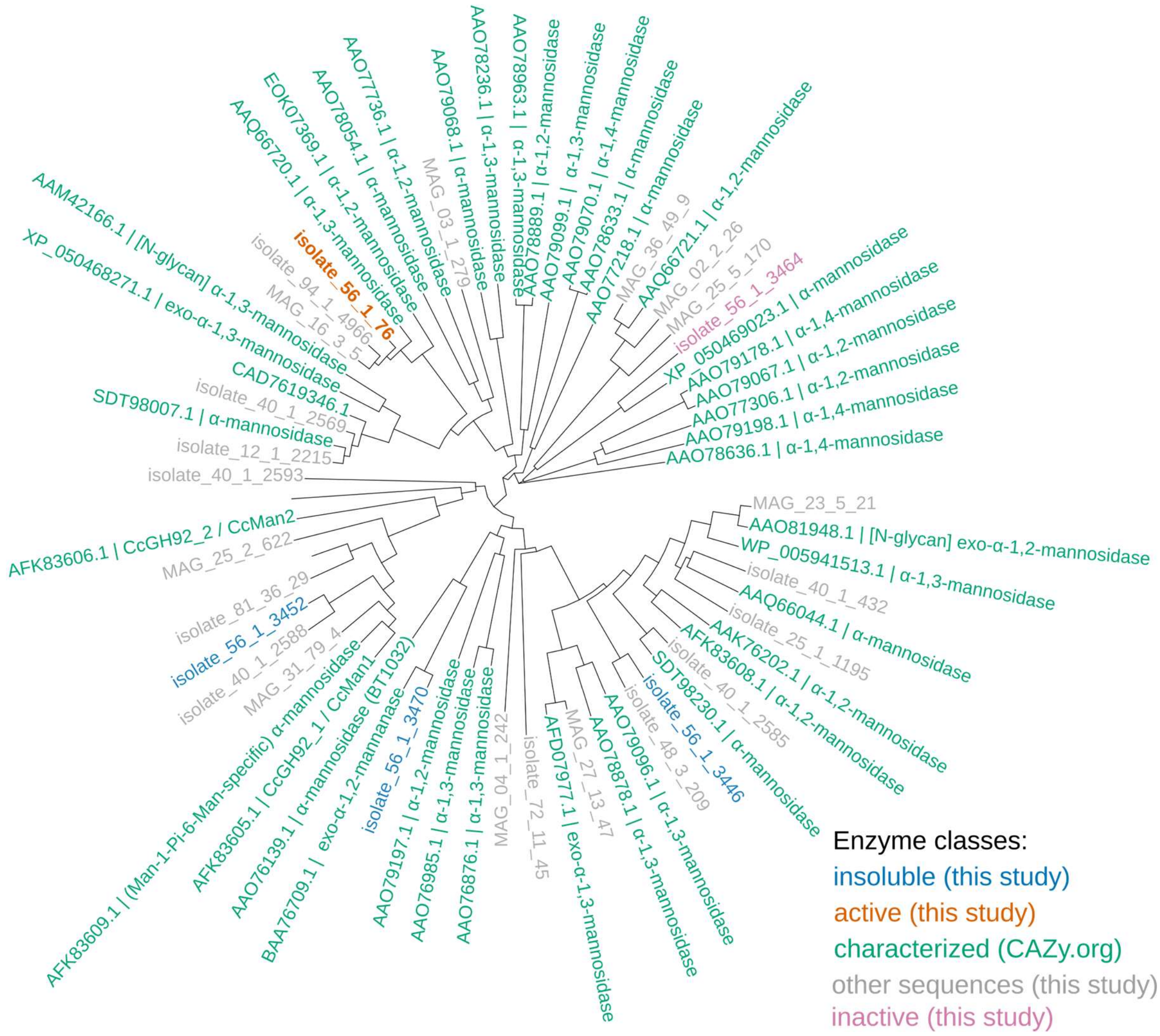


*Supplementary Fig. 6:* **Phylogenetic analysis of GH92 α-mannosidases.** Maximum-likelihood phylogenetic tree of GH92 family enzymes, including sequences from this study and all functionally characterized reference enzymes from CAZy. Sequences characterized in this study are classified based on experimental outcomes: active enzymes (orange), insoluble proteins (blue) and inactive enzymes (purple). Previously characterized enzymes are shown in green and additional sequences encoded by members of the fucoidan-degrading community are shown in grey. Functional annotations, where available, are indicated next to sequence labels. The tree was inferred from a multiple sequence alignment of catalytic domains using MAFFT, trimmed with trimAl and reconstructed with FastTree.

**Supplementary Figure 7**

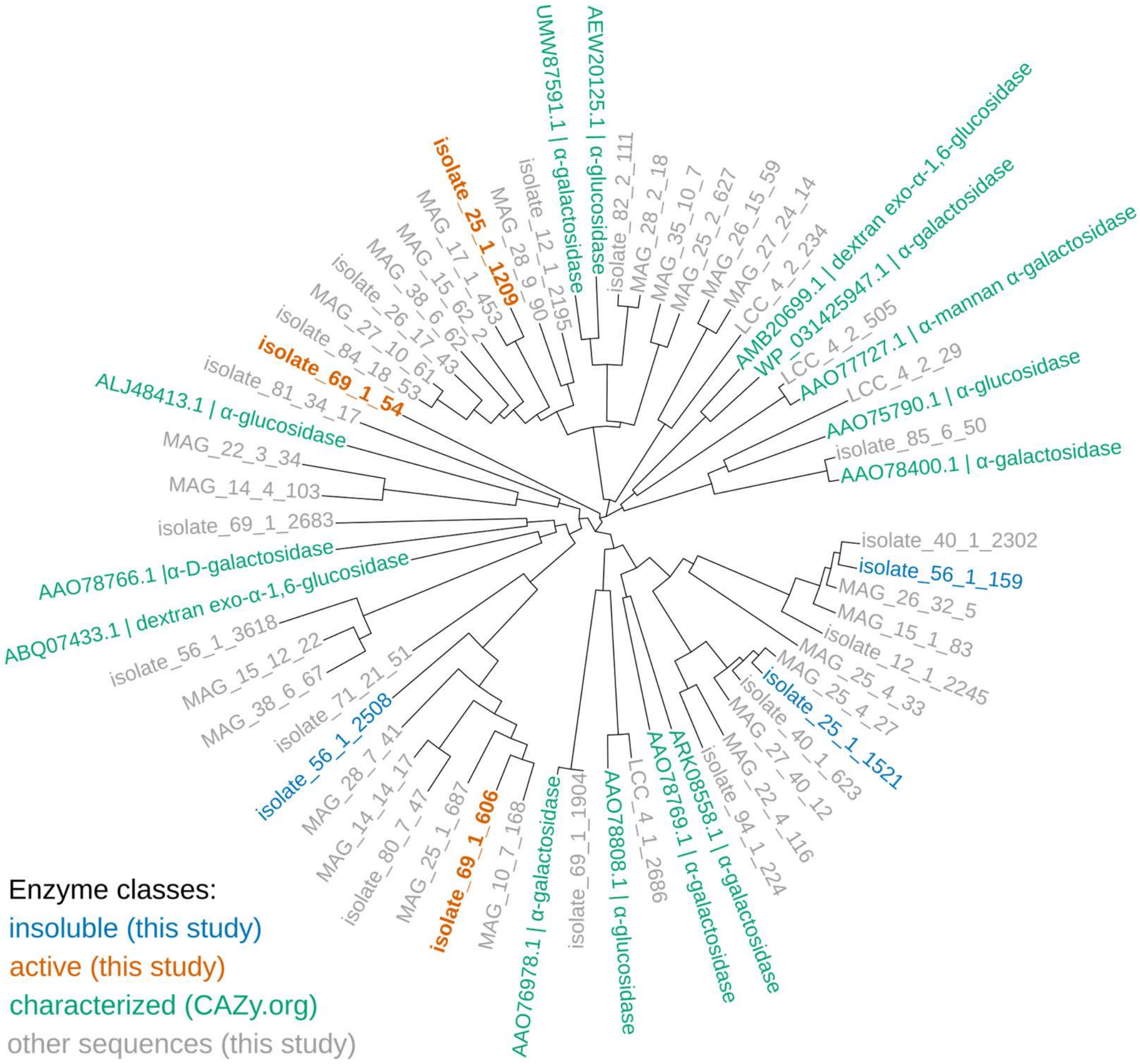


*Supplementary Fig. 7:* **Phylogenetic analysis of GH97 α-galactosidases.** Maximum-likelihood phylogenetic tree of GH97 family enzymes, including sequences from this study and all functionally characterized reference enzymes from CAZy. Sequences characterized in this study are classified based on experimental outcomes: active enzymes (orange), insoluble proteins (blue) and inactive enzymes (purple). Previously characterized enzymes are shown in green and additional sequences encoded by members of the fucoidan-degrading community are shown in grey. Functional annotations, where available, are indicated next to sequence labels. The tree was inferred from a multiple sequence alignment of catalytic domains using MAFFT, trimmed with trimAl and reconstructed with FastTree.

## Supplementary Figure 8

**a PUL_1|Flavo56**

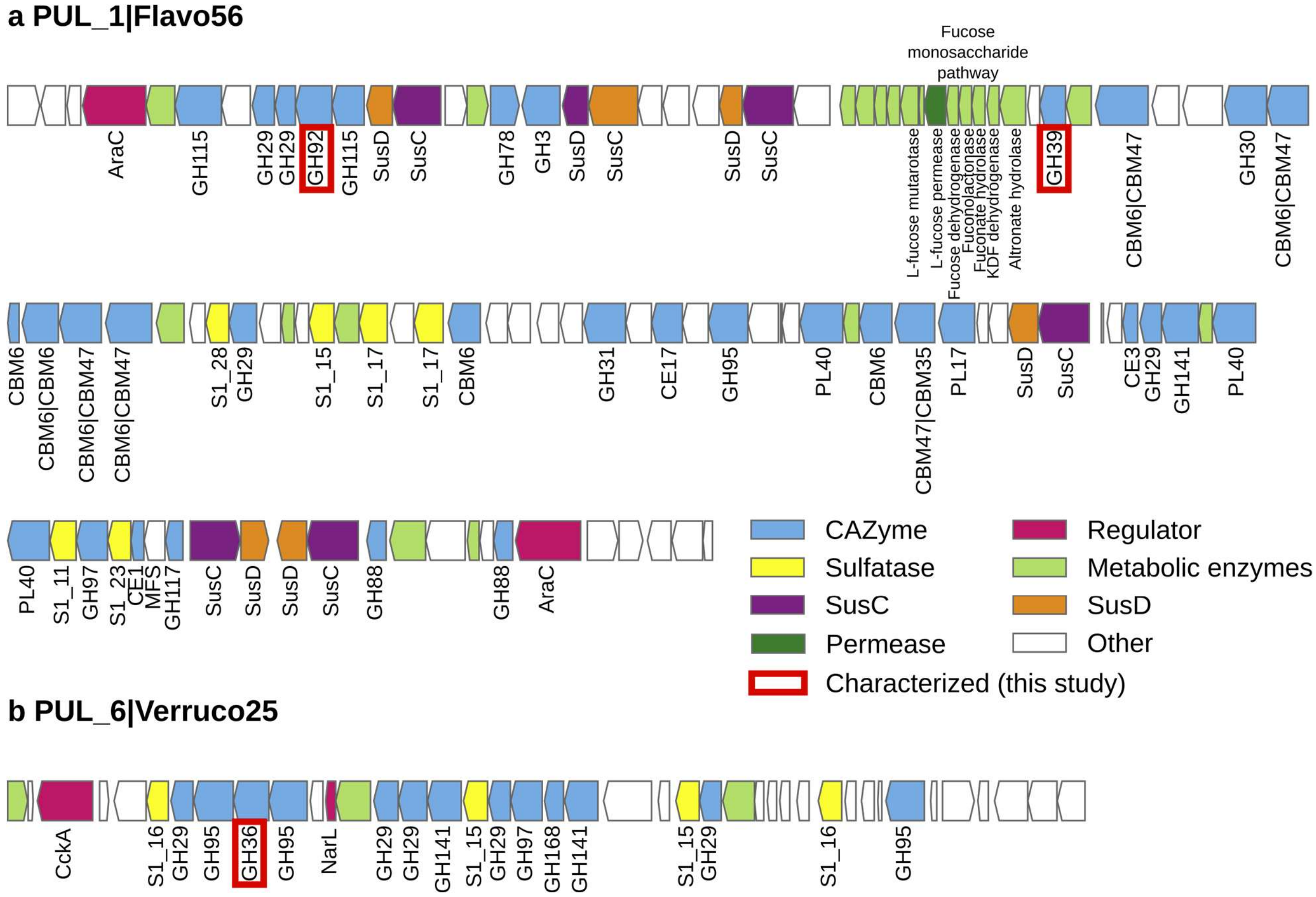


*Supplementary Fig. 8:* **Genetic organization of two fucoidan-targeting polysaccharide utilization loci (PULs).** Gene organization of two representative fucoidan-targeting PULs from Flavo56 (**a**) and Verruco25 (**b**). Arrows indicate open reading frames and their orientation. Genes are colour-coded by functional category: CAZymes (red), sulfatases (blue), transporters (teal), regulators (purple), metabolic enzymes (green) and other genes (grey). CAZymes and sulfatases implicated in fucoidan degradation are annotated with family-level labels. Enzymes biochemically characterized in this study are highlighted with orange outlines.

**Supplementary Figure 9**

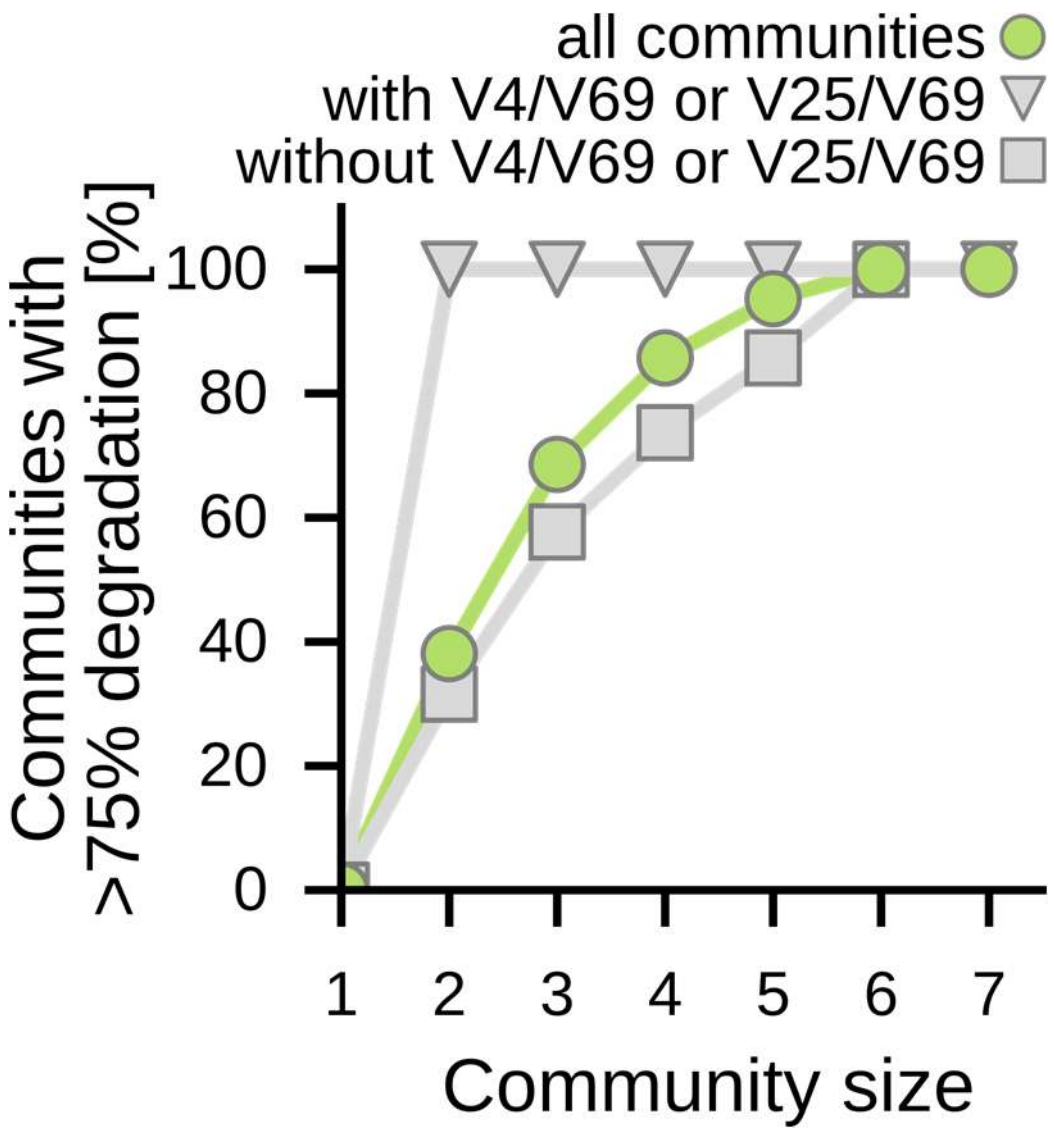


*Supplementary Fig. 9:* **Dependency of community function on high-performing strain pairs.** Percentage of communities achieving ≥75% fucoidan degradation across all 127 possible combinations of the seven degraders, grouped by community size. Green circles show all communities. Grey triangles show communities containing either of the high-performing pairs V4/V69 or V25/V69; grey squares show communities lacking both pairs. The 75% threshold was chosen to reflect degradation levels exceeding those of the best-performing monocultures.

## Supplementary Table Legends

### Supplementary Table 1

Sheet 1: Stock solutions used for media preparation.

Sheet 2: Media formulations used in this study.

### Supplementary Table 2

Sheet 1: Genome identifiers, accession numbers, MIMAG quality metrics, taxonomy, strain names and inferred functional roles.

Sheet 2: Relative abundances of genomes in metagenomes of enrichment cultures.

Sheet 3: TYGS-based classification of isolates.

### Supplementary Table 3

Sheet 1: Inferred activities and annotation confidence for putative fucoidan-degrading enzymes.

Sheet 2: Protein sequences of putative fucoidan-degrading enzymes.

Sheet 3: MMseqs2 clustering of fucoidan-degrading enzymes.

Sheet 4: Annotated fucoidan PULs in isolate genomes.

Sheet 5: Genomic specialization scores of fucoidan degraders.

### Supplementary Table 4

Sheet 1: Exact masses, fragment ions and retention times for LC–MS identification of fucoidan-derived monosaccharides.

Sheet 2: Time-resolved concentrations of acid-hydrolysed culture supernatants for eight degraders.

Sheet 3: Time-resolved concentrations of free monosaccharides in the culture supernatants of eight degraders.

Sheet 4: Sugar concentrations (mM), calculated degradation (%) and statistical significance for 29 strains in monoculture.

Sheet 5: Sugar concentrations (mM), calculated degradation (%) and statistical significance for 87 pairwise co-cultures.

Sheet 6: Sugar concentrations (mM), calculated degradation (%) and statistical significance for degraders grown on residual fucoidans from primary degraders.

Sheet 7: Sugar concentrations (mM), calculated degradation (%) and statistical significance for all 127 combinations of seven degraders grown on fucoidan.

Sheet 8: Sugar concentrations (mM), calculated degradation (%) and statistical significance for seven communities grown on nine fucoidans.

### Supplementary Table 5

Sheet 1: Growth parameters for 87 pairwise co-cultures.

Sheet 2: Fold-changes and statistical significance of fucoidan degradation in 87 pairwise co-cultures.

Sheet 3: Fold-changes and statistical significance of growth in 87 pairwise co-cultures.

Sheet 4: Synergism scores and statistical significance for 87 pairwise co-cultures.

**Supplementary Table 6**

Sheet 1: Primer sequences, linear ranges, limits of detection and recovery for strain-specific qPCR assays.

Sheet 2: Quantified bacterial DNA in degrader–degrader and degrader–exploiter co-cultures.

**Supplementary Table 7**

Sheet 1: Names, accession numbers, construct sequences and molecular characteristics of recombinant glycoside hydrolases.

**Supplementary Table 8**

Sheet 1: Observed and predicted degradation values used for model training and testing.

Sheet 2: Observed and predicted degradation values for out-of-sample predictions across diverse fucoidans.

**Supplementary Table 9**

Sheet 1: Sulfate content, total hydrolysable carbohydrate concentration, estimated fucoidan content and mass recovery of nine fucoidans. Values are reported as mean ± s.d. of three independent measurements.

**Gel source data**

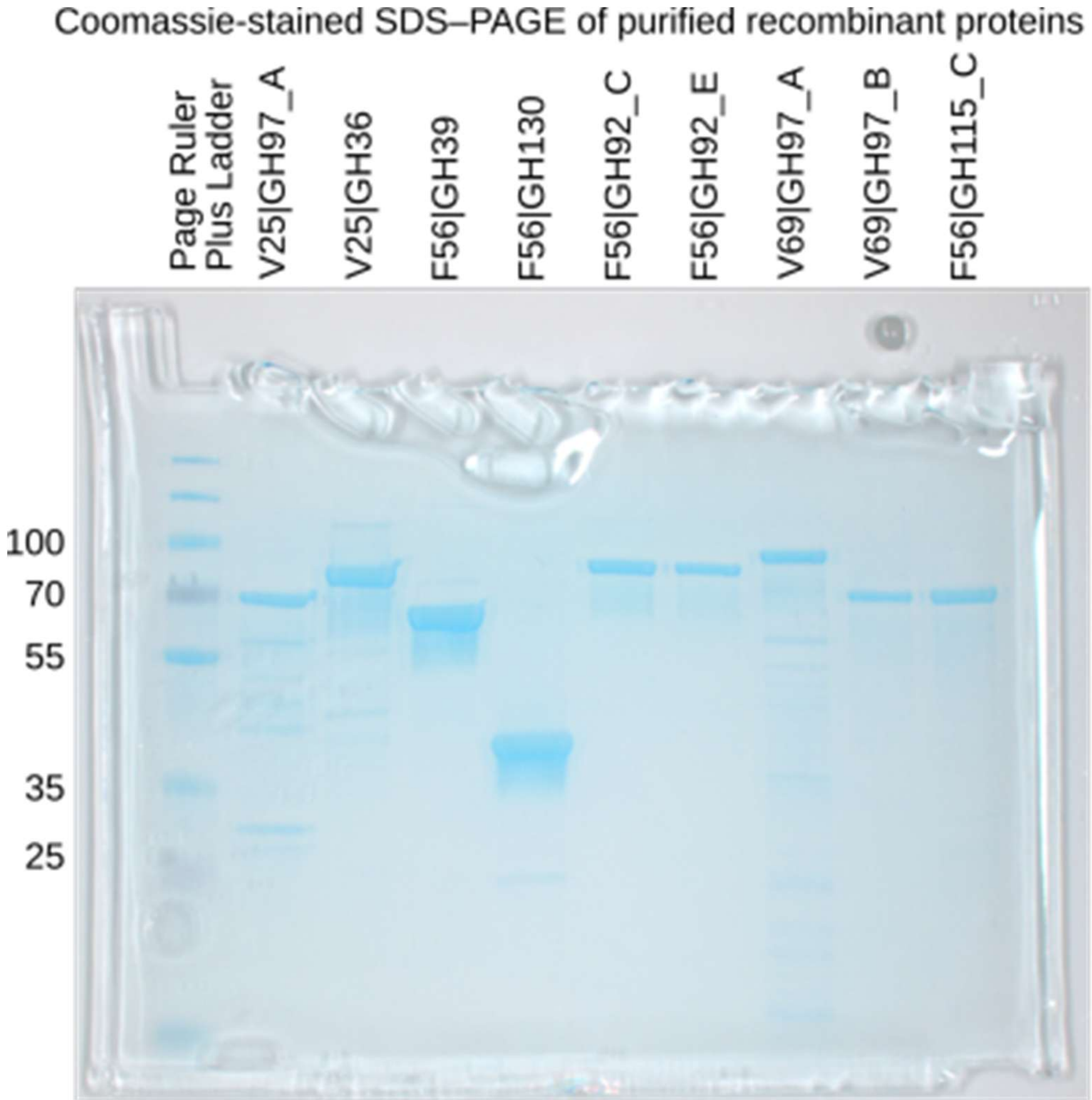


*Gel source data 1:* **Uncropped SDS-PAGE of fucoidan-acting enzymes characterized in this study.** Purified proteins used for Fig. 2h and Extended Data Fig. 7. The molecular weight (in kDa) of the protein ladder is indicated as text labels.

84. Ponce, N. M. A. & Stortz, C. A. A comprehensive and comparative analysis of the fucoidan compositional data across the Phaeophyceae. *Front. Plant Sci.* **11**, 556312 (2020).

85. Kopplin, G. *et al.* Structural characterization of fucoidan from *Laminaria hyperborea*: assessment of coagulation and inflammatory properties and their structure–function relationship. *ACS Appl. Bio Mater.* **1**, 1880–1892 (2018).

86. Anastyuk, S. D., Shevchenko, N. M., Nazarenko, E. L., Dmitrenok, P. S. & Zvyagintseva, T. N. Structural analysis of a fucoidan from the brown alga *Fucus evanescens* by MALDI-TOF and tandem ESI mass spectrometry. *Carbohydr. Res.* **344**, 779–787 (2009).

87. Bilan, M. I., Grachev, A. A., Shashkov, A. S., Nifantiev, N. E. & Usov, A. I. Structure of a fucoidan from the brown seaweed *Fucus serratus* L. *Carbohydr. Res.* **341**, 238–245 (2006).

88. Meier-Kolthoff, J. P. & Göker, M. TYGS is an automated high-throughput platform for state-of-the-art genome-based taxonomy. *Nat. Commun.* **10**, 2182 (2019).

89. Ruscheweyh, H.-J. *et al.* Cultivation-independent genomes greatly expand taxonomic-profiling capabilities of mOTUs across various environments. *Microbiome* **10**, 212 (2022).

90. Anastyuk, S. D., Shevchenko, N. M., Dmitrenok, P. S. & Zvyagintseva, T. N. Structural similarities of fucoidans from brown algae *Silvetia babingtonii* and *Fucus evanescens*, determined by tandem MALDI-TOF mass spectrometry. *Carbohydr. Res.* **358**, 78–81 (2012).

91. Bilan, M. I. *et al.* Structure of a fucoidan from the brown seaweed *Fucus evanescens* C.Ag. *Carbohydr. Res.* **337**, 719–730 (2002).

92. Zvyagintseva, T. N. *et al.* Water-soluble polysaccharides of some far-eastern brown seaweeds. Distribution, structure, and their dependence on the developmental conditions. *J. Exp. Mar. Biol. Ecol.* **294**, 1–13 (2003).

93. Amin, M. N. G., Mischnick, P., Rosenau, T. & Böhmdorfer, S. Refined linkage analysis of the sulphated marine polysaccharide fucoidan of *Cladosiphon okamuranus* with a focus on fucose. *Carbohydr. Polym.* **342**, 122302 (2024).

94. Sakai, T., Ishizuka, K., Shimanaka, K., Ikai, K. & Kato, I. Structures of oligosaccharides derived from *Cladosiphon okamuranus* fucoidan by digestion with marine bacterial enzymes. *Mar. Biotechnol.* **5**, 536–544 (2003).

95. Yang, Y., Hu, T., Li, J., Xin, M. & Zhao, X. Structural characterization and effect on leukopenia of fucoidan from *Durvillaea antarctica*. *Carbohydr. Polym.* **256**, 117529 (2021).
