# Supplementary material for: Synergistic degradation of fucoidans in the ocean
Source: Nature. 2026 Aug 26;658(8136):682–9. doi: 10.1038/s41586-026-10980-z (PMC13668718; doi:10.1038/s41586-026-10980-z)
Supplement: Supplementary file 3 [file 41586_2026_10980_MOESM2_ESM.pdf]

nature portfolio

Corresponding author(s): Prof. Uwe Sauer & Prof. Otto Cordero

Last updated by author(s): Jul 20, 2026

Reporting Summary

Nature Portfolio wishes to improve the reproducibility of the work that we publish. This form provides structure for consistency and transparency in reporting. For further information on Nature Portfolio policies, see our Editorial Policies and the Editorial Policy Checklist.

Statistics

For all statistical analyses, confirm that the following items are present in the figure legend, table legend, main text, or Methods section.

n/a	Confirmed	
☐	☒	The exact sample size (*n*) for each experimental group/condition, given as a discrete number and unit of measurement
☐	☒	A statement on whether measurements were taken from distinct samples or whether the same sample was measured repeatedly
☐	☒	The statistical test(s) used AND whether they are one- or two-sided<br>*Only common tests should be described solely by name; describe more complex techniques in the Methods section.*
☒	☐	A description of all covariates tested
☐	☒	A description of any assumptions or corrections, such as tests of normality and adjustment for multiple comparisons
☐	☒	A full description of the statistical parameters including central tendency (e.g. means) or other basic estimates (e.g. regression coefficient) AND variation (e.g. standard deviation) or associated estimates of uncertainty (e.g. confidence intervals)
☐	☒	For null hypothesis testing, the test statistic (e.g. *F*, *t*, *r*) with confidence intervals, effect sizes, degrees of freedom and *P* value noted<br>*Give P values as exact values whenever suitable.*
☒	☐	For Bayesian analysis, information on the choice of priors and Markov chain Monte Carlo settings
☒	☐	For hierarchical and complex designs, identification of the appropriate level for tests and full reporting of outcomes
☐	☒	Estimates of effect sizes (e.g. Cohen's *d*, Pearson's *r*), indicating how they were calculated

Our web collection on statistics for biologists contains articles on many of the points above.

Software and code

Policy information about availability of computer code

Data collection	Metabolomics data were acquired using Analyst Software 1.7.3 (Sciex). Sequencing data were generated using an Illumina NovaSeq 6000, an Illumina NextSeq 500 and an Oxford Nanopore PromethION.
Data analysis	Python v3.9.12 (matplotlib, pandas, numpy, scipy), R v4.3.3 (ggplot2, rnaturalearth and sf)) Julia v1.12.3 (LsqFit.jl), OrthoANIu v1.2, SPAdes v3.13.0, CONCOCT v1.0.0, MaxBin v2.2.7, DAS Tool v1.1.2, Unicycler v0.4.8, CheckM v1.1.2, GTDB-Tk v2.0.0, dRep v3.5.0, DRAM v1.2.4, HMMER v3.3, MMseqs2 v13.4511, diamond v0.9.14.115, MAFFT v7.505, trimAl build 2019-08-05, FastTree v2.1.11, pyBarrnap v0.5.1, mOTUs v1, TYGS (access date 2024-03-2), Skyline v24.1.0.414.<br>Custom code generated in this study is available at:<br>https://github.com/EnvSysMicroLAB/Sichert2025_Fucoidan<br>https://github.com/tpriest0/Global_ocean_microbial_fucoidan_degrader_analysis

For manuscripts utilizing custom algorithms or software that are central to the research but not yet described in published literature, software must be made available to editors and reviewers. We strongly encourage code deposition in a community repository (e.g. GitHub). See the Nature Portfolio guidelines for submitting code & software for further information.

Data

Policy information about availability of data

All manuscripts must include a data availability statement. This statement should provide the following information, where applicable:

- Accession codes, unique identifiers, or web links for publicly available datasets
- A description of any restrictions on data availability
- For clinical datasets or third party data, please ensure that the statement adheres to our policy

All data supporting the findings of this study are available within the paper and its Supplementary Information. Raw LC–MS data are available via Panorama Public at https://doi.org/10.6069/r2z3-7b10. Genome assemblies and associated sequencing data have been deposited in the NCBI Sequence Read Archive under accession number PRJNA996876. Accession numbers for all genomes used in this study are listed in Supplementary Table 2. All data underlying the analysis of global ocean metagenomes, including fucoidan degrader annotations, PUL sequences, genome summaries and community profiles, are publicly available on Zenodo (https://zenodo.org/records/19591477) and https://github.com/tpriest0/Global_ocean_microbial_fucoidan_degrader_analysis.
Publicly available databases used in this study were: the CAZy database (http://www.cazy.org; accessed September 2022 and March 2026), dbCAN v10 and v14 (https://bcb.unl.edu/dbCAN2/), SulfAtlas v1.2 (https://sulfatlas.sb-roscoff.fr/), Pfam (https://www.ebi.ac.uk/interpro/), MetaCyc (https://metacyc.org/), KEGG (https://www.genome.jp/kegg/), GTDB release 202 (https://gtdb.ecogenomic.org/), the mOTUs database (https://motu-tool.org/) and the MicrobeAtlas database (https://microbeatlas.org/).No data in this study are subject to restricted or controlled access.

Research involving human participants, their data, or biological material

Policy information about studies with human participants or human data. See also policy information about sex, gender (identity/presentation), and sexual orientation and race, ethnicity and racism.

Reporting on sex and gender	N/A
Reporting on race, ethnicity, or other socially relevant groupings	N/A
Population characteristics	N/A
Recruitment	N/A
Ethics oversight	N/A

Note that full information on the approval of the study protocol must also be provided in the manuscript.

Field-specific reporting

Please select the one below that is the best fit for your research. If you are not sure, read the appropriate sections before making your selection.

☒ Life sciences ☐ Behavioural & social sciences ☐ Ecological, evolutionary & environmental sciences

For a reference copy of the document with all sections, see nature.com/documents/nr-reporting-summary-flat.pdf

Life sciences study design

All studies must disclose on these points even when the disclosure is negative.

Sample size	No formal sample size calculation was performed. All experiments used a minimum of three biological replicates ($n \geq 3$), i.e. independent growth cultures, which is standard in microbial degradation studies and sufficient to detect consistent differences in fucoidan degradation given the low variability between replicates. Sample sizes for co-culture and time-series experiments were additionally based on effect sizes from preliminary experiments and constrained by throughput.
Data exclusions	No data was excluded from the analyses.
Replication	All experiments included independent biological replicates (typically $n = 3$). Replication success was evaluated based on consistency across independent cultures. No replication attempts failed.
Randomization	Metabolomics samples were measured in randomized order to minimize technical bias. Randomization was not applicable to the in vitro experiments because in vitro cultures were genetically identical and grown under identical conditions, leaving no confounding variables to randomize.
Blinding	During LC–MS data acquisition, sample identity was blinded to the analyst through randomized filenames, and for hierarchical clustering, principal component analyses and model fitting, group identities were assigned only after data processing was complete. Blinding during culturing was not applicable because degradation and growth were quantified by automated, objective instrument readouts (OD_{600} and LC–MS) rather than subjective assessment, leaving no scope for experimenter bias.

Reporting for specific materials, systems and methods

We require information from authors about some types of materials, experimental systems and methods used in many studies. Here, indicate whether each material, system or method listed is relevant to your study. If you are not sure if a list item applies to your research, read the appropriate section before selecting a response.

Materials & experimental systems

n/a	Involved in the study
☒	☐ Antibodies
☒	☐ Eukaryotic cell lines
☒	☐ Palaeontology and archaeology
☒	☐ Animals and other organisms
☒	☐ Clinical data
☒	☐ Dual use research of concern
☒	☐ Plants

Methods

n/a	Involved in the study
☒	☐ ChIP-seq
☒	☐ Flow cytometry
☒	☐ MRI-based neuroimaging

Plants

Seed stocks	N/A
Novel plant genotypes	N/A
Authentication	N/A
